# Acrylate-Induced β-H Elimination in Coordination Insertion Copolymerizaton Catalyzed by Nickel

**DOI:** 10.1021/jacs.3c10800

**Published:** 2023-11-22

**Authors:** Shuoyan Xiong, Alexandria Hong, Priyabrata Ghana, Brad C. Bailey, Heather A. Spinney, Hannah Bailey, Briana S. Henderson, Steve Marshall, Theodor Agapie

**Affiliations:** † Division of Chemistry and Chemical Engineering, California Institute of Technology, Pasadena, California 91125, United States; ‡ Chemical Science, Core R&D, The Dow Chemical Company, Midland, Michigan 48667, United States

## Abstract

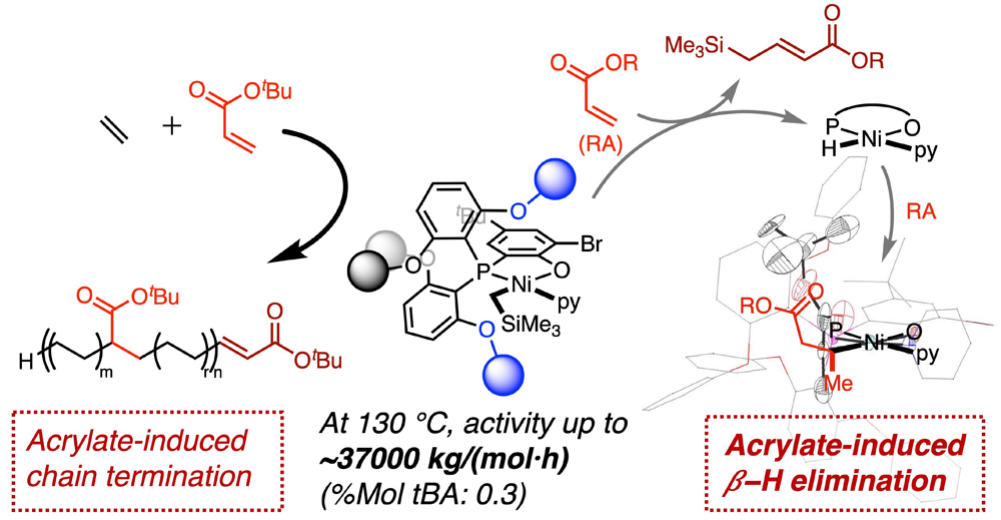

Polar monomer-induced β-H elimination is a key elementary step in polar polyolefin synthesis by coordination polymerization but remains underexplored. Herein, we show that a bulky neutral Ni catalyst, **1**^**Ph**^, is not only a high-performance catalyst in ethylene/acrylate copolymerization (activity up to ∼37,000 kg/(mol·h) at 130 °C in a batch reactor, mol % tBA ∼ 0.3) but also a suitable platform for investigation of acrylate-induced β-H elimination. **4**^**Ph-**^***t***^**Bu**^, a novel Ni alkyl complex generated after acrylate-induced β-H elimination and subsequent acrylate insertion, was identified and characterized by crystallography. A combination of catalysis and mechanistic studies reveals effects of the acrylate monomer, bidentate ligand, and the labile ligand (e.g., pyridine) on the kinetics of β-H elimination, the role of β-H elimination in copolymerization catalysis as a chain-termination pathway, and its potential in controlling the polymer microstructure in polar polyolefin synthesis.

## Introduction

**Figure 1:**
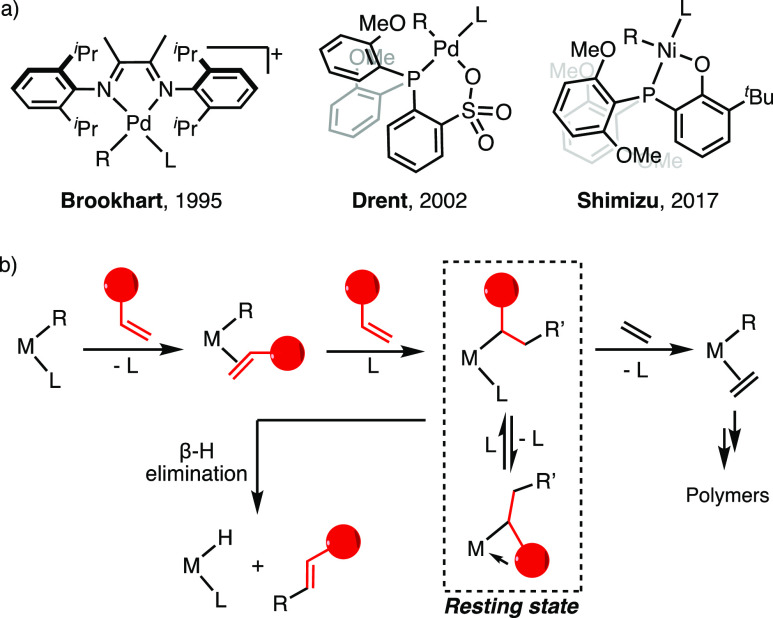
(a) Examples of catalysts for copolymerization of ethylene and polar monomers; (b) mechanism of coordination copolymerization, the resting state, and example of termination event involving β-H elimination [R, R′: H, alkyl or polymer chain; L: olefin, or the labile ligand (e.g., pyridine); and red circle: polar group].

Polyolefins account for over half of global plastic production.^1–8^ Coordination copolymerization of nonpolar and polar monomers is of high interest as it can provide value-added functional polyolefins with diverse but controlled material properties and potential degradability.^2–7,9–36^ However, industrial implementation of this process is limited by low activity [typically <1000 kg/(mol·h)] and thermal stability (typically <100 °C) of reported catalysts (Figure 1a), as well as the low-molecular weight (MW) of the resulting copolymers.^10,17,19,37–40^

In ethylene/polar monomer copolymerization, the intermediate generated after polar monomer insertion is typically the resting state of catalysis due to coordination of the enchained polar group to a vacant site on the transition metal and challenges of olefin insertion into a secondary metal-alkyl bond (Figure 1b).^37,41–49^ β-H elimination from this intermediate is thus a key elementary step controlling catalyst performance and polymer microstructure.^37,49^ For ethylene and α-olefin polymerization, β-H elimination has been investigated intensively, and relevant intermediates including β-agostic species have been identified and characterized.^44,50–61^ On the other hand, limited studies have been reported of intermediates relevant to polar monomer-induced β-H elimination occurring during catalysis.^46,47,62,63^ The only catalyst species resulting from a β-H elimination event characterized by crystallography is an intermediate generated after acrylate insertion and chain walking with a cationic Pd complex.^64^ Prior studies were also carried out on catalyst systems that exhibit no or low reactivity in ethylene/polar monomer copolymerization [e.g., activity <20 kg/(mol·h)].

Nickel catalysts have been a recent focus in copolymerization involving polar monomers due to nickel’s relatively low cost and promising performance.^17–19^ Despite the high interest, β-H elimination at nickel has not been thoroughly studied, potentially due to the lack of a suitable catalyst system that undergoes facile β-H elimination while still being productive in copolymerization. Herein we report highly active Ni phosphine phenoxide catalysts and their β-H elimination behavior. An intermediate, **4**^**Ph-**^***t***^**Bu**^, generated from a putative Ni-hydride, was characterized by X-ray crystallography. These results provide insights into how catalyst design impacts catalyst activity, copolymer Mw, and chain-end functionality in polar polyolefin synthesis.

## Results and Discussion

### Catalyst Design, Preparation, and Characterization

**Figure 2:**
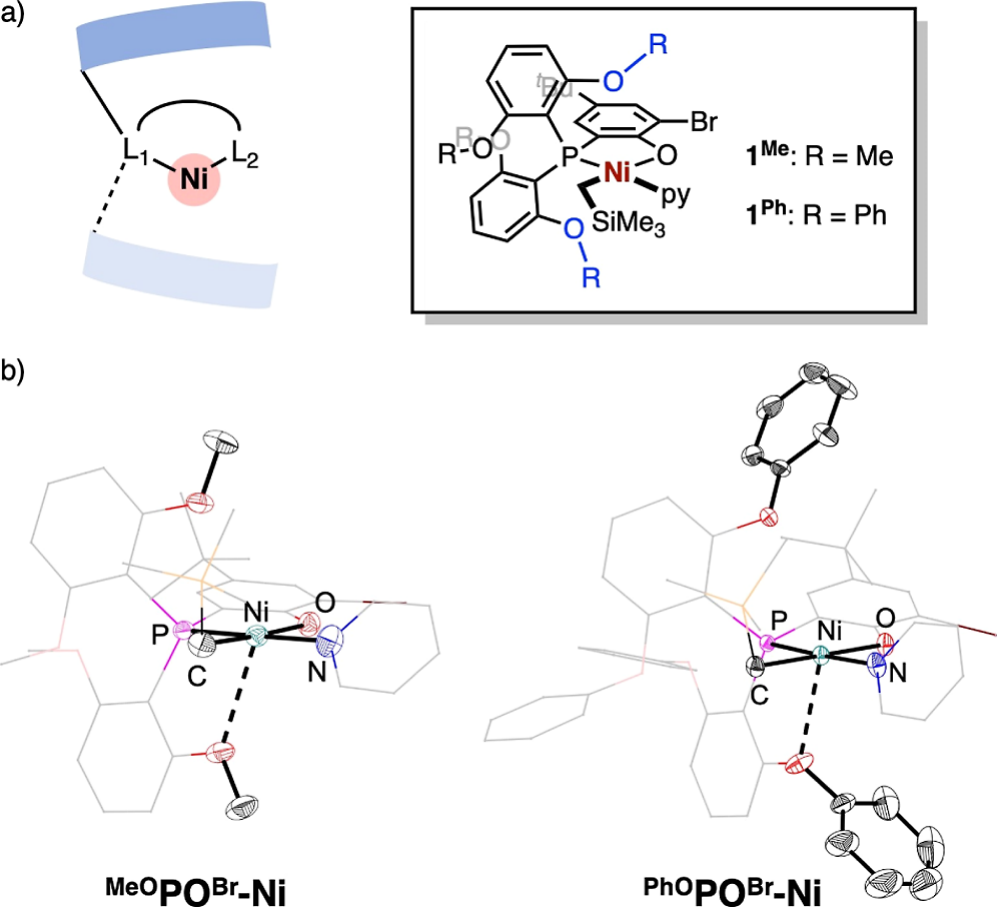
(a) Depiction of the steric profile of the catalyst system and two new catalysts in this work. (b) Solid-state structures of **1**^**Me**^ and **1**^**Ph**^ with thermal ellipsoid representations emphasizing the metal coordination sphere and the steric profile of groups along the axial positions.

The nickel complexes generated after β-H elimination of a copolymer chain are expected to be highly reactive toward further insertion or catalyst decomposition reactions. Previous mechanistic studies have identified several catalyst deactivation pathways starting from inter- and intramolecular interactions axial to the nickel center.^45–47,65–67^ To stabilize reactive intermediates, a catalyst design strategy targeting large axial shielding was chosen (Figure 2a). Increasing proximal steric hindrance has also shown promise in improving catalytic activity and thermal stability in Ni catalysts supported by anionic PO ligands.^22,37,40,68–73^ Two neutral Ni complexes, **1**^**Me**^ and **1**^**Ph**^, were synthesized as single-component catalysts for ethylene/acrylate copolymerization and precursors for the investigation of β-H elimination (Figure 2a). Structural characterization by single-crystal X-ray diffraction (scXRD), in combination with topographical steric analysis by Cavallo’s SambVca 2.1,^74,75^ confirms that axial positions of the Ni center in both complexes are covered from both the top and bottom directions (Figures 2b and Figure S1). Notably, the phenoxy group in **1**^**Ph**^ also provides steric shielding extending to the side of the O side, while the methoxy group in **1**^**Me**^ provides steric shielding only on the P side.

### High-Temperature Ethylene/Acrylate Copolymerization

Both **1**^**Me**^ and **1**^**Ph**^ are highly active in ethylene/acrylate copolymerization reactions (Table 1, entries 1–9). The bulkier catalyst, **1**^**Ph**^, shows significantly higher activity than **1**^**Me**^ but produces copolymers with lower tBA incorporation (e.g., entry 2 vs 6), consistent with structure–performance relationships of Ni catalysts reported previously.^22,34,40^ The optimized reaction temperature for **1**^**Me**^ is 90 °C (entry 1 vs 2, 3 vs 5) while **1**^**Ph**^ is significantly more active at 110 °C than that at 90 °C (entry 7 vs 9). The latter is also in contrast with the optimized reaction temperatures for other reported Pd and Ni catalysts, typically ranging between 50 and 90 °C (Figure S13).^22,40,49,70^ An activity of ∼33,000 kg/(mol·h) was achieved at 110 °C in a batch reactor (entry 10), demonstrating *a* ∼ 10 times increase compared to the state-of-art activity of Ni phosphine phenoxide catalysts, with a similar level of acrylate incorporation (0.3%).^34^ Overall, **1**^**Ph**^ features significantly improved activity and thermal stability compared to reported catalysts (Supporting Information Section S6, Figures S12 and S13). At 130 °C, **1**^**Ph**^ shows an activity of ∼37,000 kg/(mol·h) in a batch reactor (entry 8), albeit with low tBA incorporation (0.3 mol %). To the best of our knowledge, this is the first reported example of ethylene/acrylate coordination copolymerization at >110 °C. These results show promise for potential practical applications as low catalyst activity, low catalyst thermal stability, and low copolymer MW are three major limitations to industrial implementation.^17,76^

**Table 1:** Ethylene/Acrylate Copolymerization Results

entry^a^	catalyst	*T*/°C	[tBA]/M	activity^b^	*M*_w_/10^3^	*Đ*	% mol tBA	*T*_m_/°C
1	**1**^**Me**^	70	0.05	750	120.0	2.6	2.3	113
2	**1**^**Me**^	90	0.05	1550	73.3	2.4	1.5	115
3	**1**^**Me**^	90	0.10	720	47.0	2.2	3.4	106
4	**1**^**Me**^	90	0.15	410	35.4	2.3	4.8	99
5	**1**^**Me**^	110	0.10	440	17.8	2.4	2.9	107
6	**1**^**Ph**^	90	0.05	21,000	38.5	2.3	0.3	126
7	**1**^**Ph**^	90	0.10	9700	32.9	2.4	0.7	123
8	**1**^**Ph**^	90	0.15	5700	30.0	2.3	1.0	120
9	**1**^**Ph**^	110	0.10	17,800	26.0	2.4	0.7	123
10^c^	**1**^**Ph**^	110	0.054	33,000	28.4	2.2	0.3	127
11^c^	**1**^**Ph**^	110	0.108	14,000	24.9	2.2	0.6	125
12^c^	**1**^**Ph**^	130	0.054	37,000	15.6	2.6	0.3	127

a Entries 1–9: copolymerization in high-throughput parallel pressure reactors. [Ni] = 0.05 mM, ethylene pressure = 400 psi, toluene solvent, *V* = 5 mL. Polymerization was stopped after consuming a set amount of ethylene, and each entry represents multiple replicated runs. See Supporting Information Section S3 for detailed procedures and Table S2 for original catalytic runs.

b kg/(mol·h).

c Entries 10–12: copolymerization in a batch reactor: V (solvent) = 550 mL, [Ni] = 0.043 mM, ethylene pressure = 430 psi, *t* = 3.5 min (entry 6), 6.5 min (entry 7), or 3 min (entry 8), ethylene consumption = 40 g. See Supporting Information Section S3 for detailed procedures.

### Identification of β-H Elimination and Subsequent Acrylate Insertion

**Figure 3:**
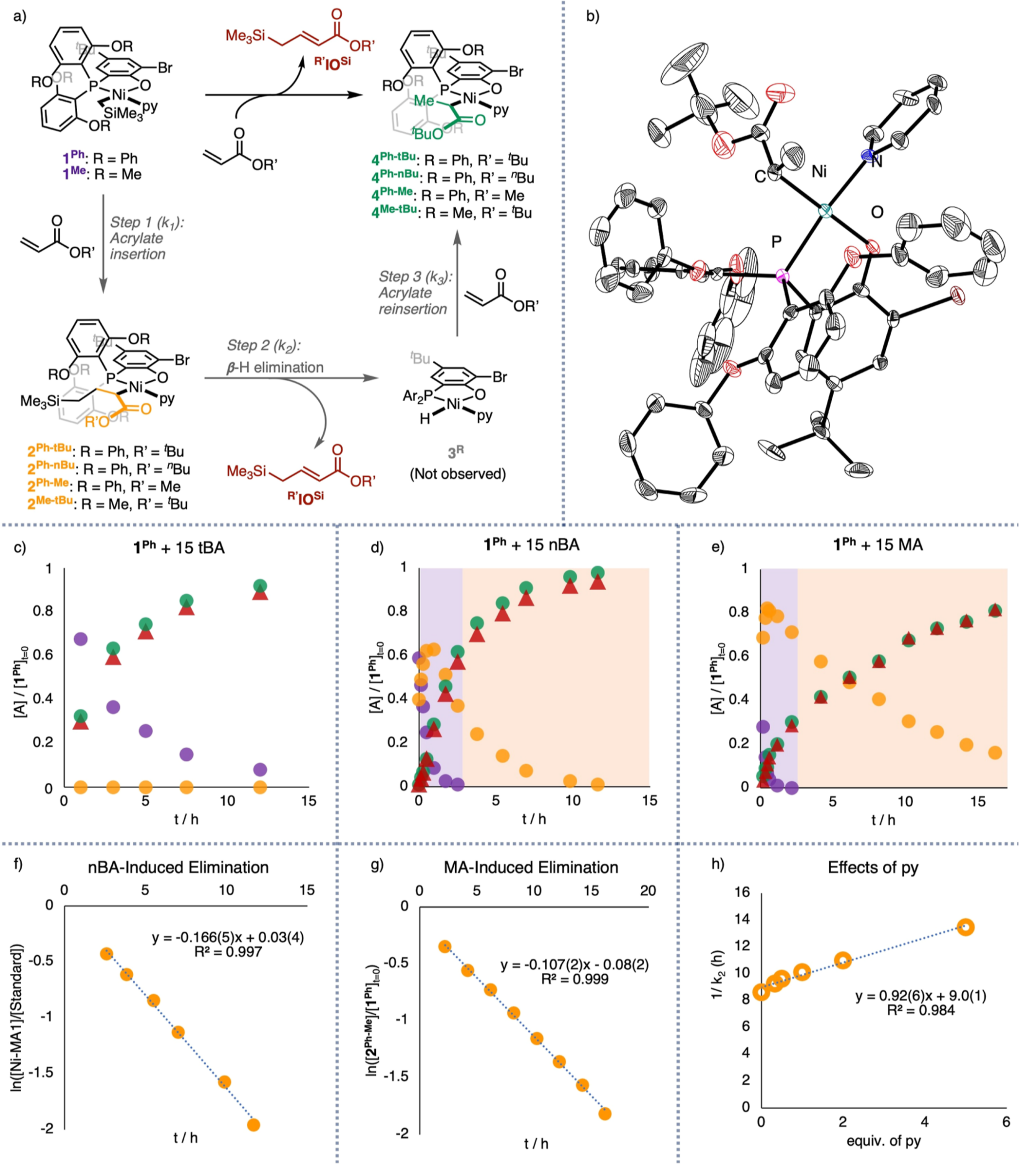
(a) Generation of the internal olefin (**IO**^**Si**^) and the acrylate-inserted species via a three-step pathway. (b) Solid-state structure of **4**^**Ph-**^***t***^**Bu**^. (c–e) Kinetic profiles of reaction of tBA, nBA, and MA with **1**^**Ph**^. (f,g) *pseudo*-first-order kinetics of acrylate-induced β-H elimination revealed by log plots for the decay of **2**^**Ph-R′**^. Note: Colors in (c∼h) correspond to coloring of structures in (a): purple for the starting catalyst complex, yellow for the acrylate-inserted Ni–alkyl complex, red for the β-H elimination product, and green for the acrylate-inserted Ni–H complex. Condition for (c–g): [Ni] = [**1**^**Ph**^]_*t*=0_ = 0.0118 M, [py] = 0, [acrylate] = 0.177 M, solvent: C_6_D_6_, *V* (total) = 0.5 mL, *T* = 25 °C. (h) Plot of the reverse of first-order rate constants of β-H elimination [1/*k* (step 2), or 1/*k*_2_] vs [py]/[Ni_*t*=0_] for **1**^**Ph**^. Condition for h): [Ni] = 0.0118 M, [py] = 0039–0.059 M, [MA] = 0.59 M, solvent: C_6_D_6_, *V* (total) = 0.5 mL, *T* = 25 °C. See Supporting Information Section S7 and S8 for details.

With these two highly active and thermally robust catalysts, tBA insertion and subsequent reactions were investigated. Treatment of **1**^**Ph**^ with excess tBA (ca. 15 equiv) results in a color change from yellow to red. Monitoring of the ^1^H and ^31^P{^1^H} NMR spectra confirmed the consumption of **1**^**Ph**^. One broad resonance appears in ^31^P{^1^H} NMR spectra over time (Figures S14 and S17), and four new resonances were observed in the ^1^H NMR spectra in a ∼1:9:9:9 ratio (Figures S15, S16 and S18): one new doublet in the olefinic region (δ ∼ 5.8 ppm), one in the upfield region corresponding to a Me_3_Si-containing species (δ ∼ 0 ppm), and two ^*t*^BuO- resonances (δ 1.2–1.5 ppm). These results suggest reactivity with two acrylates and the generation of a new olefinic species. A combination of ^1^H–^1^H COSY NMR and gas chromatography–mass spectrometry analysis revealed the identity of the internal olefin as **^^*t*^**Bu**^IO^Si^** (Figure 3a, Figures S18–S22). Furthermore, ^1^H, ^31^P{^1^H}, and ^1^H–^1^H COSY NMR analysis suggests the identity of the other species as **4**^**Ph-**^***t***^**Bu**^, which is most likely generated via tBA insertion into a Ni hydride complex (**3**^**Ph**^, Figures 3a, S23 and S24).

### Structural Characterization of **4^Ph-^*t*^Bu^**

Despite numerous attempts, bulk isolation of pure **4**^**Ph-**^***t***^**Bu**^ as a solid was not successful. The complex decomposes quickly at room temperature, both under vacuum and in solution. Nevertheless, single crystals of **4^Ph-^*t*^Bu^** were obtained from tBA insertion experiments with **1^Ph^** in the presence of tBA and excess pyridine (ca. five equiv), and the scXRD structure of **4**^**Ph-**^***t***^**Bu**^ is shown in Figure 3b. To the best of our knowledge, this is the first crystallographic characterization of an intermediate generated after polar-monomer-induced β-H elimination relevant to Ni-catalyzed polar polyolefin synthesis. The Ni(1)–C(1) distance in **4**^**Ph-**^***t***^**Bu**^ [2.030(5) Å] is longer than that in **1**^**Ph**^ [1.949(2) Å] or in reported Ni complexes resulting from tBA insertion into a metal alkyl moiety [1.972(8) ∼ 2.003(8) Å].^37,49^ This comparison suggests a weakened Ni–alkyl bond in **4**^**Ph-**^***t***^**Bu**^, potentially due to steric repulsion induced by the bulky phenoxy and ^*t*^Bu groups. These steric interactions may also promote facile β-H elimination in crowded intermediate **2**^**Ph-**^***t***^**Bu**^.

### Kinetic Studies of Acrylate-Induced β-H Elimination

Identification of the internal olefin **^^*t*^**Bu**^IO^Si^** and **4**^**Ph-**^***t***^**Bu**^, in combination with in situ ^1^H and ^31^P{^1^H} NMR monitoring, established a kinetic profile of the reactions with tBA (Figure 3c). The concentration of **4**^**Ph-**^*t*^**Bu**^ is roughly equal to that of **^^*t*^**Bu**^IO^Si^** during the course of the reaction and the two putative intermediates, **2**^**Ph-**^***t***^**Bu**^ and **3**^**Ph**^, were not observed, indicating that acrylate insertion (step 1) is rate determining in this reaction. In contrast, **2**^**Ph-nBu**^ and **2**^**Ph-Me**^ were observed as the intermediates in analogous reactions with ^*n*^butyl acrylate (^*n*^BA) and methyl acrylate (MA), indicating that these two acrylates feature faster rates of initial insertion (step 1) and a lower tendency for β-H elimination after acrylate insertion compared to tBA (Figure 3c–e). Consequently, acrylate insertion (step 1) and β-H elimination (step 2) are differentiable in the kinetic profile (Figure 3d,e), allowing direct quantitative kinetic studies of β-H elimination to elucidate the mechanism. **3**^**Ph**^ was still not observed, suggesting that acrylate reinsertion after β-H elimination (step 3) is faster than β-H elimination (step 2) in these cases.

Next, quantitative kinetic studies of β-H elimination were performed with ^*n*^BA and MA under otherwise identical conditions. After full consumption of **1**^**Ph**^, decay of **2**^**Ph-nBu**^ or **2**^**Ph-Me**^ is representative of β-H elimination (step 2). Notably, linear relationships were observed in the log plot for the decay of relative concentrations of **2**^**Ph-nBu**^ or **2**^**Ph-Me**^ over time (Figure 3f–g), consistent with *pseudo*-first-order kinetics. Comparing ^*n*^BA with MA, β-H elimination induced by the former, bulkier monomer, features a >50% faster rate constant (*k*_2_). This scenario suggests that β-H elimination (step 2) is faster from the insertion product derived from the larger monomer than from the smaller one. In contrast, the initial insertion (step 1) is fastest with MA, the smallest monomer among MA, ^*n*^BA, and tBA, as evidenced by the faster decrease of **1**^**Ph**^ in the reaction with MA compared to those with ^*n*^BA and tBA. Consequently, reaction of **1**^**Ph**^ and tBA, the bulkiest monomer among these three, features the slowest rate of acrylate insertion (step 1) and the fastest rate of subsequent β-H elimination (step 2), leading to **2**^**Ph-**^*t*^**Bu**^ and **3**^**Ph**^ not being observed during the tBA reaction. This generation of **4**^**Ph-**^***t***^**Bu**^ free of intermediates is critical for its isolation.

Furthermore, the rate constant of acrylate-induced β-H elimination (step 2) was measured with **1**^**Ph**^ under varying acrylate and pyridine concentrations (15 or 50 equiv of MA, 0–5 equiv of pyridine) to investigate the influence of acrylate monomer and labile ligand, pyridine. MA instead of BA was selected to ensure better differentiation of acrylate insertion (step 1) and β-H elimination (step 2). A linear relationship was observed between *pseudo*-first-order rate constant of MA insertion (*k*_1_, step 1) and the reverse of pyridine concentration (1/[py]), consistent with the reported mechanism for acrylate insertion.^37^ For acrylate-induced β-H elimination (step 2), a near–linear correlation was observed between the inverse of a *pseudo*-first-order rate constant (1/*k*_2_) and the pyridine concentration ([py], Figure 3e). These observations are consistent with pyridine being involved in the β-H elimination step in this model system, likely through a dissociative process (step 2, Figure 3a; Detailed discussion: Supporting Information section S9). We have previously reported that the labile ligand (i.e., pyridine) also affects the rate of catalyst initiation and chain propagation during polymerization catalysis by neutral Ni catalysts, suggesting that either the resting state of this catalysis is a pyridine-coordinated species or pyridine binding to metal is competitive with olefin coordination.^37,49,77^ Together, these results highlight that the effect of the labile ligand (i.e., pyridine) is a critical consideration in designing neutral Ni catalysts for polar polyolefin synthesis. Notably, *k*_2_ does not depend on [MA] (Table S4, Figures S49 and S50), indicating that acrylate is not involved in this portion of the mechanism. Therefore, a mechanism involving β-H-transfer to olefin is inconsistent.^78–80^

### Effect of the Catalyst Structure on Acrylate-Induced β-H Elimination and Competing Catalyst Deactivation

Effects of the catalyst structure on acrylate-induced β-H elimination and subsequent reactions were investigated by comparing the reaction of tBA with **1**^**Ph**^ and with **1**^**Me**^. Initial tBA insertion for the latter is faster by almost an order of magnitude [0.042(1) min^–1^ vs 0.0061(1) min^–1^, Figures S47 vs S48], while subsequent β-H elimination is slower, as indicated by slower **^^*t*^**Bu**^IO^Si^** generation (Figures 3c vs Figure S44). These observations are consistent with the behavior observed when the size of the monomer was changed—larger steric profiles induce lower rates of insertion but faster β-H elimination.

**Figure 4:**
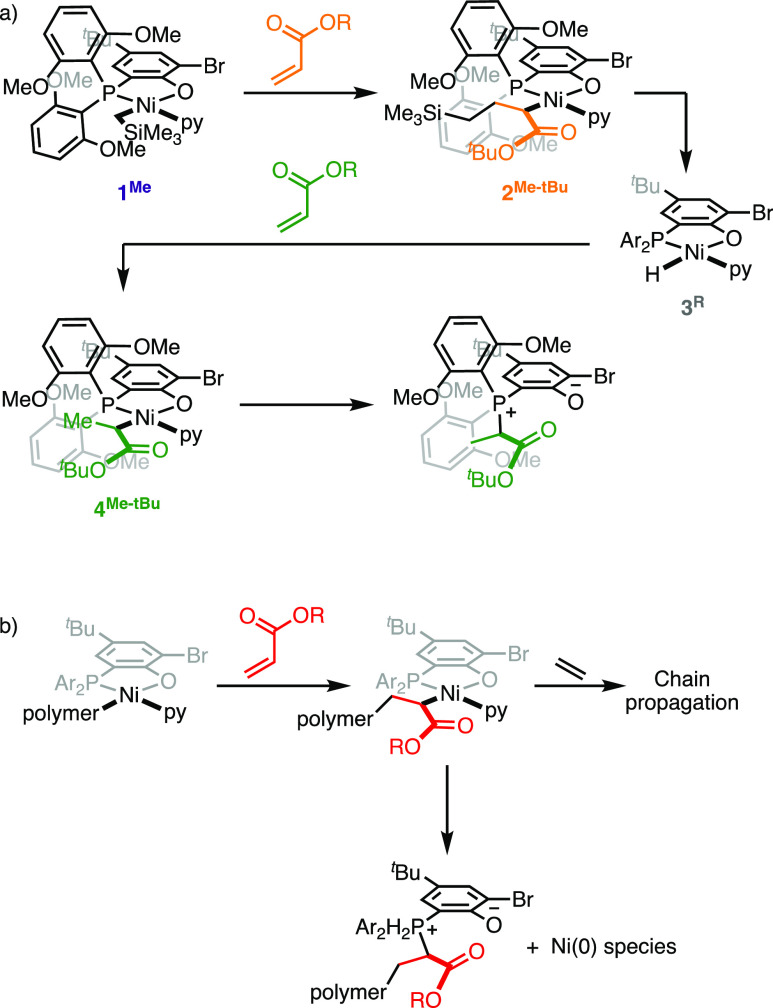
(a) Proposed reaction pathway of **1**^**Me**^ upon treatment with tBA that generates phosphonium species. (b) Corresponding potential catalyst deactivation pathway occurring during catalysis.

Competing with β-H elimination, side reactions that appear to generate phosphonium species were also observed with the smaller ligand (Figures S42 and S43), as indicated by the peak observed at ∼8 ppm in the ^31^P{^1^H} NMR spectra of **1**^**Me**^ upon treatment with tBA. This resonance is close to ^31^P{^1^H} NMR resonance of several phosphonium species reported in the literature.^81^ In addition, matrix-assisted laser desorption ionization–time-of-flight (MALDI–TOF) analysis of the mixture after the reaction of **1**^**Me**^ and tBA indicates the generation of a species with MW = 661 (Figure S45), consistent with the phosphonium species generated by reductive elimination of **4**^**Me-**^***t***^**Bu**^ along with alkyl transferring to the phosphine (Figures 4a and Figure S46). This process is potentially related to catalyst deactivation pathways during the copolymerization reaction (Figure 4b).^66,81^ Indeed, the ethylene uptake curve of ethylene/acrylate copolymerization by **1**^**Me**^ at 110 °C reveals that ethylene consumption slows quickly (Figure S6), indicating fast catalyst decomposition during copolymerization. Notably, the phosphonium species was not observed in the reactions of acrylate with **1**^**Ph**^. Overall, these results suggest that the larger axial shielding in **1**^**Ph**^ compared to that in **1**^**Me**^ is crucial for stabilizing intermediates generated in acrylate-induced reactions and disfavoring undesired side reactions, as suggested by the higher activity (Table 1). However, this comes with a disadvantage of a slower reaction with acrylate and, therefore, decreased incorporation into the copolymer.

### Correlation of Catalyst Behavior in β-H Elimination and in Copolymerization

Correlations between the mechanistic details of acrylate-induced β-H elimination at Ni complexes and nickel-catalyzed ethylene/acrylate copolymerization reactions were further examined. Notably, analysis of ethylene/tBA copolymers shows that although **1**^**Ph**^ produces copolymers with lower acrylate incorporation than **1**^**Me**^, **1**^**Ph**^ features significantly higher TOF_tBA_ (moles of tBA inserted per mole of catalyst per hour), compared to **1**^**Me**^ under otherwise identical conditions (Table 2, by ∼3 fold at 90 °C or ∼10-fold at 110 °C). Given that the resting state of this catalysis is the intermediate generated after acrylate insertion and that back to back tBA insertion is very slow,^37^ the higher TOF_tBA_ implies that subsequent ethylene insertion and/or β-H elimination (Figure 1) is faster with **1**^**Ph**^ compared to **1**^**Me**^. Additional analysis of polymer microstructures may provide insights into the competition between β-H elimination and ethylene insertion after tBA insertion (Table S3).

**Table 2:** TOF_E_ and TOF_tBA_ in Ethylene/Acrylate Copolymerization (TOF: Moles of Monomer Inserted per Mole of Catalyst per Hour)

entry^a^	catalyst	*T*/°C	[tBA]/M	TOF_E_	TOF_tBA_
1	**1**^**Me**^	90	0.05	51,000	800
2	**1**^**Ph**^	90	0.05	740,000	2500
3	**1**^**Me**^	90	0.10	22,000	750
4	**1**^**Ph**^	90	0.10	340,000	2500
5	**1**^**Me**^	90	0.15	12,000	610
6	**1**^**Ph**^	90	0.15	200,000	1900
7	**1**^**Me**^	110	0.10	14,000	420
8	**1**^**Ph**^	110	0.10	620,000	4400

a Each entry represents average of multiple replicated runs. For polymerization conditions and other catalytic data for entries 1–8, see Table 1 entries 2, 6, 3, 7, 4, 8, 5, and 9, respectively.

**Figure 5:**
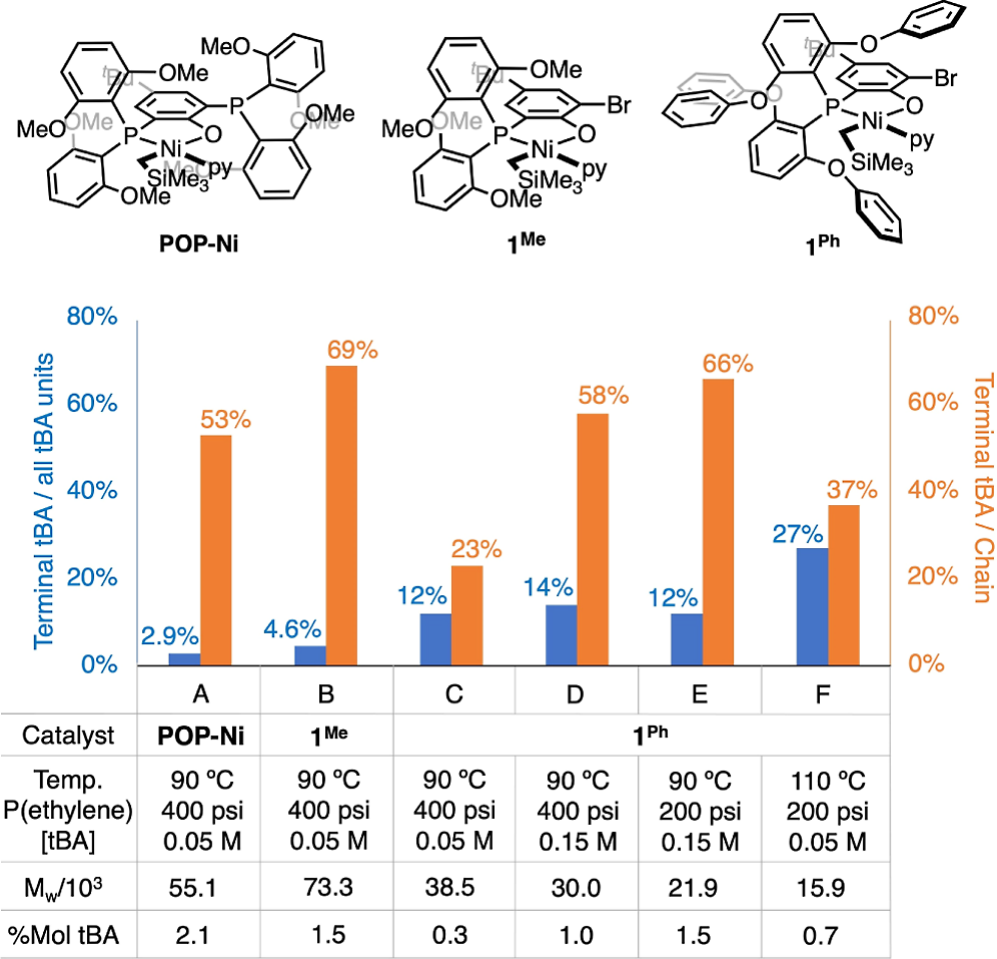
Structural analysis of ethylene/acrylate copolymers (see Tables S1 and S3 for catalysis data and other details).

β-H elimination after acrylate insertion results in ester chain-ends, while competing ethylene insertion results in in-chain acrylate units. In copolymers produced by **POP-Ni**, a catalyst we reported earlier,^37^**1**^**Me**^, and **1**^**Ph**^, approximately 23–69% of unsaturated chain-ends are ester chain-ends despite acrylate content being below 3 mol % in these samples, confirming that acrylate-induced β-H elimination is an important pathway for chain-termination (Figure 5).^82^ Because the majority of acrylate units are located in-chain (approximately 73–97%), ethylene insertion rather than β-H elimination remains the major event occurring after acrylate insertion. In this regard, the aforementioned higher TOF_tBA_ of **1**^**Ph**^ compared to **1**^**Me**^ suggests that ethylene insertion after tBA insertion is faster with **1**^**Ph**^ than that with **1**^**Me**^; therefore, a larger ligand lowers the residence time of the acrylate-inserted species, the resting state of catalysis. Changes in the tBA concentration during copolymerizations with **1**^**Ph**^ show minimal effects on the terminal tBA/total tBA ratio in the resulting copolymers even though the total tBA content is affected. This finding is related to the impact on polymer *M*_w_ which decreases with the increased tBA concentration, as expected for chain termination promoted upon tBA insertion.^82^ Notably, doubling in terminal tBA/all tBA units (C vs F) is feasible by tuning the reaction temperature and ethylene pressure, while tripling in terminal tBA/chain (C vs E) is achievable by tuning the acrylate concentration and/or ethylene pressure (Figure 5). These results demonstrate potential strategies to control acrylate-induced β-H elimination and the polymer microstructure. Changing the catalyst structure further expands the range of tBA incorporation profiles.

## Summary

In summary, **1**^**Me**^ and **1**^**Ph**^ are high-performance catalysts for ethylene/acrylate copolymerization. Specifically, **1**^**Ph**^ shows activity >30,000 kg/(mol·h) for 0.3 mol % tBA incorporation at 110 and 130 °C, demonstrating a new level of activity and thermostability. Still, further improvements in acrylate incorporation are needed for an alternative to the industrial radical process. On the other hand, such copolymers with low acrylate incorporation could potentially serve as telechelic monomers for preparation of chemically recyclable ester linked polyolefins.^83–85^ This catalyst system is of potential interest for this purpose, given its high activity.

Additionally, this ligand class allowed for investigations of polar monomer (acrylate)-induced β-H elimination, an underexplored elementary step in polar polyolefin synthesis. An intermediate, **4**^**Ph-**^***t***^**Bu**^, generated from a putative Ni-hydride, was characterized by crystallography, confirming alkyl chain release by acrylate-induced β-H elimination. Tuning the steric profiles of acrylate monomers leads to significant changes in the rates of acrylate insertion and subsequent β-H elimination, allowing differentiation of these two steps in the kinetic profile and direct, quantitative kinetic studies of β-H elimination.

Analysis of ethylene/acrylate copolymers correlates with kinetic data and shows that β-H elimination after acrylate insertion can be the primary chain termination event even at relatively low tBA incorporation levels of 1%. Competing with β-H elimination, a decomposition pathway from acrylate-inserted compounds that generates phosphonium species is supported by ^31^P{^1^H} NMR and MALDI–TOF analyses. Increased ligand sterics promote acrylate-induced β-H elimination while also being essential for preventing catalytic intermediates from decomposition and thus affording efficient catalysis. Overall, a combination of mechanistic and catalysis studies demonstrates the role of acrylate-induced β-H elimination as a chain-termination mechanism in copolymerization and its potential in controlling polymer microstructures, providing insights for future studies targeting catalyst developments and polymer synthesis.

## Supporting Information Available

Experimental section, synthetic procedures, characterization data, and polymerization studies (PDF)

## Accession Codes

CCDC 2280179–81 contain the supplementary crystallographic data for this paper. These data can be obtained free of charge via www.ccdc.cam.ac.uk/data_request/cif, or by emailing data_request@ccdc.cam.ac.uk, or by contacting The Cambridge Crystallographic Data Centre, 12 Union Road, Cambridge CB2 1EZ, UK; fax: +44 1223 336033.
