# Supplementary material for: Acrylate-Induced β-H Elimination in Coordination Insertion Copolymerizaton Catalyzed by Nickel
Source: J Am Chem Soc. 2023 Nov 22;145(48):26463–71. doi: 10.1021/jacs.3c10800 (PMC10704591; doi:10.1021/jacs.3c10800)
Supplement: Supplementary file 1 — ja3c10800_si_001.pdf [file ja3c10800_si_001.pdf]

Supporting Information for

Acrylate-Induced β-H Elimination in Coordination Insertion Copolymerization Catalyzed by Nickel

Shuoyan Xiong[a], Alexandria Hong[a], Priyabrata Ghana[a], Brad C. Bailey[b], Heather A. Spinney[b], Hannah Bailey[b], Briana S. Henderson[b], Steve Marshall[b], Theodor Agapie*[a]

* To whom correspondence should be addressed, E-mail: agapie@caltech.edu

[a] *Division of Chemistry and Chemical Engineering, California Institute of Technology, Pasadena, California 91125, United States.*
[b] *Chemical Science, Core R&D, The Dow Chemical Company, Midland, Michigan 48667.*

1. Procedures for synthesis of ligands and metal complexes S2

2. Topographical analyses S5

3. Procedures for polymerization and polymer characterization S7

4. Supplemental data for ethylene/tBA copolymerization S11

5. Characterization of ethylene/tBA copolymers S16

6. Catalyst comparison S19

7. Investigations of acrylate-induced reactions S22

8. Quantitative kinetic studies of acrylate-induced reactions S47

9. Discussion of pathways of β-H elimination S56

10. Crystallographic information S59

11. NMR spectra of ligands and organometallic complexes S63

References S68

1. Procedures for Synthesis of Ligands and Metal Complexes

1.1. General Considerations

All air- and water-sensitive compounds were manipulated under N_2 or Ar using standard Schlenk or glovebox techniques. The solvents for air- and moisture-sensitive reactions were dried over sodium benzophenone/ketyl, calcium hydride, or by the method of Grubbs.[1] Deuterated solvents were purchased from Cambridge Isotopes Lab, Inc.; C_6D_6, was dried over a purple suspension with Na/benzophenone ketyl and vacuum transferred. t-Butyl acrylate was dried over 4 Å sieves for greater than 72h. 2.5 M ^{n}BuLi in hexanes were purchased from Sigma-Aldrich and used without further purification. 1,3-Dibromo-5-(tert-butyl)-2-(methoxymethoxy)benzene,[2] bis(dimethoxyphenyl)phosphine chloride,[3] bis(diphenoxyphenyl)phosphine chloride,[4] $py_2Ni(CH_2SiMe_3)_2$[5] and 2-bromo-4-tertbutyl-5-bis(dimethoxyphenyl)phosphino)phenol[6] were synthesized according to literature procedures. All ^{1}H, ^{13}C, and ^{31}P spectra of organic and organometallic compounds were recorded on Varian INOVA-400, or Bruker Cryoprobe 400 spectrometers. ^{1}H and ^{13}C chemical shifts are reported relative to residual solvent resonances.

1.2. Synthesis of Ligands and Metal Complexes

Ligand ***^{PhO}POBrH:*** A Schlenk flask fitted with a screw-in Teflon stopper was charged with a solution of 1,3-dibromo-5-(tert-butyl)-2-(methoxymethoxy)benzene (3.52 g, 10.0 mmol) in THF (40 mL) and cooled to -78 °C under nitrogen. A hexane solution of *n*-butyllithium (4 mL, 2.5 M, 10.0 mmol) was added dropwise via syringe. After stirring for an additional 30 min at -78 °C, a solution of bis(2,6-diphenoxyphenyl)phosphine chloride (5.89g, 10.0 mmol) in THF (20 mL) was added dropwise via cannula. After complete addition, the reaction was allowed to warm up to room temperature and stirred for an additional 3 h, yielding a yellow solution. The solution was then concentrated to ~20 mL and degassed MeOH (10 mL) and concentrated aqueous HCl (10 mL, degassed by three freeze-pump-thaw cycles with a liquid nitrogen bath prior to usage) were added. After stirring for 12 h under room temperature, volatiles were removed under vacuum. In a N_2-filled glovebox (no exclusion of water), the resulting yellow residue was taken up in CH_2Cl_2 (20 mL), washed with saturated aqueous solutions of K_2CO_3 (3 x

10 mL) and NH_4Cl (3 x 10 mL), dried over $MgSO_4$, and filtered through Celite. The volatiles were removed under reduced pressure. In a glovebox (exclusion of water and oxygen), the resulting pale-yellow solid was was washed by cold pentane (3 x 20 mL), then dissolved in Et_2O and filtered through Celite. The volatile materials were removed once more under vacuum, yielding **^{PhO}POBrH** (3.82g, 95% purity) as gel-like solids. This material was then used in metalation as the proligand without further purification. ^{1}H NMR (400 MHz, C_6D_6, *note: only resonances assigned to protons of the desired product were listed*): δ 7.69 (dd, *J* = 9.7, 2.3 Hz, 1H, ArH), 7.33 (d, *J* = 2.3 Hz, 1H, ArH), 6.98 – 6.92 (m, 8H, ArH), 6.85 – 6.75 (m, 12H, ArH), 6.72 – 6.64 (m, 3H, ArH + ArOH), 6.44 (dd, *J* = 8.2, 2.8 Hz, 4H, ArH), 0.99 (s, 9H, -Si(CH_3)$_3$); ^{31}P{^{1}H} NMR (162 MHz, C_6D_6): δ -51.70 (s).

*Complex **1^{Ph}**:* In the glove box, to a solution of $Py_2Ni(CH_2SiMe_3)_2$ (44 mg, 0.119 mmol) in benzene (4 ml) in a vial was added a solution of **^{PhO}POBrH** (92.9 mg, 0.119 mmol) in benzene (8 ml). The mixture was stirred for 2 h under room temperature, forming a red-brown solution. Volatile materials were removed under vacuum. The residue was extracted with pentane (3 x 5 mL), then washed by cold pentane (3 x 15 mL), pentane (3 x 5 mL) and hexanes (3 x 2 mL), The solid was collected via vacuum filtration, and redissolved in Et_2O, filtered through Celite. The volatile materials were removed once more under vacuum, yielding spectroscopically pure **1^{Ph}** (50 mg, 45%) as yellow-orange solids. ^{1}H NMR (400 MHz, C_6D_6): δ 8.76 (dd, *J* = 4.9, 1.6 Hz, 2H, ArH), 7.71 – 7.65 (m, 1H, ArH), 7.51 (d, *J* = 2.3 Hz, 1H, ArH), 7.12 – 7.07 (m, 8H, ArH), 7.06 – 6.99 (m, 8H, ArH), 6.88 – 6.79 (m, 5H, ArH), 6.77 – 6.70 (m, 2H, ArH), 6.56 – 6.48 (m, 5H, ArH), 0.90 (s, 9H, -^{t}Bu), -0.00 (s, 9H, -$SiMe_3$), -0.61 (d, *J* = 9.8 Hz, 2H, $NiCH_2Si$). ^{13}C{^{1}H} NMR (101 MHz, C_6D_6): δ 168.74 (d, *J* = 25.2 Hz, 1C, ArC), 160.01 (s, 4C, ArC), 155.86 (s, 4C, ArC), 151.68 (s, 2C, ArC), 136.44 (s, 1C, ArC), 135.97 (d, *J* = 7.6 Hz, 1C, ArC), 132.80 (s, 1C, ArC), 130.45 (s, 2C, ArC), 129.92 (s, 8C, ArC), 128.59 (s, 1C, ArC), 126.45 (d, *J* = 2.8 Hz, 1C, ArC), 124.06 (s, 4C, ArC), 123.27 (s, 2C, ArC), 123.20 (d, *J* = 52.4 Hz, 1C, ArC), 120.56 (s, 8C, ArC), 114.06 (d, *J* = 44.4 Hz, 2C, ArC), 110.72 (d, *J* = 4.0 Hz, 4C, ArC), 33.73 (s, 1C, -$\underline{C}(CH_3)_3$), 31.65 (s, 3C, -$C(\underline{C}H_3)_3$), 2.64 (s, 3C, $SiMe_3$), -16.11 (d, *J* = 27.2 Hz, 1C, $NiCH_2Si$); ^{31}P{^{1}H} NMR (121 MHz, C_6D_6): δ -2.49 (s, 1P). Anal. Calcd(%) for $C_{55}H_{53}BrNNiO_5PSi$: C, 65.69; H, 5.31; N, 1.39. Found(%): C, 66.12; H, 5.40; N, 1.11.

*Complex **1^{Me}***: In the glove box, to a solution of $Py_2Ni(CH_2SiMe_3)_2$ (44 mg, 0.119 mmol) in benzene (4 ml) in a vial was added a solution of **^{MeO}POBrH** (63.3mg, 0.119 mmol) in benzene (8 ml). The mixture was stirred for 2

h under room temperature, forming a red-brown solution. Volatile materials were removed under vacuum. The residue was extracted with pentane (3 x 5 mL), then washed by pentane (3 x 10 mL), hexanes (3 x 5 mL) and Et_2O (2 x 2 mL). The solid was collected via vacuum filtration, and redissolved in benzene, filtered through Celite. The volatile materials were removed once more under vacuum, yielding **1^{Me}** (50 mg, 45%) as brown solids. ^1H NMR (400 MHz, C_6D_6): δ 9.19 (d, *J* = 6.5 Hz, 2H, ArH), 7.68 (d, *J* = 2.3 Hz, 1H, ArH), 7.62 (dd, *J* = 11.1, 2.4 Hz, 1H, ArH), 7.10 (t, *J* = 8.3 Hz, 2H, ArH), 6.93 – 6.81 (m, 1H, ArH), 6.60 (t, *J* = 7.3 Hz, 2H, ArH), 6.28 (dd, *J* = 8.3, 3.7 Hz, 4H, ArH), 3.27 (s, 12H, $-OCH_3$), 1.13 (s, 9H, -*t*Bu), -0.13 (s, 9H, $-SiMe_3$), -0.59 (d, *J* = 9.2 Hz, 2H, $NiCH_2Si$). $^{13}C\{^1H\}$ NMR (101 MHz, C_6D_6): δ 168.17 (d, *J* = 24.5 Hz, 2C, ArC), 161.73 (s, 4C, ArC), 151.60 (s, 2C, ArC), 136.61 (s, 1C, ArC), 135.60 (d, *J* = 7.4 Hz, 1C, ArC), 131.52 (s, 1C, ArC), 130.81 (s, 2C, ArC), 128.60 (s, 1C, ArC), 126.33 (d, *J* = 2.8 Hz, 1C, ArC), 123.75 (s, 2C, ArC), 112.74 (d, *J* = 15.2 Hz, 1C, ArC), 110.85 (d, *J* = 48.5 Hz, 1C, ArC), 104.69 (d, *J* = 4.6 Hz, 4C, ArC), 55.51 (s, 4C, -OMe), 33.81 (s, 1C, $-\underline{C}(CH_3)_3$), 32.02 (s, 3C, $-C(\underline{C}H_3)_3$), 2.30 (s, 3C, $SiMe_3$), -17.74 (d, *J* = 30.0 Hz, 1C, $NiCH_2Si$); $^{31}P\{^1H\}$ NMR (121 MHz, C_6D_6): δ -5.08 (s, 1P). Anal. Calcd(%) for $C_{35}H_{45}BrNNiO_5PSi$: C, 55.50; H, 5.99; N, 1.85. Found(%): C, 55.02; H, 5.77; N, 1.72.

2. Topographical Analyses

Results

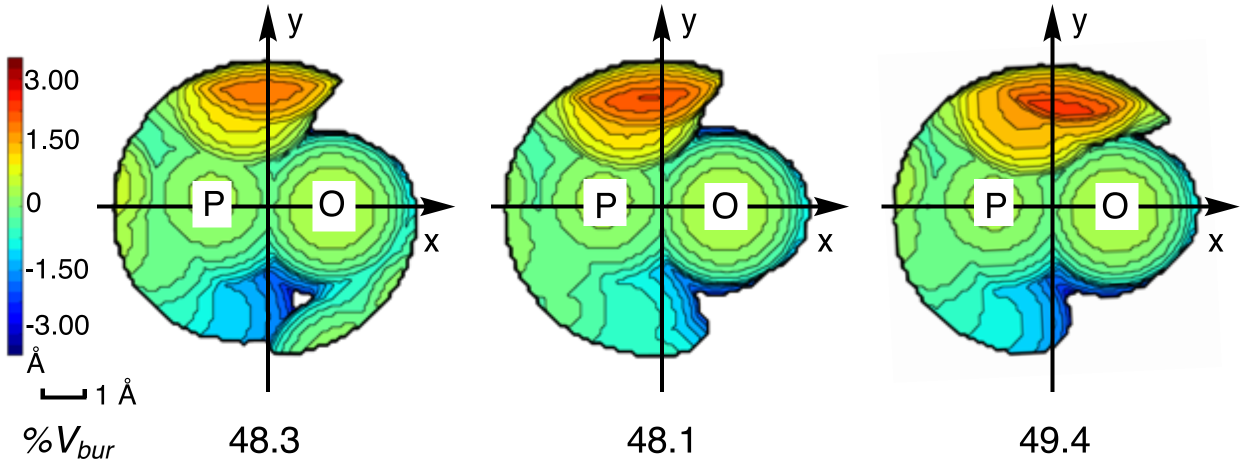

Figure S1. Topographical steric maps with %V_{bur} of **POP-Ni** (left), $\mathbf{1^{Me}}$ (middle), and $\mathbf{1^{Ph}}$ (right). The Ni atom defines the origin of xyz coordinate system. Only the P,O-ligand included in calculation and steric visualization. Blue indicates occupied space in the -z direction (toward back as drawn in a), where the phosphine-phenoxide ligands are located, and red indicates +z direction. See section S3 for more details.

Details for topographical analyses. Topographical maps of **POP-Ni**, $\mathbf{1^{Me}}$, and $\mathbf{1^{Ph}}$ and corresponding percent buried volume data (%V_{bur}) were generated by Cavallo's SambVca 2.1 (Salerno molecular buried volume calculation) program.[7-10]

More details for %V_{bur} calculation and steric maps:

1) The nickel atom (Ni1) defines the center of the xyz coordinate system,

2) <u>$Ni(PEt_3)Ph$ fragment was excluded</u>;

3) Bondi radii was scaled by 1.17; [9]

4) Mesh spacing for numerical integration was 0.10;

5) Sphere radius was set to 3.5 Å;

6) H atoms were excluded.

7) xz-Plane was defined as shown in the figure below and the y -axis was defined by the right-hand rule (for **POP-Ni**) or the reverse (for the other two). This is to ensure the larger axial shielding locates on the top for easier comparison. Note that for a specific complex, the %Vbur remained the same with Ni1 in the origin even the xyz coordination system rotated or flipped.

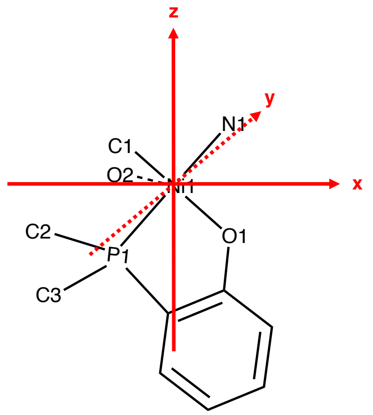

3. Procedures for Polymerization and Polymer Characterization

3.1. General procedure for high throughput parallel polymerization reactor (PPR) runs.

Polyolefin catalysis screening was performed in a high throughput parallel polymerization reactor (PPR) system. The PPR system was comprised of an array of 48 single cell (6 x 8 matrix) reactors in an inert atmosphere glovebox. Each cell was equipped with a glass insert with an internal working liquid volume of approximately 5 mL. Each cell had independent controls for pressure and was continuously stirred at 500 rpm. Catalyst solutions were prepared in toluene. All liquids (i.e., solvent, tBA, and catalyst solutions) were added via robotic syringes. Gaseous reagents (i.e., ethylene) were added via a gas injection port. Prior to each run, the reactors were heated to 50 °C, purged with ethylene, and vented.

All desired cells were injected with a solution of tBA in toluene followed with a portion of toluene (This step was skipped for ethylene homopolymerization). Note: tBA was purified by passaging a plug of activated alumina prior to use. The reactors were heated to the run temperature and then pressured to the appropriate psig with ethylene. Catalyst solutions were then added to the cells. Each catalyst addition was chased with a small amount of toluene so that after the final addition, a total reaction volume of 5 mL was reached. Upon addition of the catalyst, the PPR software began monitoring the pressure of each cell. The desired pressure (within approximately 2-6 psig) was maintained by the supplemental addition of ethylene gas by opening the valve at the set point minus 1 psi and closing it when the pressure reached 2 psi higher. All drops in pressure were cumulatively recorded as "Uptake" or "Conversion" of the ethylene for the duration of the run or until the uptake or conversion requested value was reached, whichever occurred first. Each reaction was then quenched by addition of 1% oxygen in nitrogen for 30 seconds at 40 psi higher than the reactor pressure. The pressure of each cell was monitored during and after the quench to ensure that no further ethylene consumption happens. The shorter the "Quench Time" (the duration between catalyst addition and oxygen quench), the more active the catalyst. In order to prevent the formation of too much polymer in any given cell, the reaction was quenched upon reaching a predetermined uptake level of 80 psig for copolymerization reactions (60 psig for ethylene homopolymerization). After all the reactors were quenched, they were allowed to cool to about 60

°C. They were then vented, and the tubes were removed. The polymer samples were then dried in a centrifugal evaporator at 60 °C for 12 hours, weighed to determine polymer yield and used in subsequent IR (tBA incorporation), GPC, DSC and NMR (copolymer microstructures) analysis.

3.2. General procedure for batch reactor runs for preparation of ethylene/tBA copolymers.

Polymerization reactions were conducted in a 2-L Parr batch reactor. The reactor was heated by an electrical heating mantle and cooled by an internal serpentine cooling coil containing cooling water. The water was pre-treated by passing through an Evoqua water purification system. Both the reactor and the heating/cooling system were controlled and monitored by a LabVIEW process computer. The bottom of the reactor was fitted with a dump valve, which empties the reactor contents into a lidded dump pot, which was prefilled with a catalyst-kill solution (typically 5 mL of an Irgafos / Irganox / toluene mixture). The lidded dump pot was vented to a 15-gal. blowdown tank, with both the pot and the tank N_2 purged. All chemicals used for polymerization or catalyst makeup are run through purification columns to remove any impurities that may affect polymerization. The toluene was passed through two columns, the first containing A2 alumina, the second containing Q5 reactant. The tert-butyl acrylate was filtered through activated alumina. The ethylene was passed through two columns, the first containing A204 alumina and 4 Å molecular sieves, the second containing Q5 reactant. The N2 used for transfers was passed through a single column containing A204 alumina, 4 Å molecular sieves and Q5 reactant.

The reactor was loaded first from the shot tank that contained toluene and tBA. The shot tank was filled to the load set points by use of a differential pressure transducer. After solvent/acrylate addition, the shot tank was rinsed twice with toluene. Then the reactor was heated up to the polymerization temperature set point. The ethylene was added to the reactor when the reaction temperature was reached to maintain the reaction pressure set point. Ethylene addition amounts were monitored by a micro-motion flowmeter.

The catalysts were handled in an inert atmosphere glovebox and were prepared as a solution in toluene. The catalyst was drawn into a syringe and pressure-transferred into the catalyst shot tank. This was followed by 3 rinses of toluene, 5 mL each. Catalyst was added when the reactor pressure set point was reached.

Immediately after catalyst addition the run timer was started. Ethylene was then added by the LabVIEW controller to maintain reaction pressure set point in the reactor. These polymerizations were run for 60 min or until 40 g of ethylene uptake. Then the agitator was stopped, and the bottom dump valve was opened to empty reactor contents into the lidded dump pot. The lidded dump pot was closed, and the contents were poured into trays placed in a lab hood where the solvent was evaporated off overnight. The trays containing the remaining polymer were then transferred to a vacuum oven, where they were heated up to 140 °C under vacuum to remove any remaining solvent. After the trays cooled to ambient temperature, the polymers were weighed for yield/efficiencies and submitted for polymer testing if so desired.

3.3. Procedure for gel permeation chromatography (GPC).

High temperature GPC analysis was performed using a Dow Robot Assisted Delivery (RAD) system equipped with a Polymer Char infrared detector (IR5) and Agilent PLgel Mixed A columns. Decane (10 μL) was added to each sample for use as an internal flow marker. Samples were first diluted in 1,2,4-trichlorobenzene (TCB) stabilized with 300 ppm butylated hydroxyl toluene (BHT) at a concentration of 10 mg/mL and dissolved by stirring at 160°C for 120 minutes. Prior to injection the samples are further diluted with TCB stabilized with BHT to a concentration of 3 mg/mL. Samples (250 μL) are eluted through one PL-gel 20 μm (50 x 7.5 mm) guard column followed by two PL-gel 20 μm (300 x 7.5 mm) Mixed-A columns maintained at 160 °C with TCB stabilized with BHT at a flowrate of 1.0 mL/min. The total run time was 24 minutes. To calibrate for molecular weight (MW) Agilent EasiCal polystyrene standards (PS-1 and PS-2) were diluted with 1.5 mL TCB stabilized with BHT and dissolved by stirring at 160 °C for 15 minutes. These standards are analyzed to create a 3rd order MW calibration curve. Molecular weight units are converted from polystyrene (PS) to polyethylene (PE) using a daily Q-factor calculated to be around 0.4 using the average of 5 Dowlex 2045 reference samples.

3.4. Procedure for fourier-transform infrared spectroscopy (FTIR).

The 10 mg/mL samples prepared for GPC analysis are also utilized to quantify tert-butyl acrylate (tBA) incorporation by Fourier Transform infrared spectroscopy (FTIR). A Dow robotic preparation station heated

and stirred the samples at 160°C for 60 minutes then deposited 130 μL portions into stainless wells promoted on a silicon wafer. The TCB was evaporated off at 160°C under nitrogen purge. IR spectra were collected using a Nexus 6700 FT-IR equipped with a DTGS KBr detector from 4000-400 cm-1 utilizing 128 scans with a resolution of 4. Ratio of tBA (C=O: 1762-1704 cm−1) to ethylene (CH2: 736-709 cm−1) peak areas were calculated and fit to a linear calibration curve to determine total tBA.

3.5. Differential scanning calorimetry (DSC).

Differential scanning calorimetry analyses was performed on solid polymer samples using a TA Instruments, Inc. Discovery Series or TA Instruments, Inc., DSC2500, programmed with the following method: Equilibrate at 175.00 °C; Isothermal for 3 minutes; Ramp 30.00 °C/min to 0.00 °C; Ramp 10.00 °C/min to 175.00 °C; Data was analyzed using TA Trios software.

3.6. NMR characterization.

NMR spectra of ethylene/tBA copolymers were recorded on a Bruker 400 MHz using o-dichlorobenzene at 120 °C. ^{1}H NMR analysis of copolymers were done using a relaxation time (0.2 s), and an acquisition time (1.8 s) with the number of FID's collected per sample (512). ^{13}C{^{1}H} NMR analysis of copolymers were done using 90° pulse of 17.2 μs, a relaxation time (22.0 s), an acquisition time (5.3 s), and inverse-gated decoupling with the number of FID's collected per sample (1536). Analysis of the spectra was based on literature.[11-12]

4. Supplemental Data for Ethylene/tBA Copolymerization

4.1. Analysis of turnover frequency of acrylate (TOF_{tBA})

As shown in Table 2, **1^{Ph}** produces copolymers with lower acrylate incorporation compared to **1^{Me}**. However, the former actually features a significantly higher turnover frequency of acrylate (TOF_{tBA}) compared to the latter under otherwise identical conditions (e.g. entry 2 vs 6, or 3 vs 7, or 8 vs 9). It's also notable that the temperature shows significant impact on TOF_{tBA} while the impact of tBA concentration is moderate.

4.2. Supplemental ethylene /acrylate copolymerization results.

Table S1. Catalysis results for Figure 5 and Table S3.

Entry[a]	catalyst	T/ºC	E/psi	A	A/M	Act.[b]	Mw[c]	Đ	%Mol A	Tm (ºC)
1[d]	**POP-Ni**	90	400	tBA	0.05	660	55.1	2.2	2.1	111
2	**1^{Me}**	90	400	tBA	0.05	1550	73.3	2.4	1.5	115
3	**1^{Ph}**	90	400	tBA	0.05	21000	38.5	2.3	0.3	126
4[e]	**1^{Ph}**	90	400	tBA	0.15	5700	30.0	2.3	1.0	120
5	**1^{Ph}**	90	200	tBA	0.15	910	21.9	2.1	1.5	118
6	**1^{Ph}**	110	200	tBA	0.05	8300	15.9	2.4	0.7	123

[a] V = 5 mL, [Catalyst] = 0.05 mM, ethylene pressure = 400 psi, toluene solvent; each entry represents multiple replicated runs (see section S3 for detailed procedure and Table S4 for original data). [b] Activity in kg/(mol·h). [c] kg/mol. [d] Reported in ref 1. [e] Also included in Table 1 as entry 8.

Original polymerization runs for ethylene/tBA copolymerization

Table S2 Original runs of Table 1 and S1.

Entry	catalyst	T/°C	E (psi)	A	[A]/M	time/s	Yield/mg	Act.[c]	Mw[d]	Đ	%Mol A	Tm (°C)
1	**1**^{Me}	70	400	tBA	0.05	1050	55	754	112.2	2.8	2.4	114
2	**1**^{Me}	70	400	tBA	0.05	1390	72	746	127.5	2.5	2.3	114
3	**1**^{Me}	90	400	tBA	0.05	1003	111	1594	72	2.4	1.5	115
4	**1**^{Me}	90	400	tBA	0.05	1133	118	1499	74.6	2.5	1.5	116
5	**1**^{Me}	90	400	tBA	0.1	2057	105	735	46.7	2.2	3.3	106
6	**1**^{Me}	90	400	tBA	0.1	2223	105	680	47.3	2.2	3.4	106
7	**1**^{Me}	90	400	tBA	0.15	3601	104	416	35.5	2.2	4.9	98
8	**1**^{Me}	90	400	tBA	0.15	3601	103	412	35.2	2.3	4.7	99
9	**1**^{Ph}	90	400	tBA	0.05	116	193	23922	37.9	2.3	0.3	126
10	**1**^{Ph}	90	400	tBA	0.05	129	164	18317	39.1	2.3	0.3	126
11	**1**^{Ph}	90	400	tBA	0.1	232	155	9610	33	2.4	0.7	122
12	**1**^{Ph}	90	400	tBA	0.1	238	162	9816	32.7	2.3	0.8	123
13	**1**^{Ph}	90	400	tBA	0.15	355	141	5717	30.1	2.3	1.0	121
14	**1**^{Ph}	90	400	tBA	0.15	345	137	5714	29.9	2.3	0.9	120
15	**1**^{Me}	110	400	tBA	0.1	3600	119	476	27.7	2.4	3.0	107
16	**1**^{Me}	110	400	tBA	0.1	3600	109	436	25	2.3	3.2	107
17	**1**^{Me}	110	400	tBA	0.1	3601	103	412	25.2	2.4	2.6	107
18	**1**^{Ph}	110	400	tBA	0.1	135	163	17353	18.8	2.5	0.7	123
19	**1**^{Ph}	110	400	tBA	0.1	130	159	17550	18.1	2.4	0.7	122
20	**1**^{Ph}	110	400	tBA	0.1	126	162	18550	16.5	2.5	0.7	123
21	**1**^{Ph}	110	400	tBA	0.15	217	154	10241	6.6	2.5	1.2	120
22	**1**^{Ph}	110	400	tBA	0.15	216	156	10241	7.2	2.5	1.1	120
23	**1**^{Ph}	110	200	tBA	0.05	226	132	8414	15.7	2.8	2.4	114
24	**1**^{Ph}	110	200	tBA	0.05	231	133	8282	16.2	2.5	2.3	114
25	**1**^{Ph}	90	200	tBA	0.15	1471	94	920	21.5	2.1	1.6	116
26	**1**^{Ph}	90	200	tBA	0.15	1547	98	912	21.7	2.1	1.5	117
27	**1**^{Ph}	90	200	tBA	0.15	1841	115	899	22.4	2.2	1.5	121
28	**1**^{Me}	90	400	tBA	0.05	1003	111	1594	72	2.4	1.5	115
29	**1**^{Me}	90	400	tBA	0.05	1133	118	1499	74.6	2.5	1.5	116
30	**1**^{Me}	90	400	tBA	0.15	3601	104	416	35.5	2.2	4.9	98
31	**1**^{Me}	90	400	tBA	0.15	3601	103	412	35.2	2.3	4.7	99
32	**1**^{Ph}	90	400	tBA	0.05	116	193	23922	37.9	2.3	0.3	126
33	**1**^{Ph}	90	400	tBA	0.05	129	164	18317	39.1	2.3	0.3	126
34	**1**^{Ph}	110	400	tBA	0.15	217	154	10241	6.6	2.5	1.2	120
35	**1**^{Ph}	110	400	tBA	0.15	216	156	10241	7.2	2.5	1.1	120

[a] V = 5 mL, [Catalyst] = 0.05 mM, ethylene pressure = 400 psi, toluene solvent; see SI section S3 for detailed procedure. [b] Activity in kg/(mol·h). [c] kg/mol.

4.3. Ethylene uptake curves

Measurement of ethylene uptake curves

During polymerization, the software began monitoring the pressure of the reactor upon addition of the catalyst. The desired pressure (within approximately 2-6 psig) was maintained by the supplemental addition of ethylene gas by opening the valve at the set point minus 1 psi and closing it when the pressure reached 2 psi higher. For example, the pressure was maintained between approximately 399-402 psi if the original pressure was set to 400 psi. All drops in pressure were cumulatively recorded as "Uptake" or "Conversion" of the ethylene for the duration of the run**.** The unit of this "Uptake" is in psi, while the actual amount of ethylene consumed is "ethylene uptake * cell volume". The uptake curves over time were used to analyze the real-time activity of catalysts as ethylene insertion is the primary event for chain propagation given that ethylene unit is the primary component in resulting copolymers.

Representative ethylene uptake curves

1^{Ph}

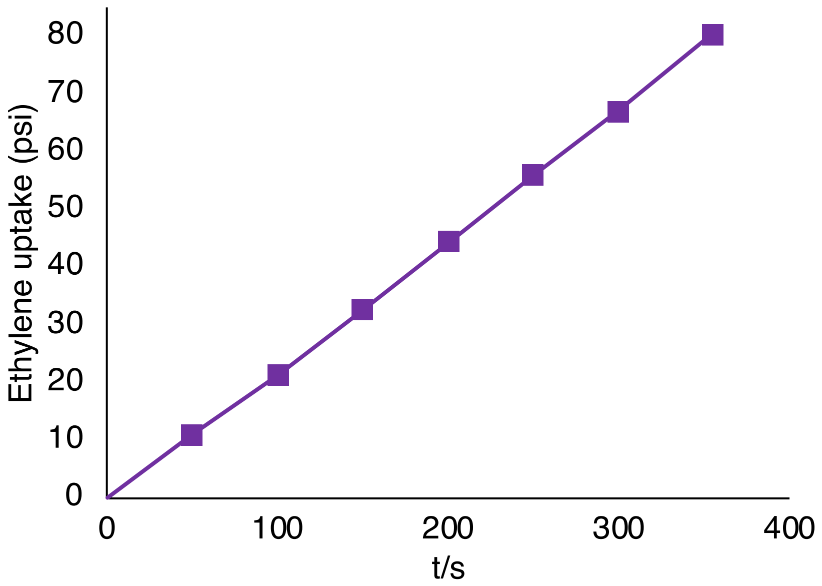

Figure S2. Ethylene uptake curve for **1^{Ph}** of copolymerization under high acrylate concentration. Conditions: V = 5 mL, T = 90 °C, [Ni] = 0.05 mM, ethylene pressure = 400 psi, [tBA] = 0.15 M, toluene solvent. Polymerization was stopped after 1 h. See SI Table S2, entry 13 for other analytical data.

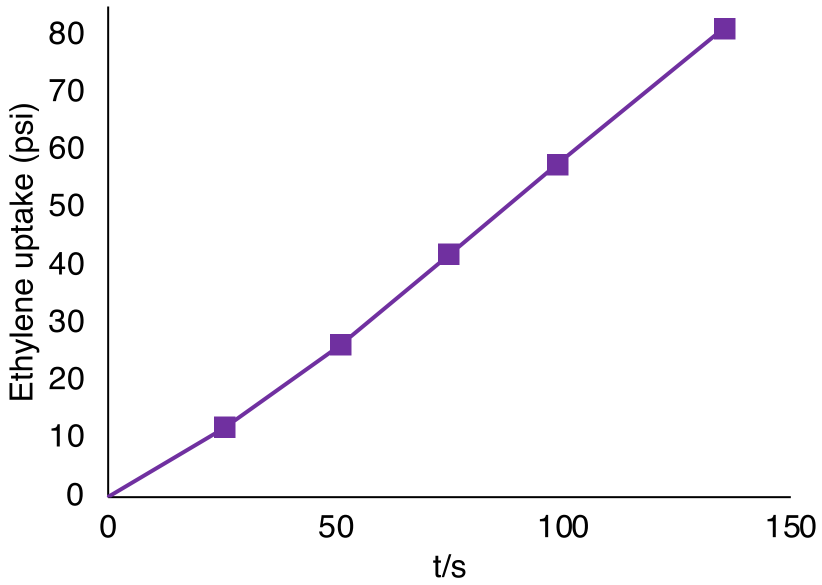

Figure S3. Ethylene uptake curve for **1**^{Ph} of copolymerization at high temperature under moderately high acrylate concentration. Conditions: V = 5 mL, T = 110 °C, [Ni] = 0.05 mM, ethylene pressure = 400 psi, [tBA] = 0.10 M, toluene solvent. Polymerization was stopped after 1 h. See SI Table S2, entry 18 for other analytical data.

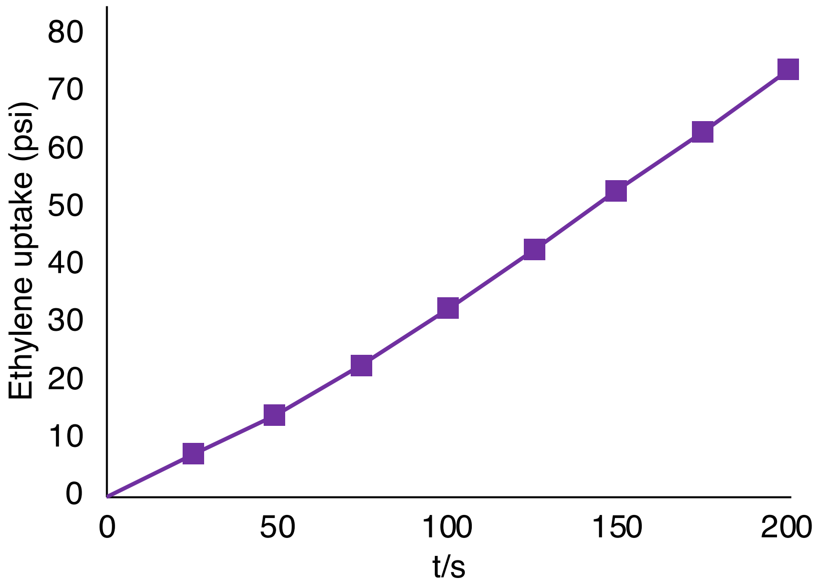

Figure S4. Ethylene uptake curve for **1**^{Ph} of copolymerization at high temperature under high acrylate concentration. Conditions: V = 5 mL, T = 110 °C, [Ni] = 0.05 mM, ethylene pressure = 400 psi, [tBA] = 0.15 M, toluene solvent. Polymerization was stopped after 1 h. See SI Table S2, entry 21 for other analytical data.

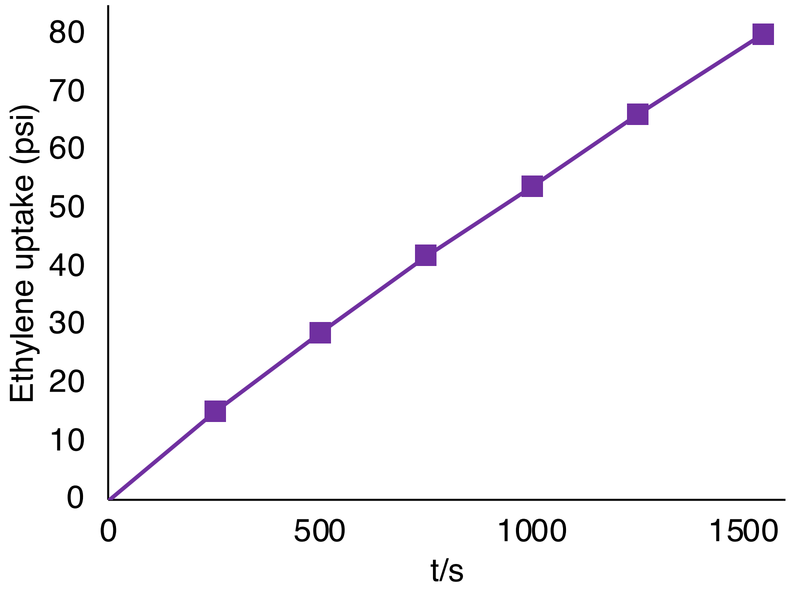

Figure S5. Ethylene uptake curve for 1^{Ph} of copolymerization at high temperature under lower ethylene pressure and high acrylate concentration. Conditions: V = 5 mL, T = 110 °C, [Ni] = 0.05 mM, ethylene pressure = 200 psi, [tBA] = 0.15 M, toluene solvent. Polymerization was stopped after 1 h. See SI Table S2, entry 21 for other analytical data.

1^{Me}

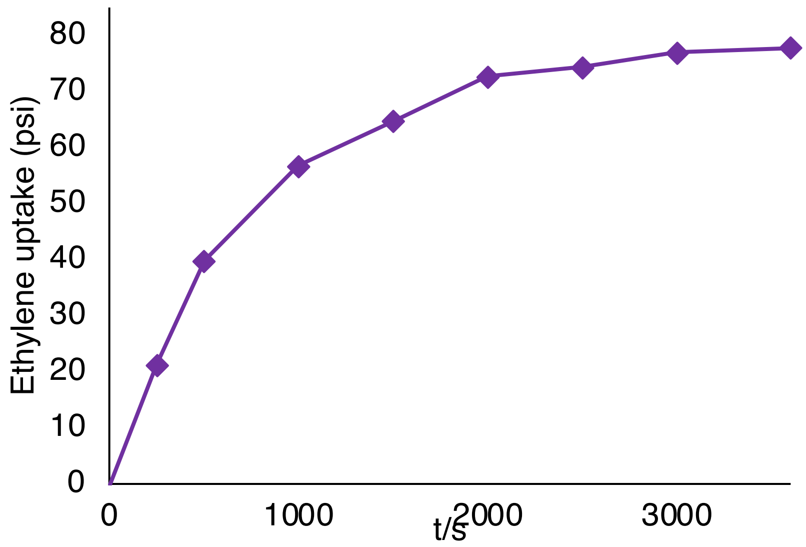

Figure S6. Ethylene uptake curve for 1^{Me} copolymerization at high temperature under moderately high acrylate concentration. Conditions: V = 5 mL, T = 110 °C, [Ni] = 0.05 mM, ethylene pressure = 400 psi, [tBA] = 0.10 M, toluene solvent. Polymerization was stopped after 1 h. See SI Table S2, entry 41 for other analytical data.

5. Characterization of Ethylene/tBA Copolymers

5.1 Microstructural analysis of ethylene/tBA copolymers

The following sections summarize methods and results of microstructural analysis. Analysis of the spectra was based on literature.[4, 11-12]

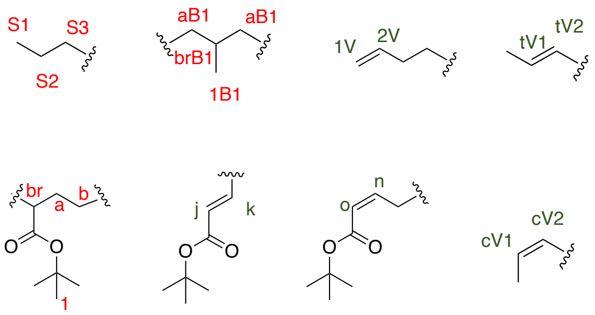

Figure S7. Microstructural features identified in ^{1}H and ^{31}C{^{1}H} NMR analysis.

Copolymer samples **A~F** are ethylene/tBA copolymers produced in entry 1~6 of Table S1, respectively.

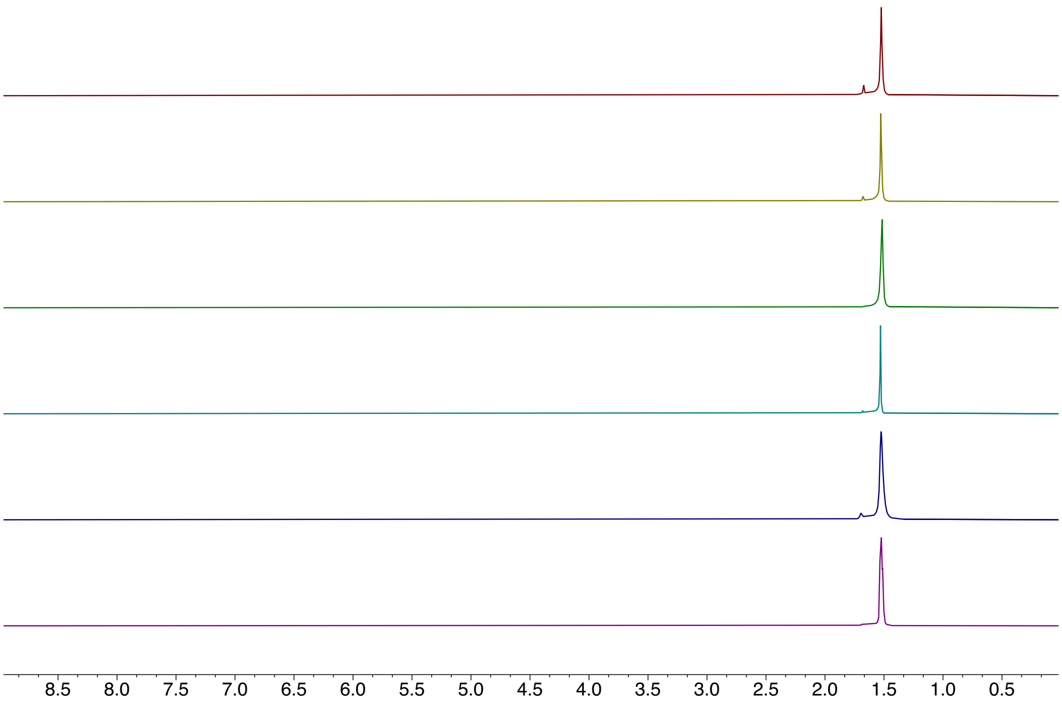

Figure S8. ^{1}H NMR spectra of ethylene/tBA copolymer **A~F** (top to bottom).

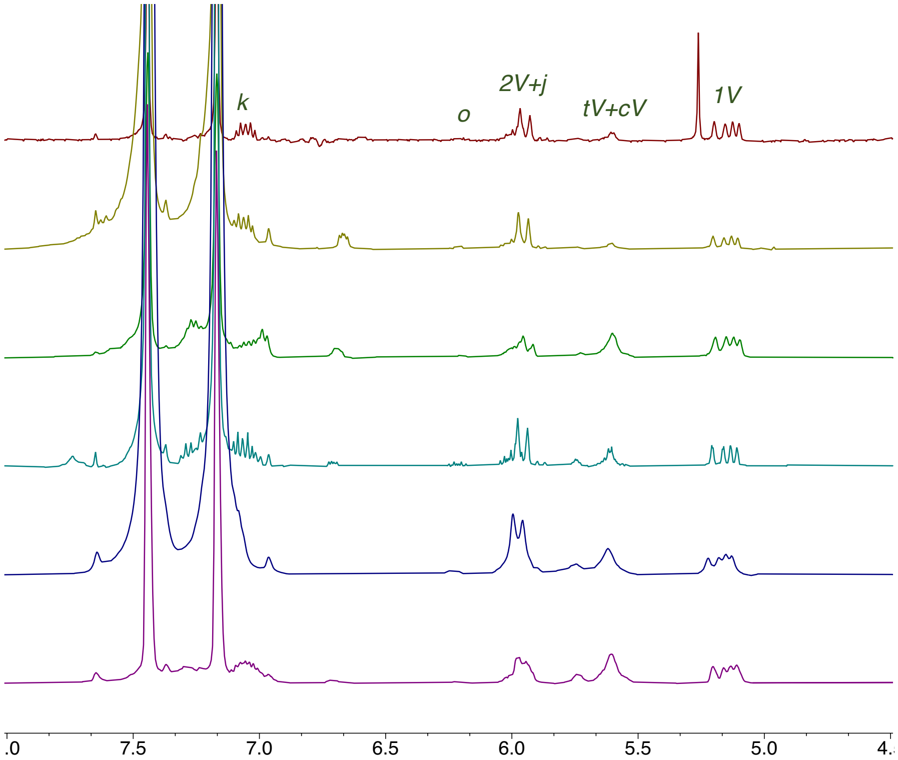

Figure S9. ^{1}H NMR spectra of ethylene/tBA copolymer **A~F** (top to bottom, olefinic region).

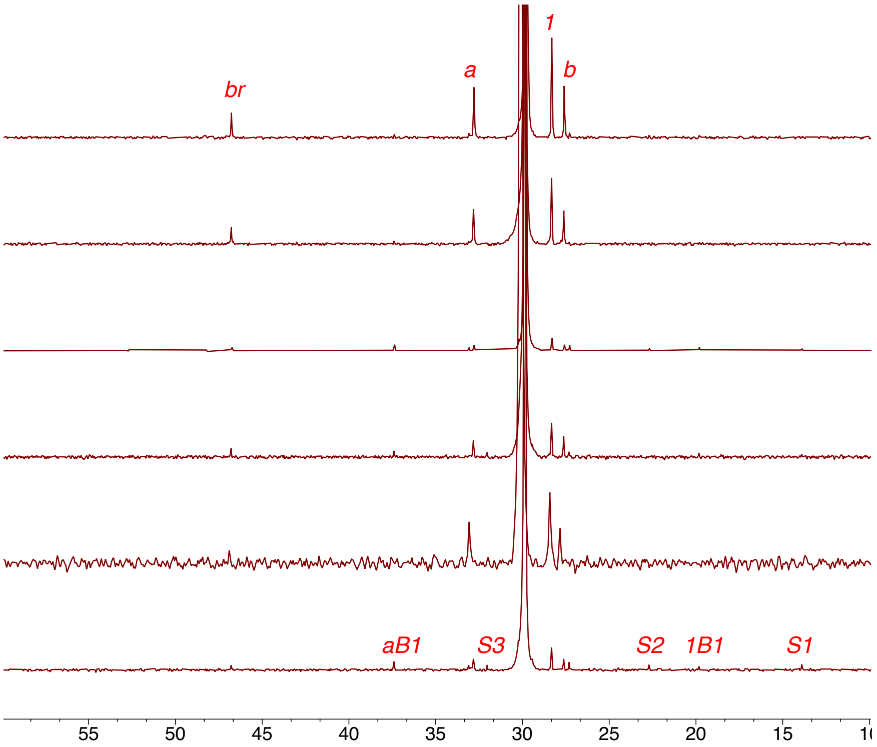

Figure S10. Scaled $^{13}C\{^1H\}$ NMR spectra of ethylene/tBA copolymer **A~F** (top to bottom, alkyl region).

Table S3. Microstructural analysis of ethylene/acrylate copolymers.[a]

	**A**	**B**	**C**	**D**	**E**	**F**
Catalyst	**POP-Ni**	$\mathbf{1^{Me}}$	$\mathbf{1^{Ph}}$	$\mathbf{1^{Ph}}$	$\mathbf{1^{Ph}}$	$\mathbf{1^{Ph}}$
T (°C)	90	90	90	90	90	110
E/psi	400	400	400	400	200	200
[tBA]/M	0.05	0.05	0.05	0.15	0.15	0.05
%Mol tBA	2.1%	1.5%	0.3%	1.0%	1.5%	0.7%
tBA/chain	18	15	1.9	4.2	5.5	1.5
Terminal tBA/ chain	0.53	0.69	0.23	0.58	0.66	0.37
%Terminal tBA/ tBA	2.9%	4.6%	12.1%	14.0%	12.1%	27.2%
Vinyl/chain	0.39	0.24	0.44	0.25	0.18	0.29
2-Propenyl/ chain	0.08	0.06	0.33	0.16	0.16	0.31
Vinyl+propenyl/E units[b]	0.06%	0.03%	0.13%	0.09%	0.10%	0.26%

[a] Copolymer samples **A~F** are ethylene/tBA copolymers produced in entry 1~6 of Table S1, respectively. See SI section S4 for more details. [b] 'Vinyl+propenyl': the sum of numbers of the vinyl and 2-propenyl units per chain. 'E units': the number of ethylene units in one chain.

6. Catalyst Comparison

A variety of catalysts have been developed for ethylene/acrylate copolymerization and their performance are summarized in Figure S11, with two metrics being plotted: catalyst activity and tBA incorporation.[4, 6, 11-99] Previously reported ethylene/acrylate copolymerization experiments were included if they feature activity > 0.5 kg/(mol*h), copolymer Mw>2500 and %mol tBA > 0.1%. In addition, experiments are excluded if they were performed with activator/masking reagents on a scale comparable to the amount of acrylate (additives:acrylates > 1:10). Overall, 573 different experiments with 281 different catalysts from 91 scientific papers are included.[4, 6, 11-99] Pd examples are shown in grey triangles while Ni examples are shown in squares with colors referring to Mw of resulting copolymers. Reaction conditions, such the ethylene pressure, catalyst, and monomer concentration, may differ, therefore these comparisons should be considered qualitative.

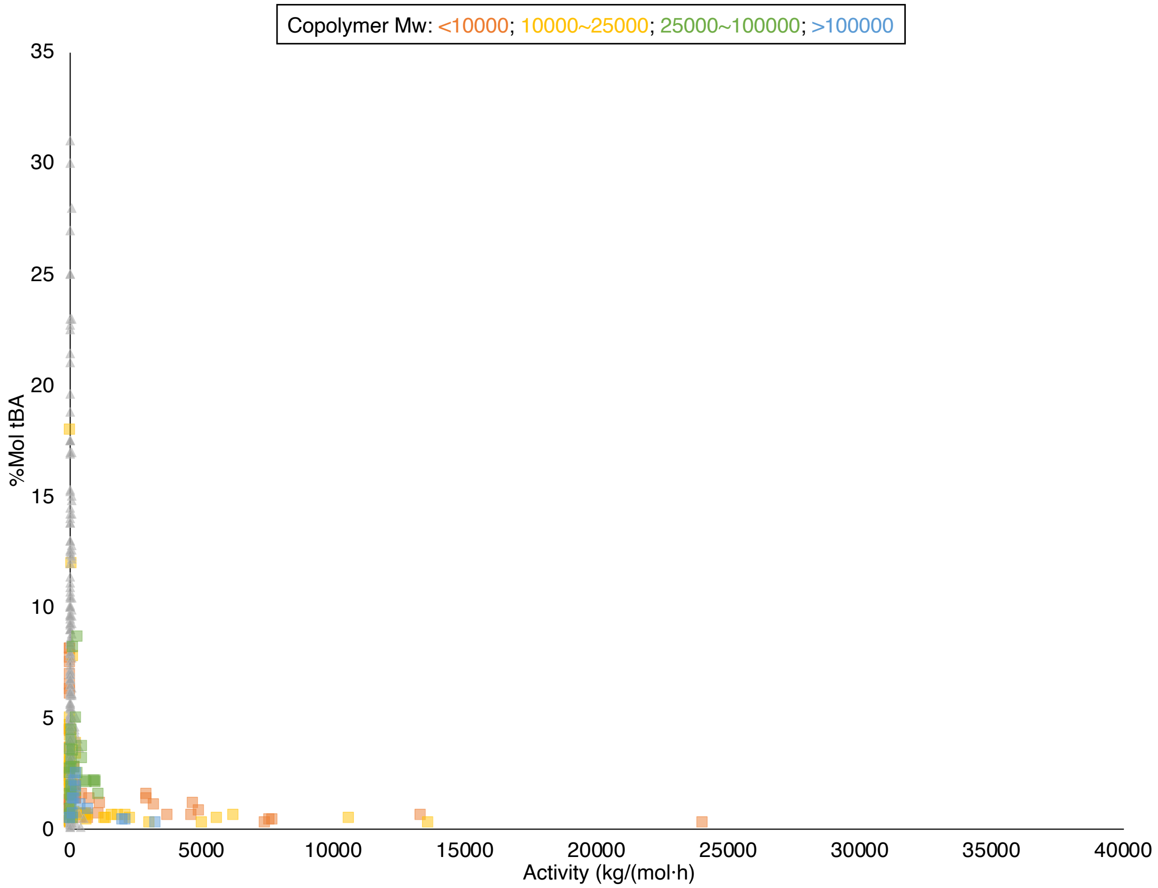

Figure S11. Reported Pd and Ni catalysts for ethylene/acrylate copolymerization (Grey triangles: palladium examples; squares: nickel examples; darker color indicates overlapping data points.

As shown in Figure S11, reported Pd catalysts produce copolymers with a broad range of acrylate incorporation, with relatively low activity (typically <100 kg/(mol*h). In contrast, Ni catalysts produce copolymers with limited amount of acrylate incorporation(typically <5%), but many of them feature higher activity. It's also notable that the most active catalysts are only able to produce copolymers with Mw < 25000.

Performance of two catalysts reported in this manuscript, $\mathbf{1^{Me}}$ and $\mathbf{1^{Ph}}$, are included in Figure S12, in circles and spheres with color referring to the Mw of resulting copolymers, respectively. In general, $\mathbf{1^{Ph}}$, not only features state-of-art activity, but also represent the only example that is able to produce copolymers with Mw>25000. Despite this, further progress is needed to combine high activity and high acrylate incorporation.

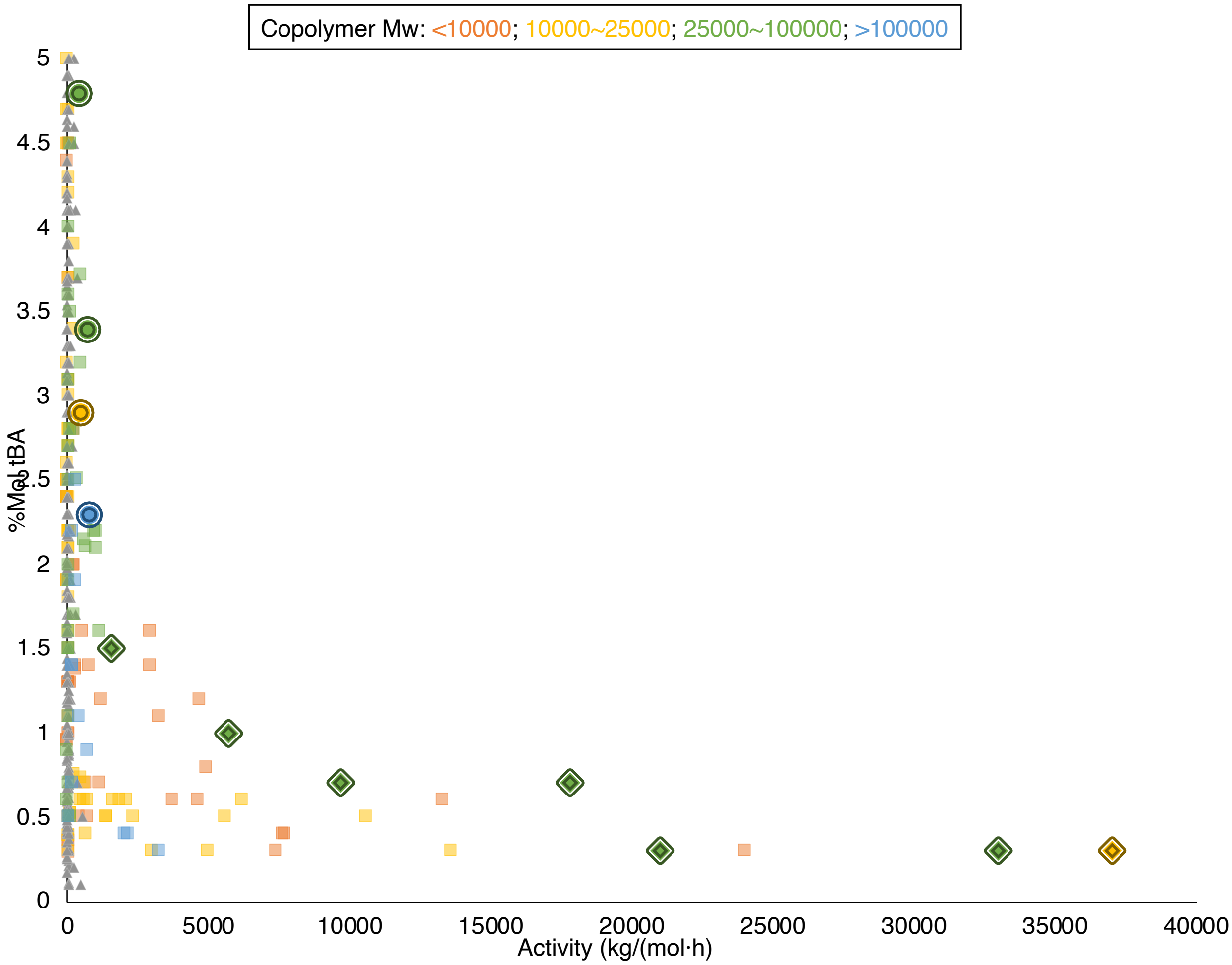

Figure S12. Performance of $\mathbf{1^{Me}}$ (circles) and $\mathbf{1^{Ph}}$ (diamonds) in comparison with reported Pd and Ni catalysts for ethylene/acrylate copolymerization (Grey triangles: palladium examples; squares: nickel examples; darker color indicates overlapping data points).

Figure 13 lists catalyst activities at different temperatures. The more active catalyst, **1^{Ph}**, show state-of-art thermal stability. In addition, **1^{Me}** and **1^{Ph}** are the only examples to date that produce copolymers with Mw>25000 at 110 °C.

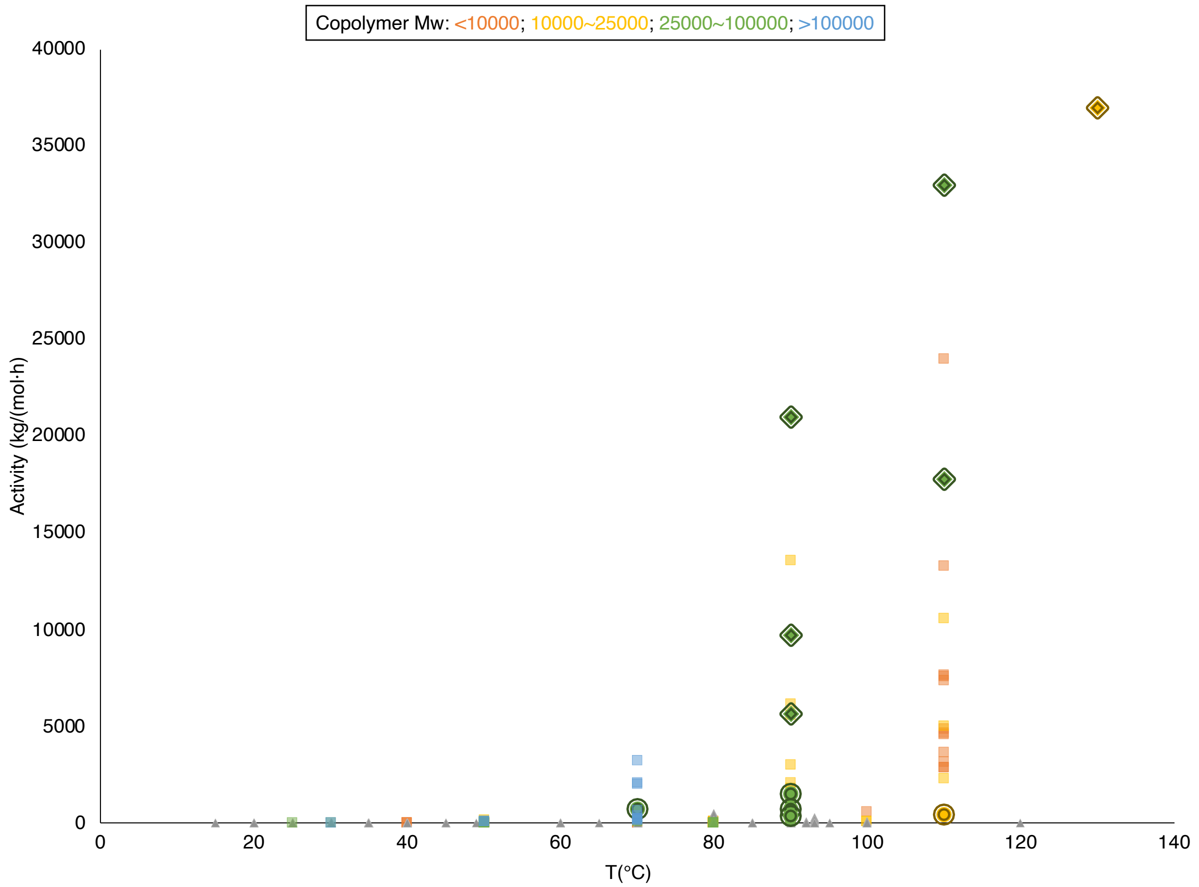

Figure S13. Temperature-dependent performance of **1^{Me}** (circles) and **1^{Ph}** (diamonds) in comparison with reported Pd and Ni catalysts for ethylene/acrylate copolymerization (Grey triangles: palladium examples; squares: nickel examples; darker color indicates overlapping data points).

7. Investigations of Acrylate-Induced Reactions

Procedures. Unless specified, 0.0059 mmol of the Ni catalyst prepared using the above procedure was dissolved in C_6D_6 and transferred to a J-Young tube. The solution was frozen in the Coldwell pre-cooled by a liquid nitrogen bath, and *t*-butyl acrylate (tBA) was added via syringe (Total volume = 0.50 ml). The resulting mixture was warmed up to thawing temperature and shaken vigorously prior to transferring to pre-heated NMR probe for acquisition of spectra at 25 °C. NMR monitoring of tBA insertion were performed by monitoring the ^{1}H and $^{31}P\{^{1}H\}$ NMR.

tBA insertion into $\mathbf{1^{Ph}}$

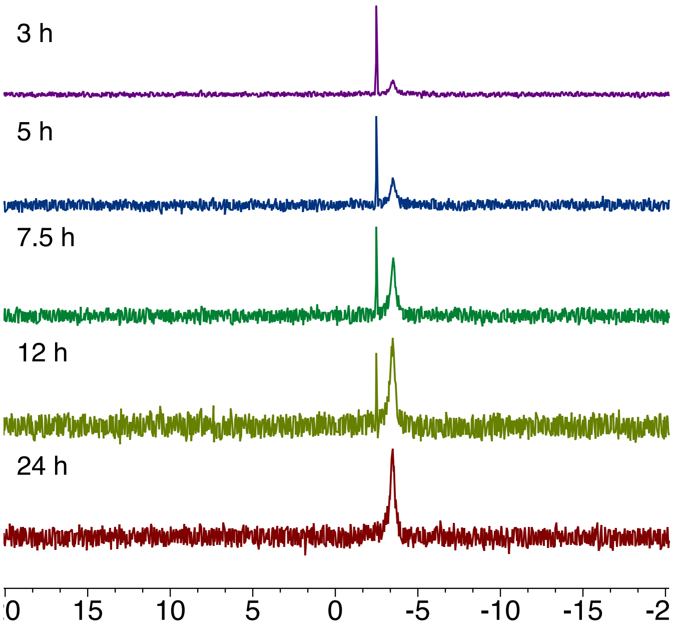

Figure S14. $^{31}P\{^{1}H\}$ NMR monitoring of reaction of tBA with $\mathbf{1^{Ph}}$ (Condition: [$\mathbf{1^{Ph}}$] = 0.0118 M, [tBA] = 0.177 M, solvent: C_6D_6, V = 0.5 mL).

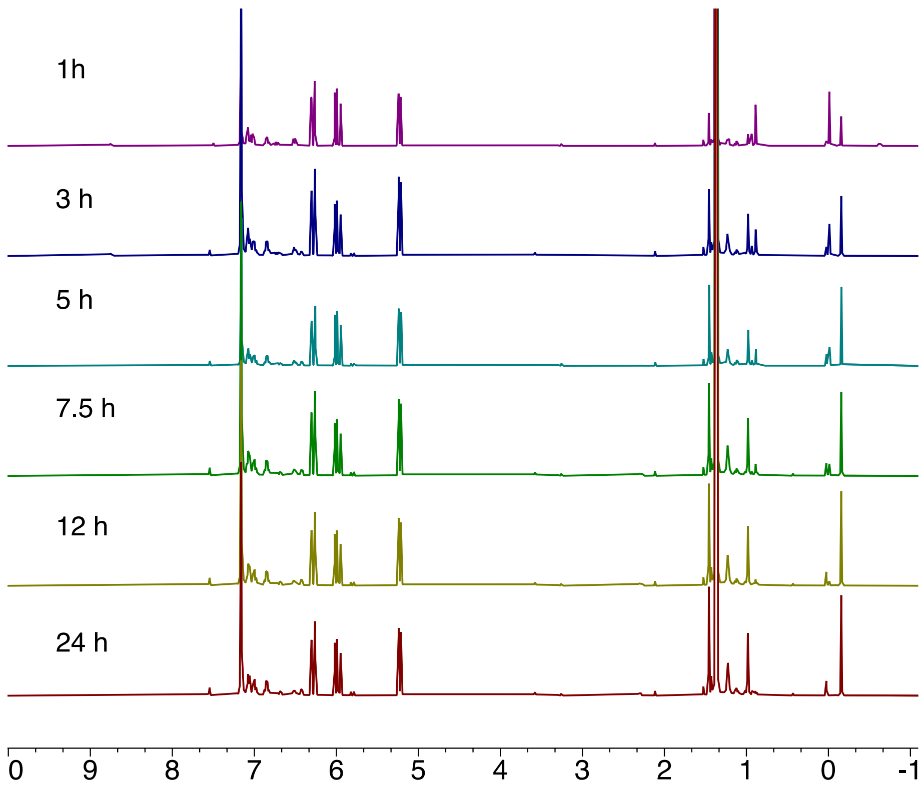

Figure S15. ^{1}H NMR monitoring of reaction of tBA with **1^{Ph}** (Condition: [**1^{Ph}**] = 0.0118 M, [tBA] = 0.177 M, solvent: C_6D_6, V = 0.5 mL).

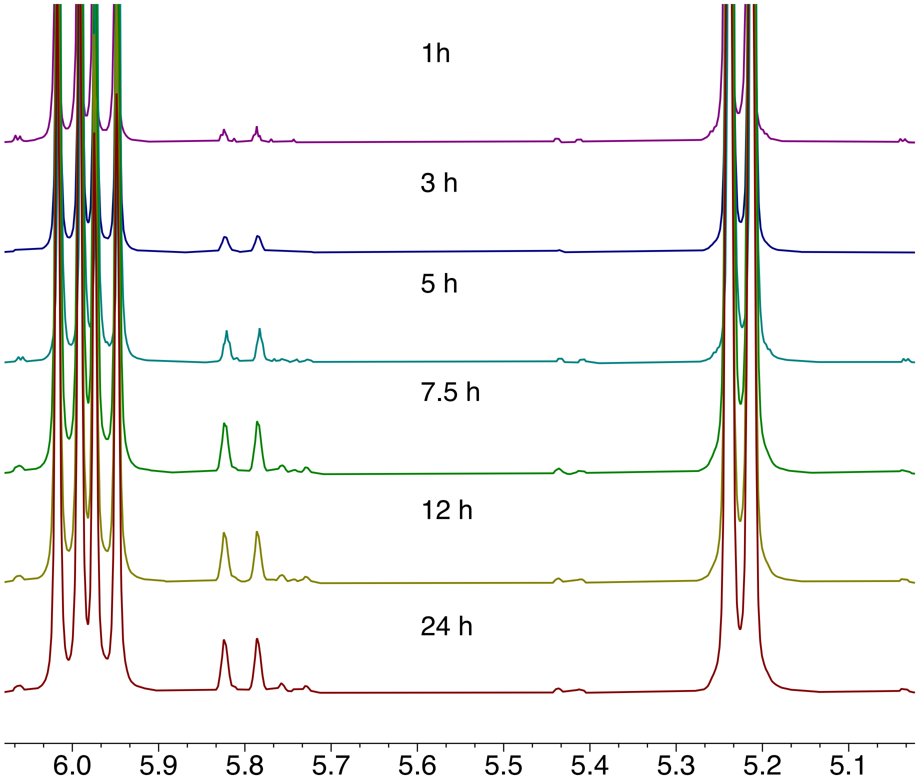

Figure S16. ^{1}H NMR monitoring of reaction of tBA with $\mathbf{1^{Ph}}$ (Olefinic region, condition: [$\mathbf{1^{Ph}}$] = 0.0118 M, [tBA] = 0.177 M, solvent: C_6D_6, V = 0.5 mL).

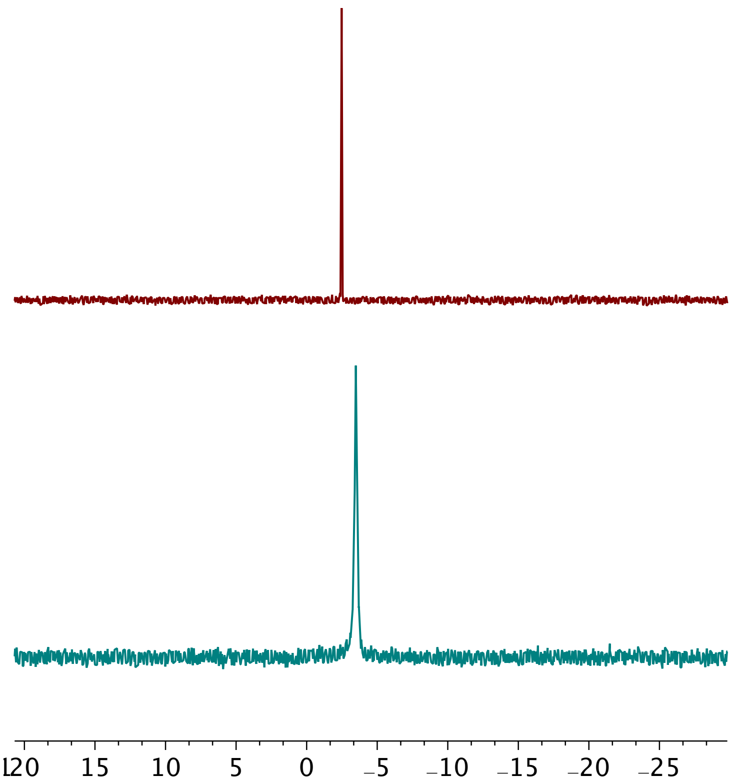

Figure S17. $^{31}P\{^{1}H\}$ NMR spectra of **$7^{PhO}PO^{Br}$-Ni** (top) and tBA inserted products (bottom, after removal of volatiles)

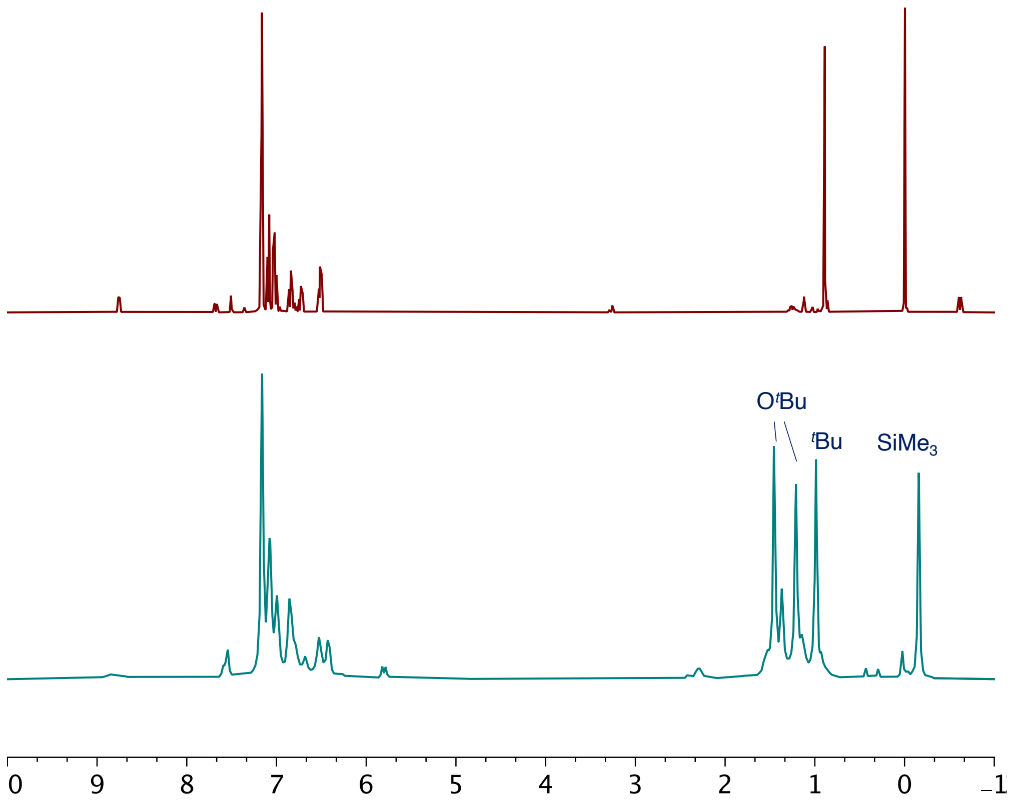

Figure S18. $^{31}P\{^1H\}$ NMR spectra of **1^{Ph}** (top) and tBA inserted products (bottom, after removal of volatiles)

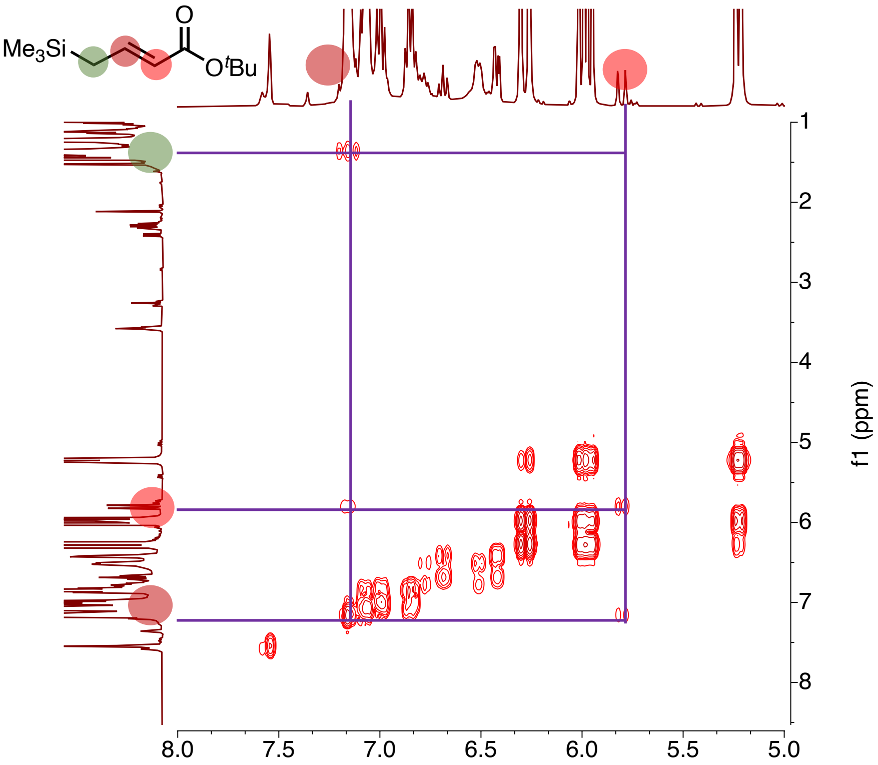

Figure S19. Identifying the internal olefin: ^{1}H-^{1}H COSY NMR spectrum of products generated from tBA reaction with **1**$^{\mathbf{Ph}}$.

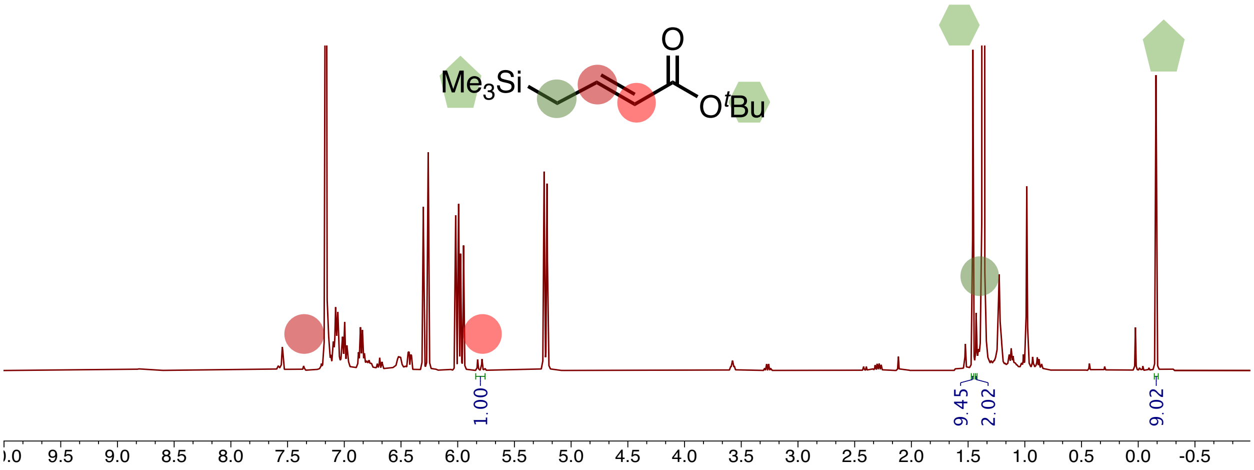

Figure S20. Identifying the internal olefin: ^{1}H NMR characterization of the internal olefin from the mixture generated from tBA reaction with **1**$^{\mathbf{Ph}}$.

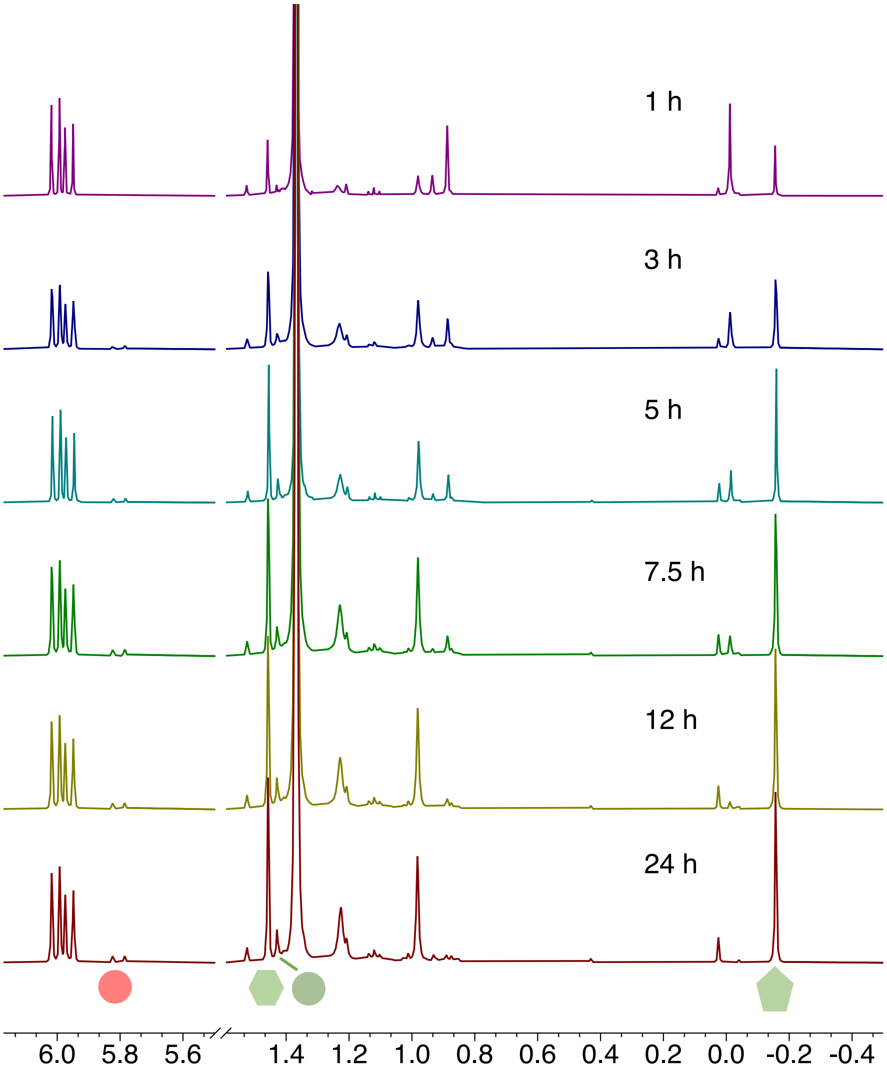

Figure S21. ^{1}H NMR monitoring of generation of the internal olefin overtime (Condition: [$\mathbf{1^{Ph}}$] = 0.0118 M, [tBA] = 0.177 M, solvent: C_6D_6, V = 0.5 mL).

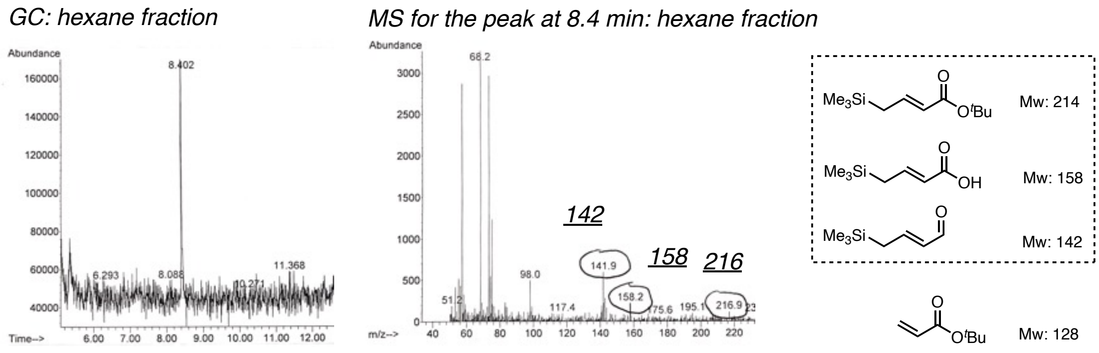

Figure S22. Identifying the internal olefin: GC-mass analysis of the mixture generated from tBA reaction with $\mathbf{1^{Ph}}$.

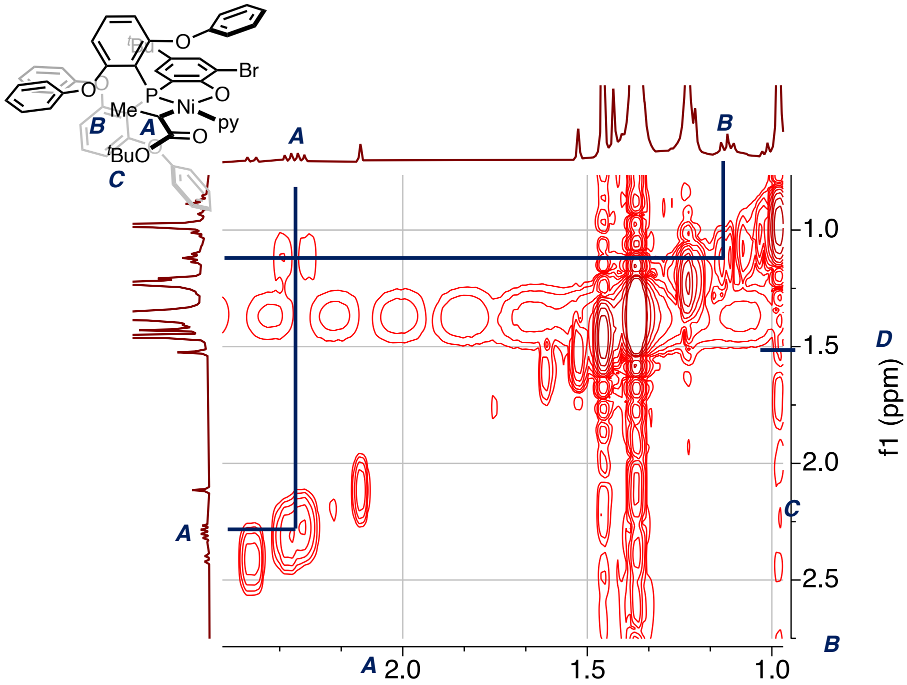

Figure S23. Identifying the tBA reinsertion product ($\mathbf{4^{Ph\text{-}tBu}}$): ^{1}H-^{1}H COSY NMR spectrum of products generated from tBA reaction with $\mathbf{1^{Ph}}$.

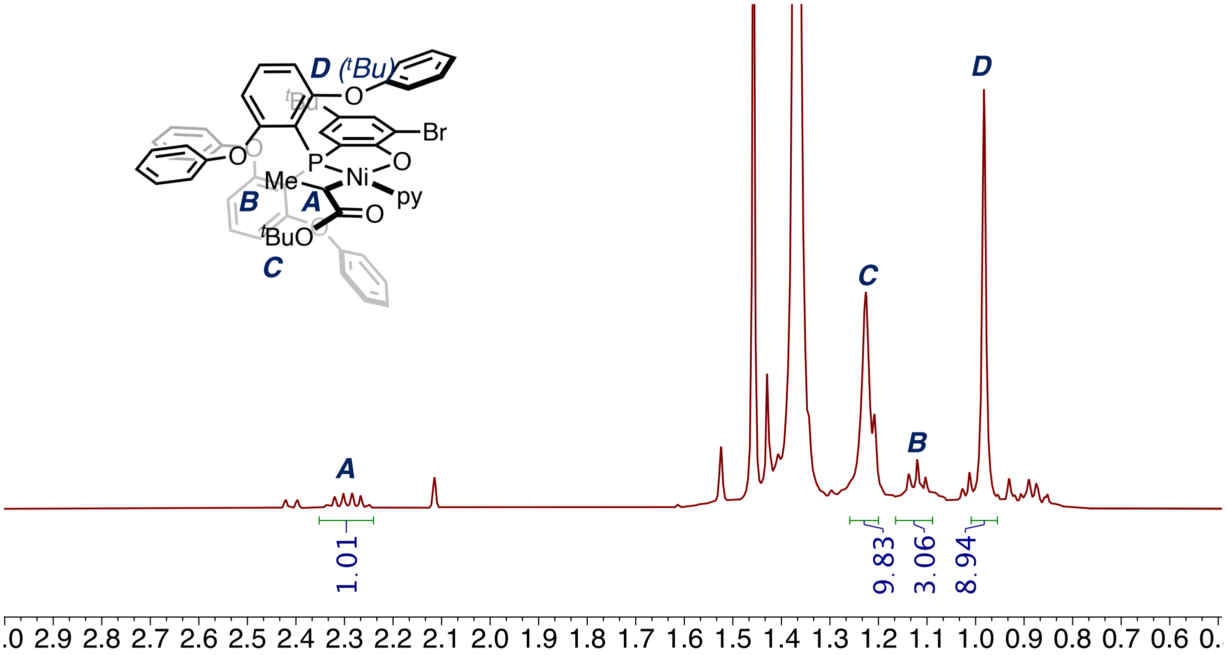

Figure S24. Identifying the tBA reinsertion product ($\mathbf{4^{Ph\text{-}tBu}}$): ^{1}H NMR spectrum of products generated from tBA reaction with $\mathbf{1^{Ph}}$.

*nBA insertion into **1**^{Ph}*

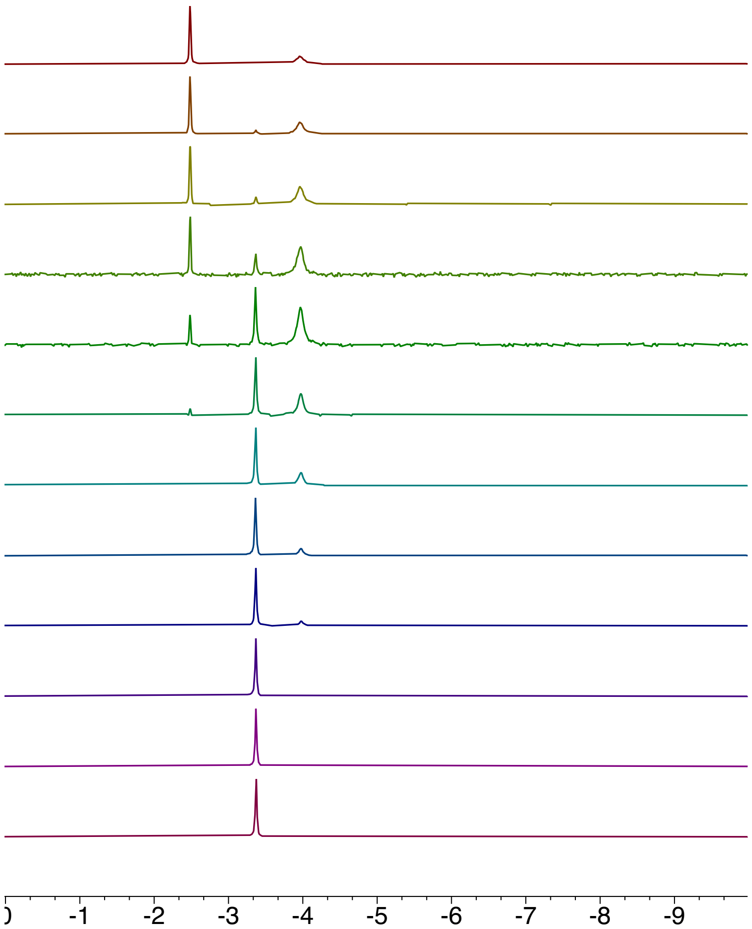

Figure S25. $^{31}P\{^1H\}$ NMR monitoring of reaction of nBA with $\mathbf{1}^{Ph}$ (Condition: [$\mathbf{1}^{Ph}$] = 0.0118 M, [tBA] = 0.177 M, solvent: C_6D_6, V(total) = 0.5 mL, T = 25 °C. Top to bottom: t = 10, 18, 31, 60, 105, 153, 228, 328, 419, 591, 700 min).

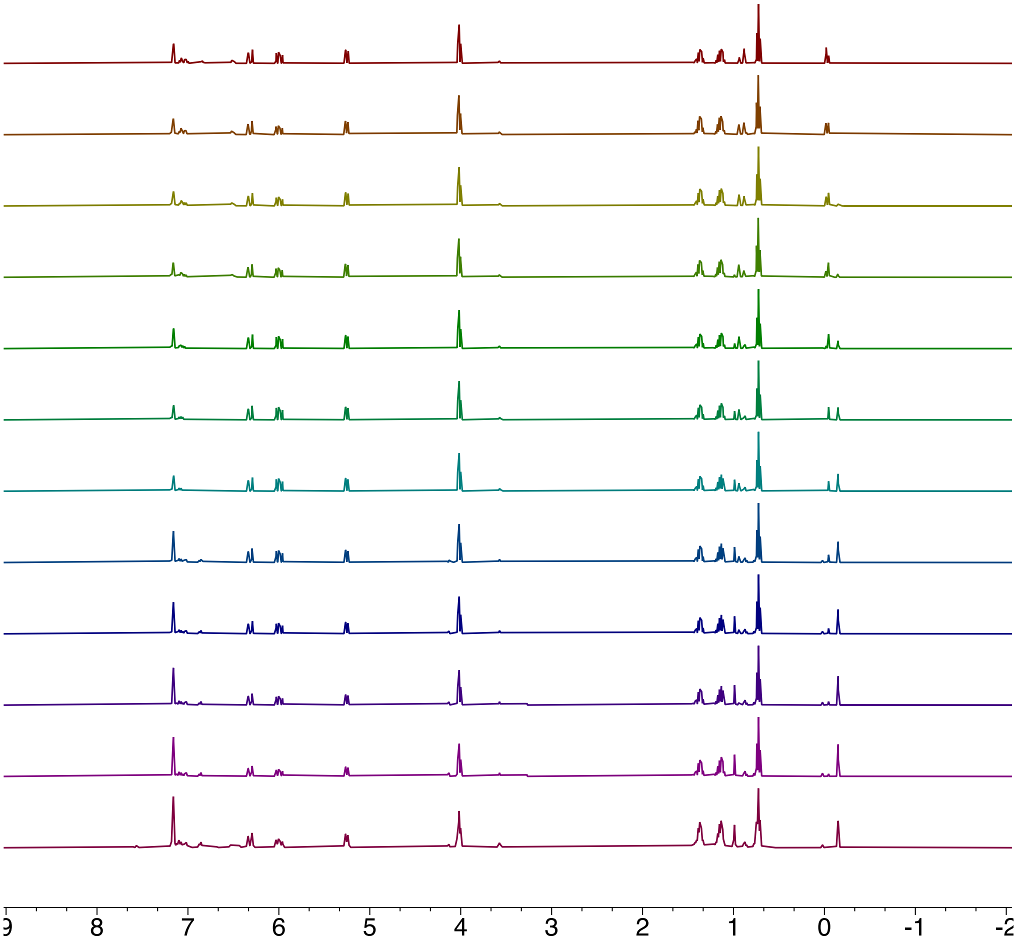

Figure S26. ^{1}H NMR monitoring of reaction of nBA with $\mathbf{1^{Ph}}$ (Condition: [$\mathbf{1^{Ph}}$] = 0.0118 M, [tBA] = 0.177 M, solvent: C_6D_6, V(total) = 0.5 mL, T = 25 °C. Top to bottom: t = 8, 16, 29, 58, 103, 151, 226, 326, 417, 589, 697 min).

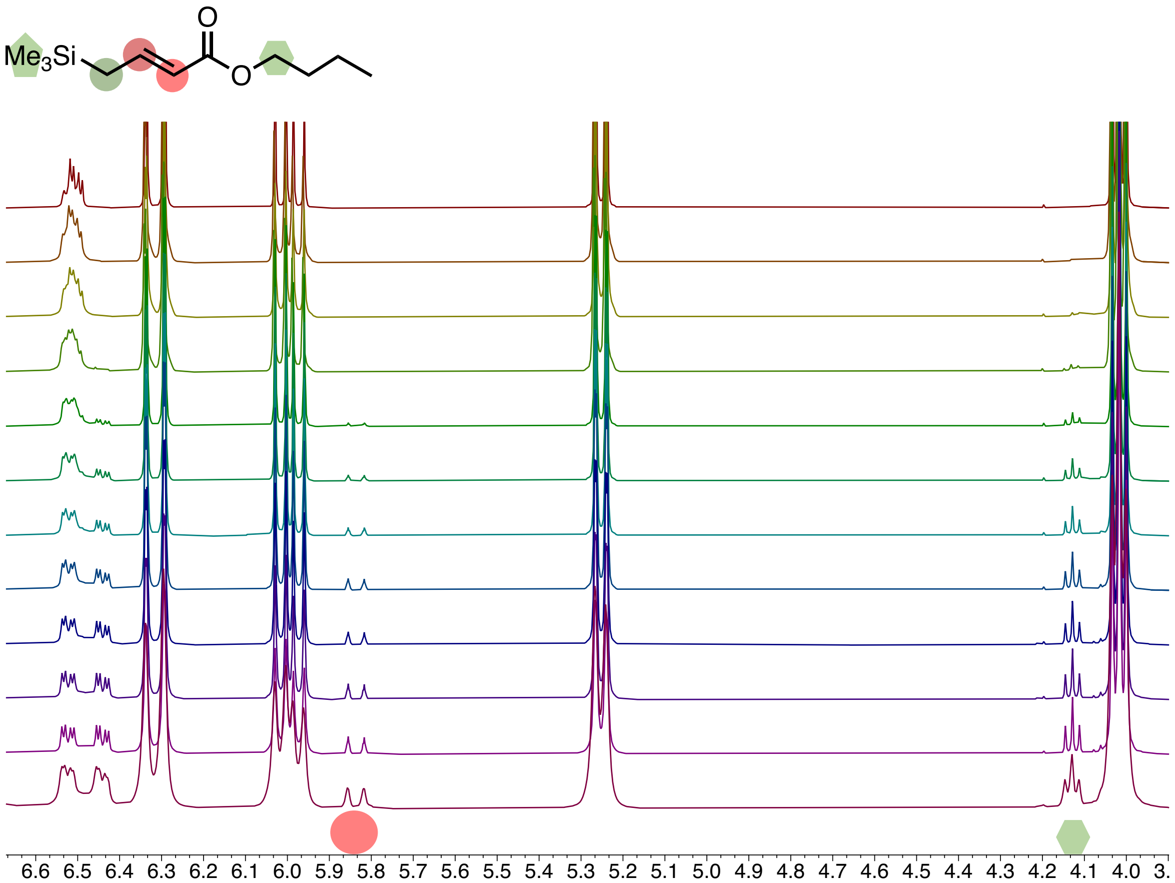

Figure S27. ^{1}H NMR monitoring of generation of the internal olefin over time (Condition: [**1**$^{\mathbf{Ph}}$] = 0.0118 M, [tBA] = 0.177 M, solvent: C_6D_6, V = 0.5 mL).

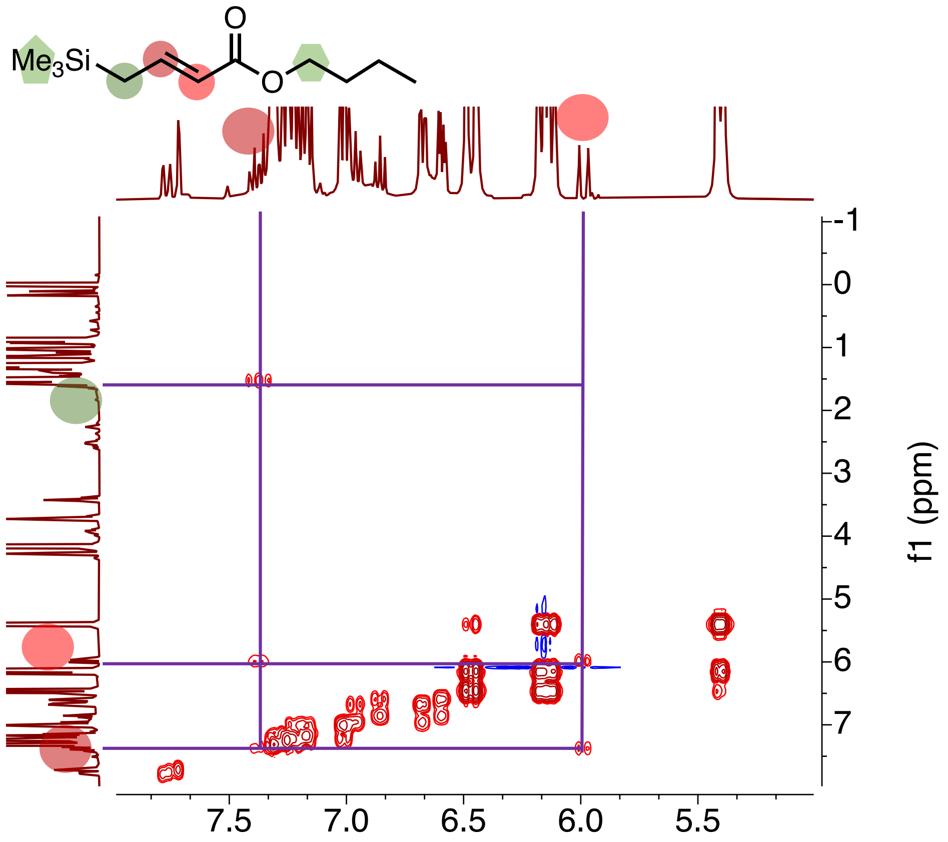

Figure S28. Identifying the internal olefin: ¹H-¹H COSY NMR spectrum of products generated from nBA reaction with **1**^{Ph}.

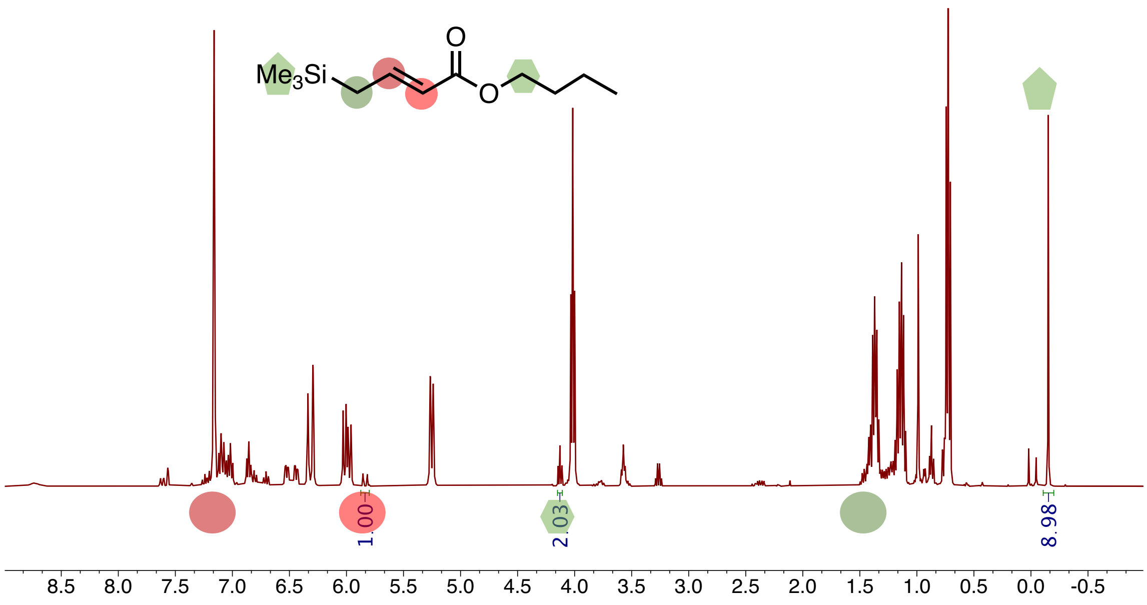

Figure S29. Identifying the internal olefin: ¹H NMR characterization of the internal olefin from the mixture generated from tBA reaction with **1**^{Ph}.

*MA insertion into **1**^Ph^*

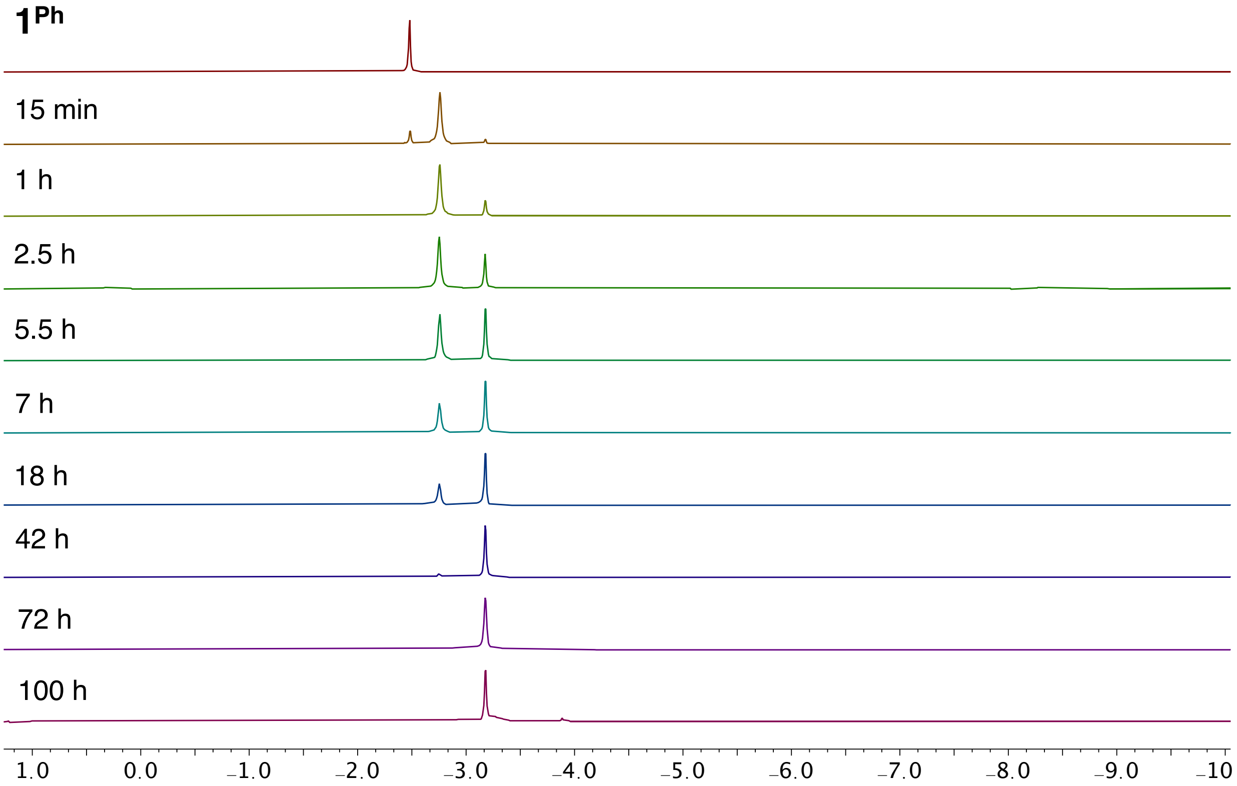

Figure S30. $^{31}P\{^{1}H\}$ NMR monitoring of reaction of MA with $\mathbf{1}^{\mathbf{Ph}}$ (Condition: [$\mathbf{1}^{\mathbf{Ph}}$] = 0.0118 M, [MA] = 0.177 M, solvent: C_6D_6, V = 0.5 mL).

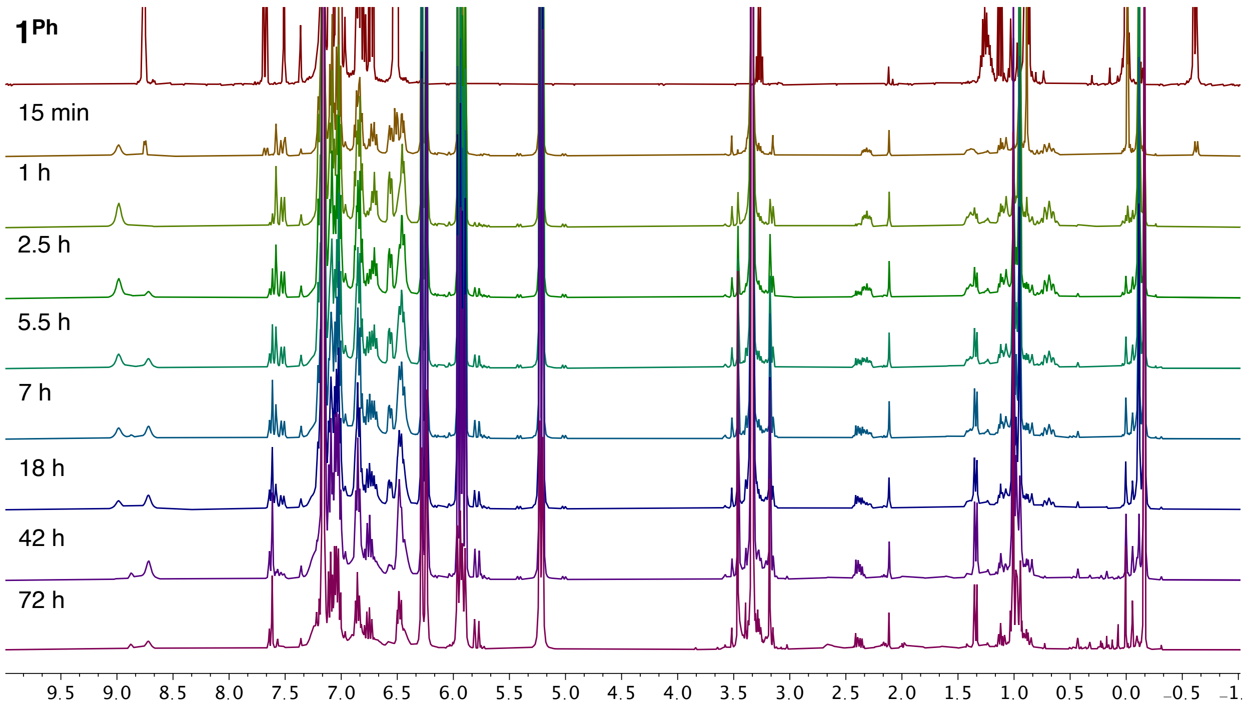

Figure S31. ^1H NMR monitoring of reaction of MA with $\mathbf{1^{Ph}}$ (Condition: [$\mathbf{1^{Ph}}$] = 0.0118 M, [MA] = 0.177 M, solvent: C_6D_6, V = 0.5 mL).

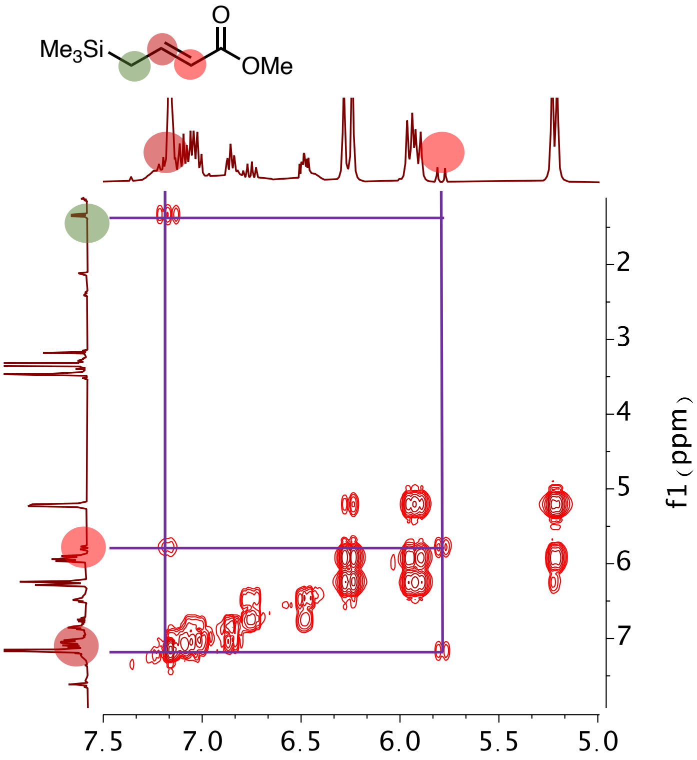

Figure S32. Identifying the internal olefin: ^{1}H-^{1}H COSY NMR spectrum of products generated from MA reaction with **1^{Ph}**.

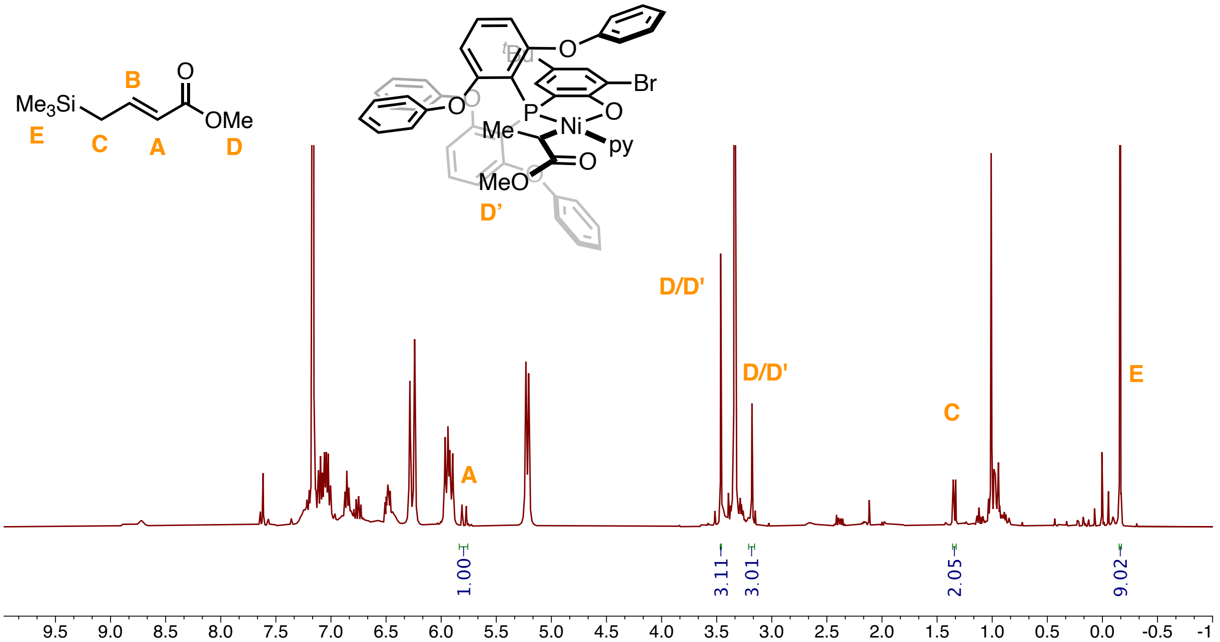

Figure S33. Identifying the internal olefin and **4$^{Ph\text{-}Me}$**: ^{1}H NMR characterization of the internal olefin from the mixture generated from MA reaction with **1^{Ph}**.

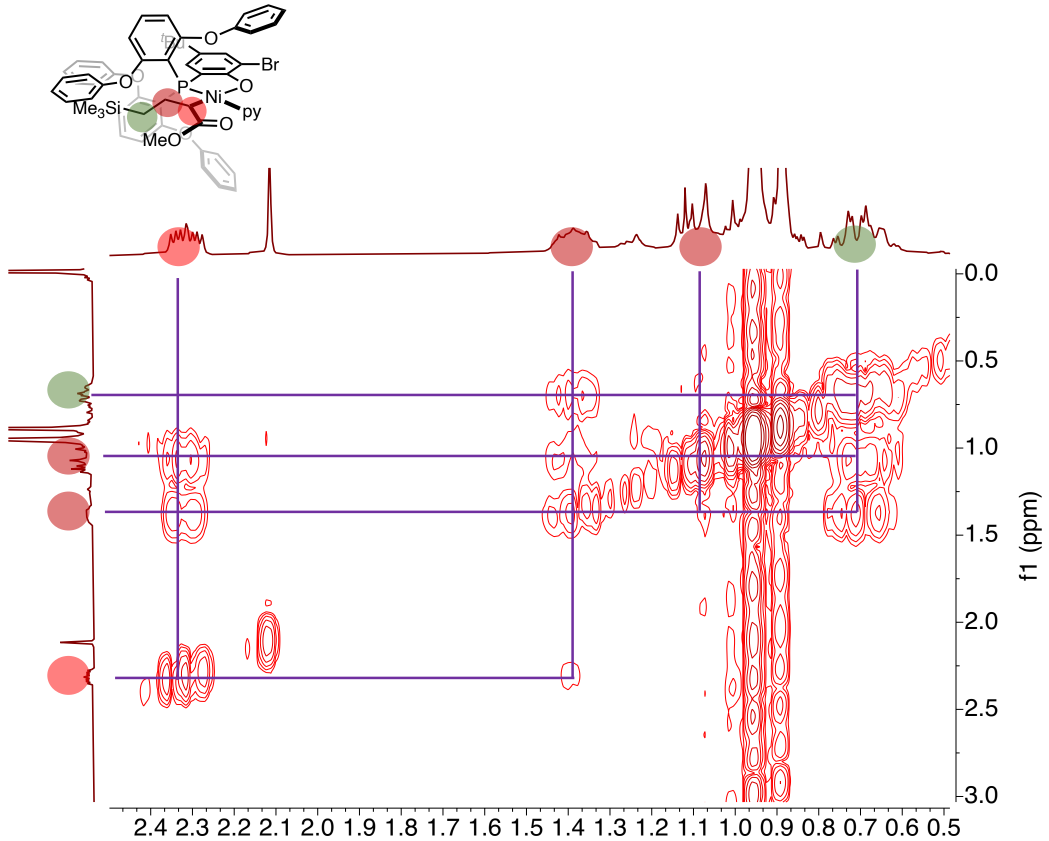

Figure S34. Identifying the first MA product ($\mathbf{2^{Ph\text{-}Me}}$): ^{1}H-^{1}H COSY NMR spectrum of tBA reaction with $\mathbf{1^{Ph}}$ (15 min).

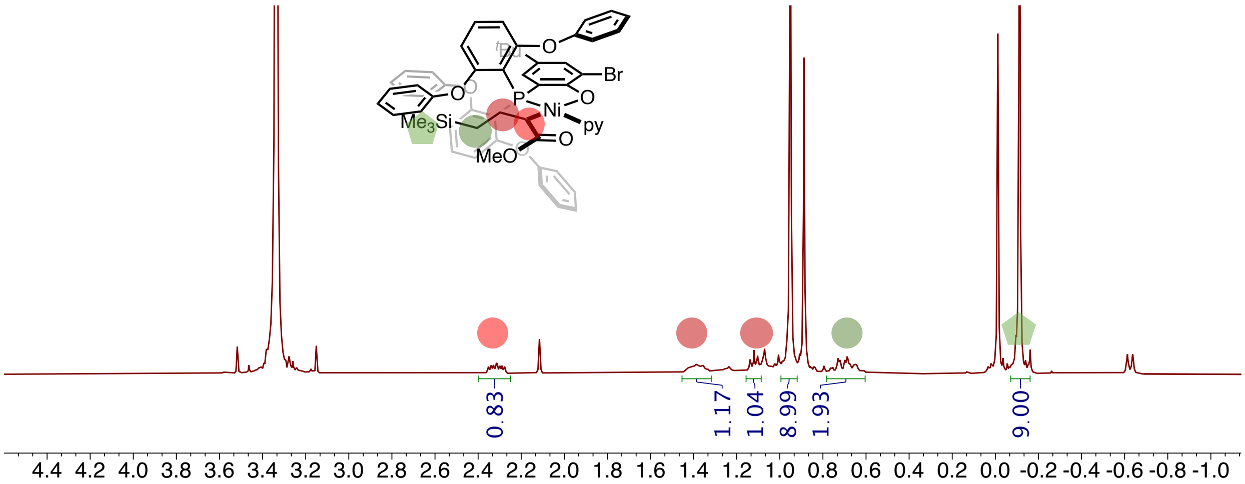

Figure S35. Identifying the first MA product ($\mathbf{2^{Ph\text{-}Me}}$): ^{1}H NMR spectrum of products generated from tBA reaction with $\mathbf{1^{Ph}}$.

*tBA insertion into **1**^{Me}*

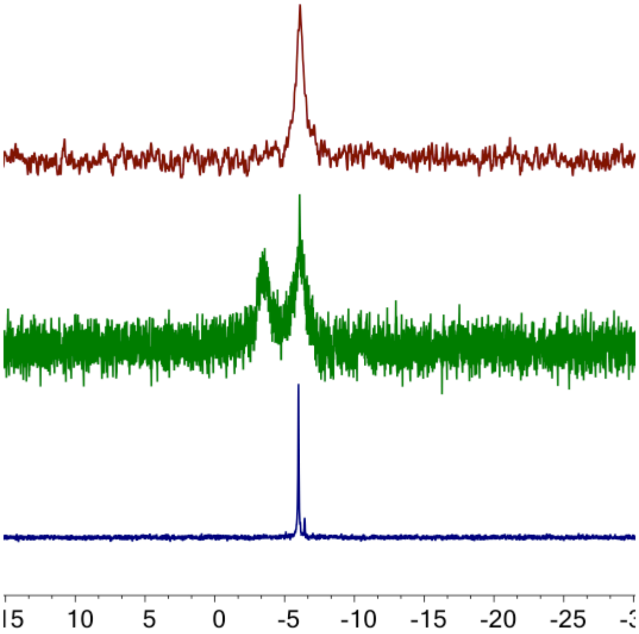

Figure S36. $^{31}P\{^1H\}$ NMR spectra of **1**^{Me} + 50 tBA (t = 75 min), residue after removal of volatiles (medium) and the product after addition of 2 equiv. of pyridine (bottom).

Addition of excess tBA to **1**^{Me} leads to generation of new species featuring one broad resonance in $^{31}P\{^1H\}$ NMR spectrum (Figure S36), which are converted to two broad resonances after removal of volatiles. After addition of 2 equiv. of pyridine, these new broad resonances collapse to one sharp resonance.

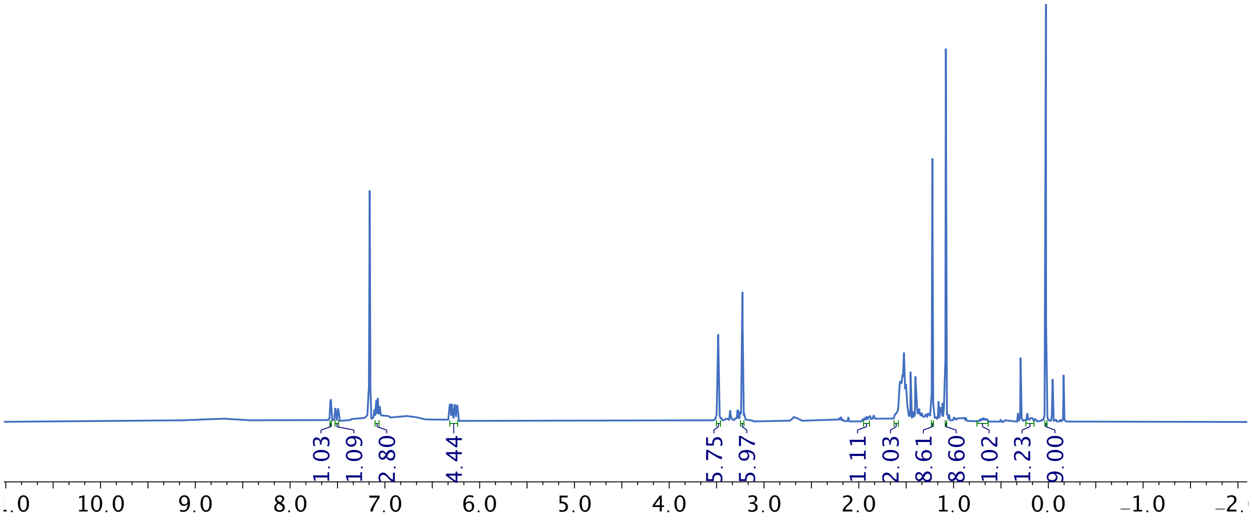

Figure S37. ^1H NMR spectrum of the tBA insertion product (**2**$^{Me\text{-}tBu}$) with 2 equiv. of pyridine.

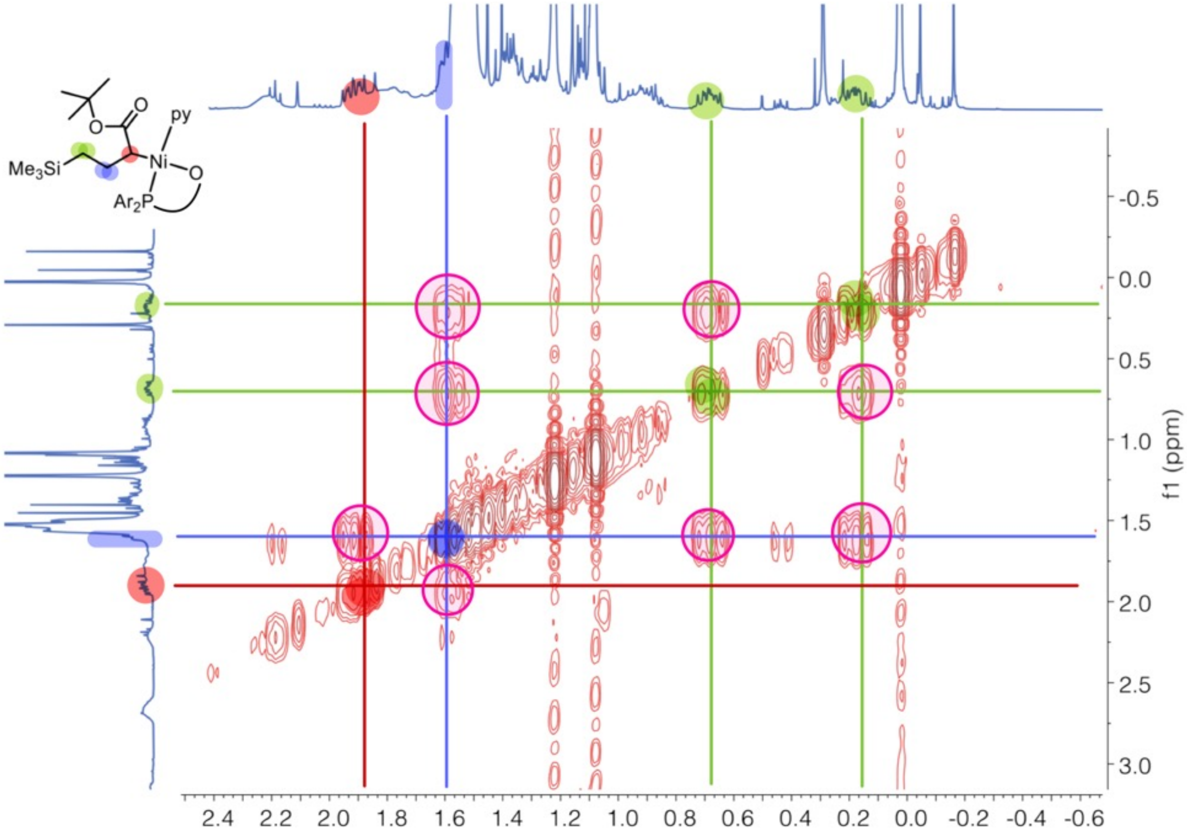

Figure S38. ^1H-^1H COSY NMR spectrum of tBA insertion product ($\mathbf{2}^{\mathbf{Me\text{-}tBu}}$) with 2 equiv. of pyridine.

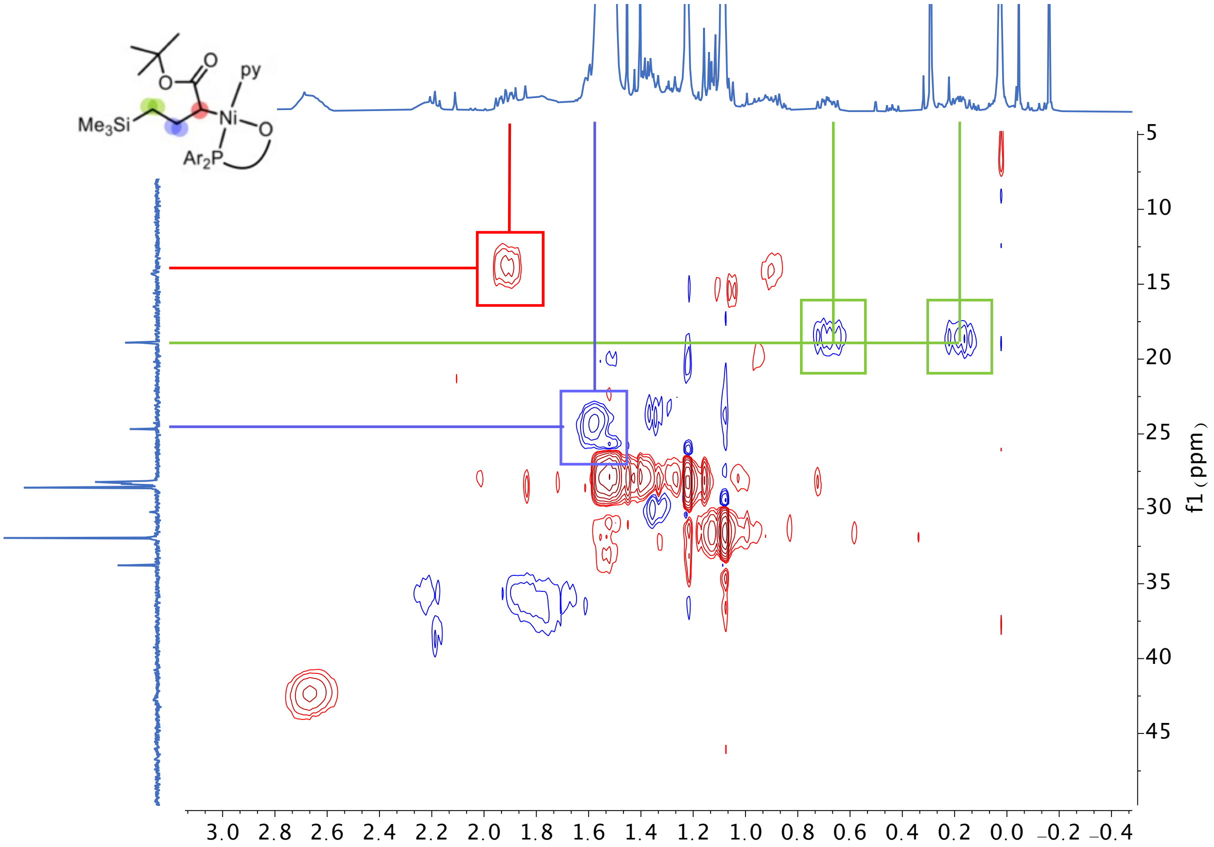

Figure S39. ^{1}H-^{13}C HSQC NMR spectrum of tBA insertion product (**2$^{\text{Me-tBu}}$**) with 2 equiv. of pyridine.

Based on ^{1}H, ^{1}H-^{1}H COSY, ^{1}H-^{13}C HSQC NMR analysis (Figure S37-39), the major product was identified as **2$^{\text{Me-tBu}}$**, the species generated after first tBA insertion.

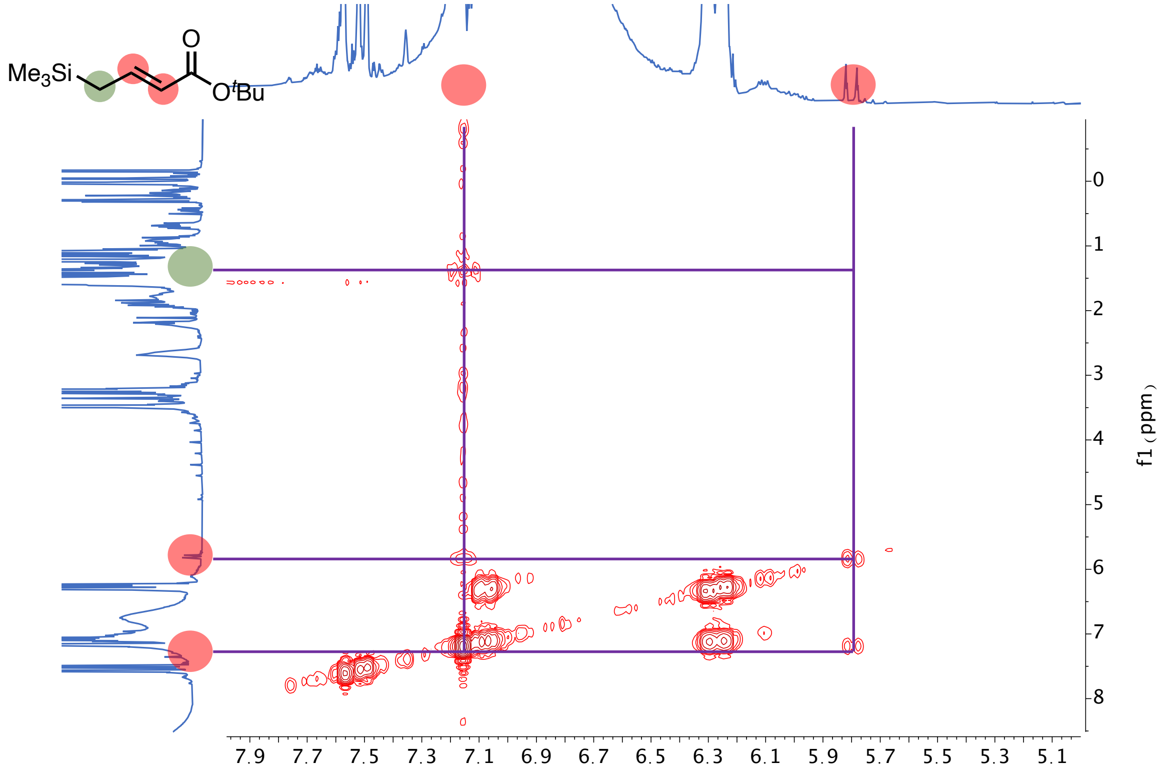

Figure S40. Possible observation of the internal olefin: ^{1}H-^{1}H COSY NMR spectrum of tBA insertion product ($\mathbf{2}^{\mathbf{Me\text{-}tBu}}$) with 2 equiv. of pyridine.

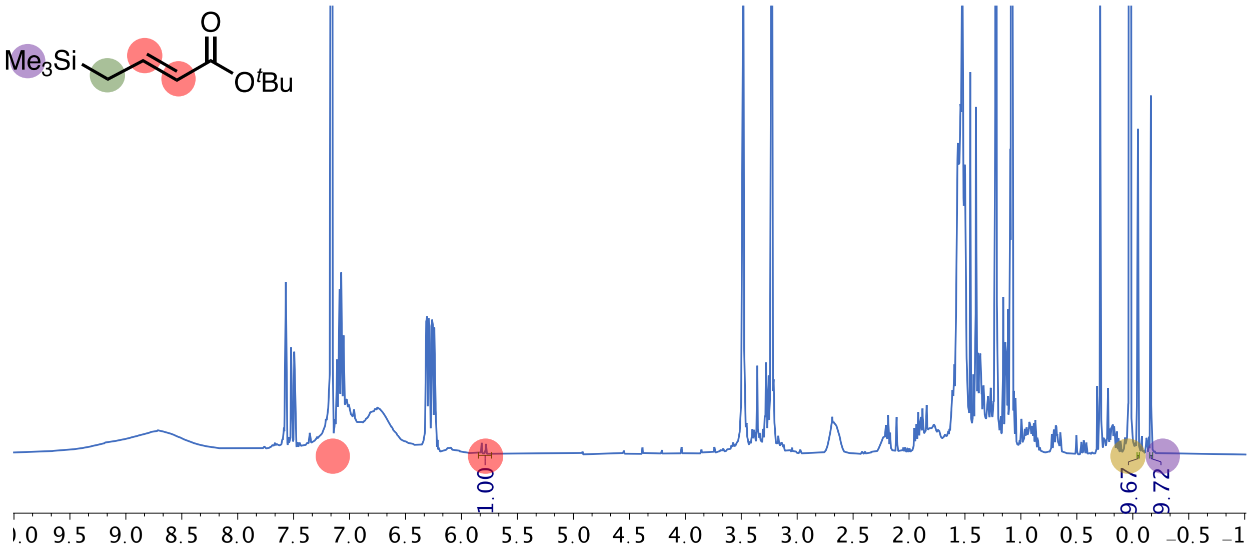

Figure S41. Possible observation of the internal olefin: ^{1}H NMR spectrum of the tBA insertion product ($\mathbf{2}^{\mathbf{Me\text{-}tBu}}$) with 2 equiv. of pyridine.

Though $\mathbf{2}^{\mathbf{Me\text{-}tBu}}$ is the major product (c.a. >90% NMR yields), other minor species were also observed during this reaction, including the internal olefin generated from β-H elimination.

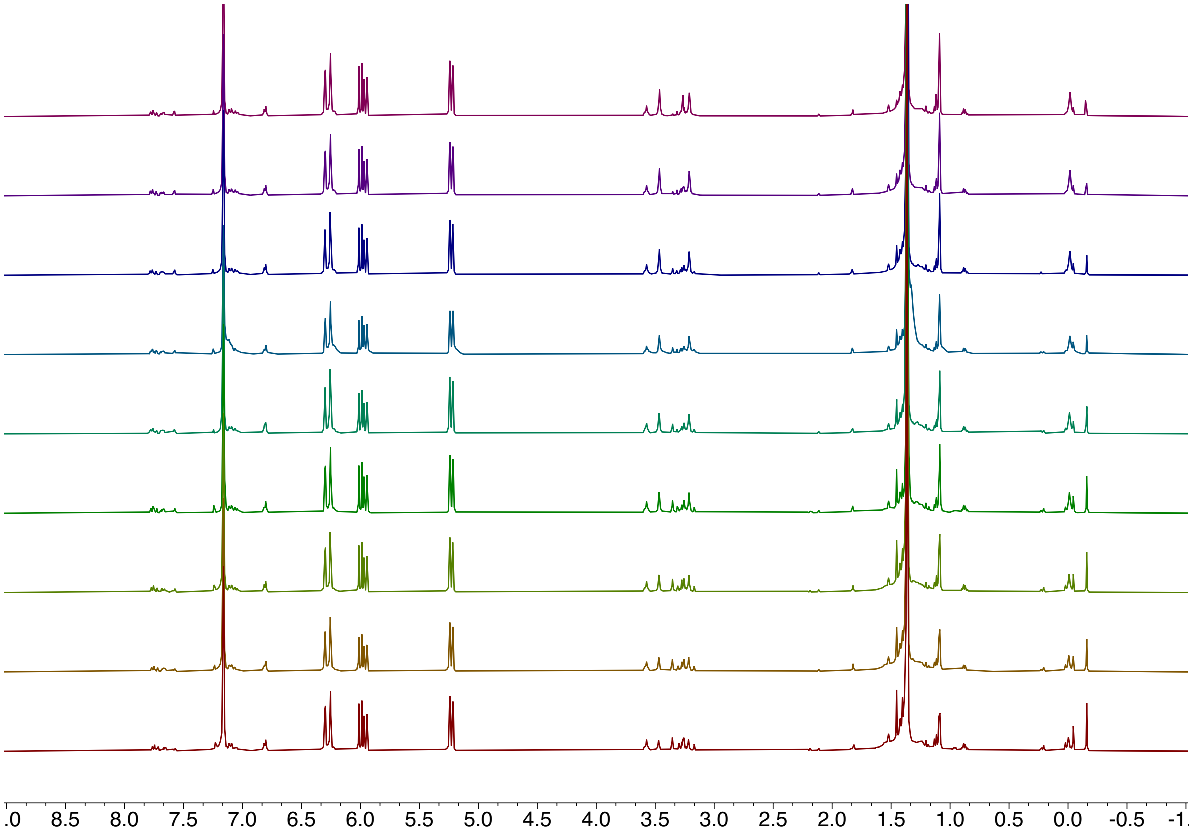

Figure S42. ^{1}H NMR monitoring of reaction of tBA with **1^{Me}** (Condition: [**1^{Me}**] = 0.0118 M, [tBA] = 0.177 M, solvent: C_6D_6, V(total) = 0.5 mL, T = 25 °C).

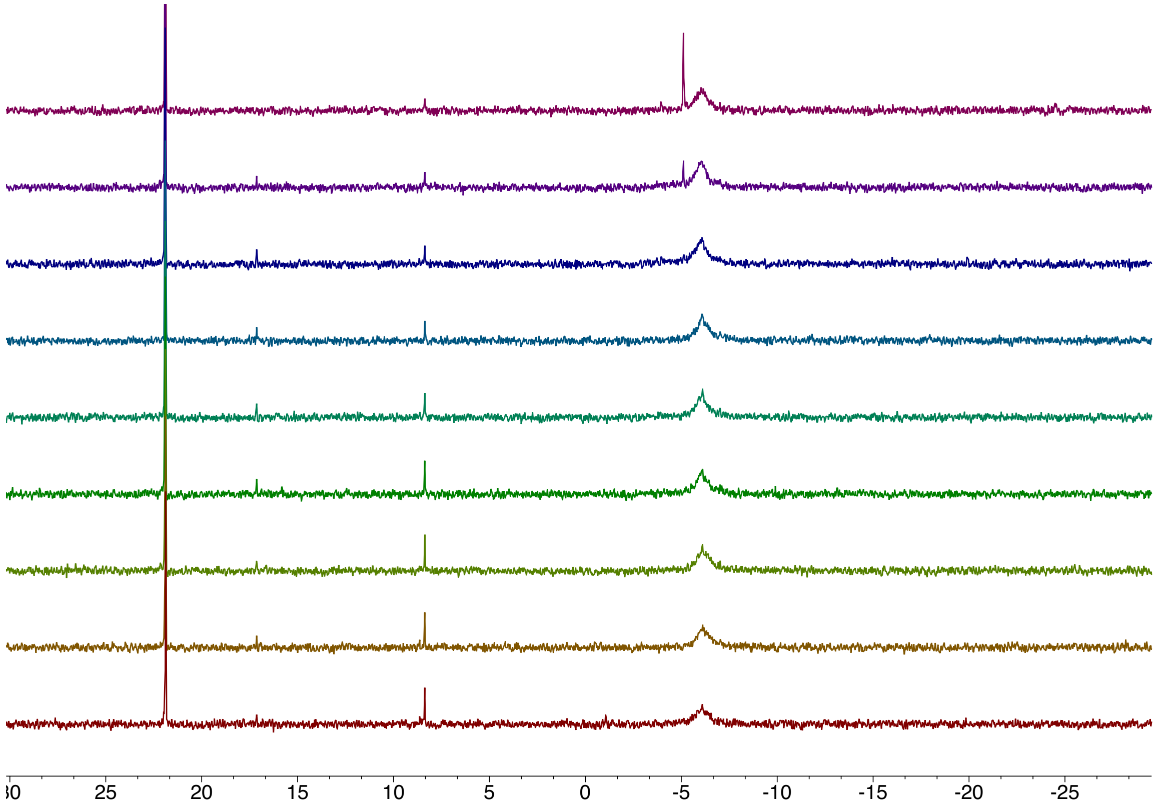

Figure S43. $^{31}P\{^1H\}$ NMR monitoring of reaction of tBA with **1^{Me}** (Condition: [**1^{Me}**] = 0.0118 M, [tBA] = 0.177 M, solvent: C_6D_6, V(total) = 0.5 mL, T = 25 °C. An external standard, $Ph_3PMe^+Br^-$, was added in a sealed capillary).

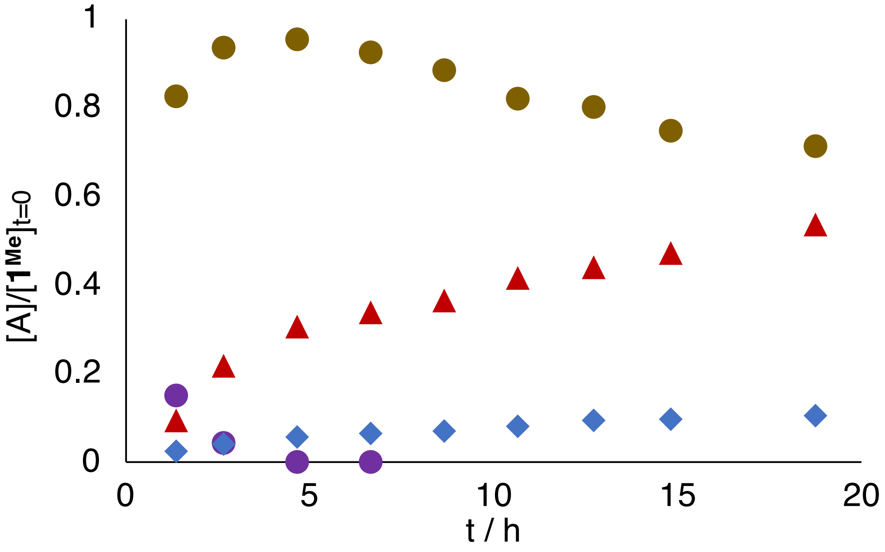

Figure S44. A kinetic profile of tBA insertion and subsequent reactions with **1^{Me}**. (Purple: **1^{Me}** (**Ni0**), brown: tBA inserted products, red: the internal olefin, blue: new species assigned to Ar_3PR^+ species based on literature.[100-101] Note: differentiation of the first insertion product, **2$^{Me\text{-}tBu}$** and subsequent insertion product, **4$^{Me\text{-}tBu}$**, is challenging and thus only the sum of them are shown as brown circles).

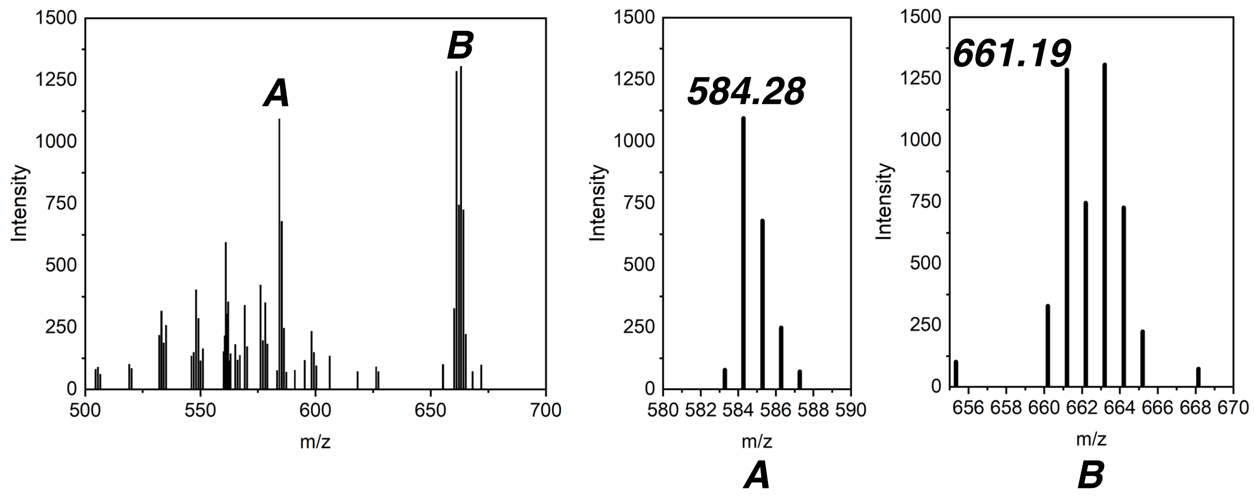

Figure S45. MALDI-TOF analysis of reaction of tBA and **1^{Me}**, indicating the presence of two major species with masses of 584 and 661. (Condition: [**1^{Me}**] = 0.0118 M, [tBA] = 0.177 M, solvent: C_6D_6, V(total) = 0.5 mL, T = 25 °C, t = 20 h. After reaction, the mixture was diluted with methanol prior to MALDI-TOF measurements).

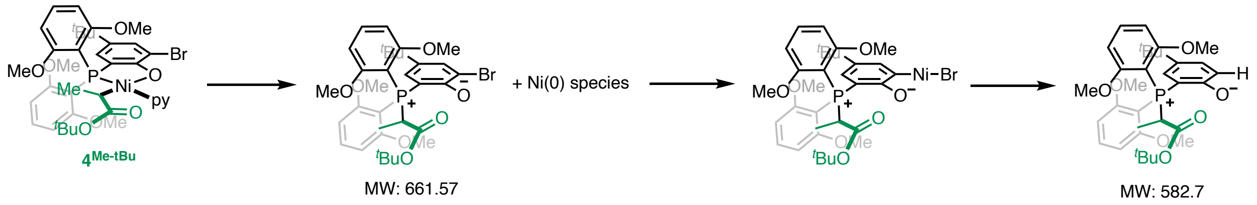

Figure S46. Possible pathways generating two species with masses of 583 and 661. The zwitterion may accept one proton under MALDI-TOF conditions and causes mass difference of 1.

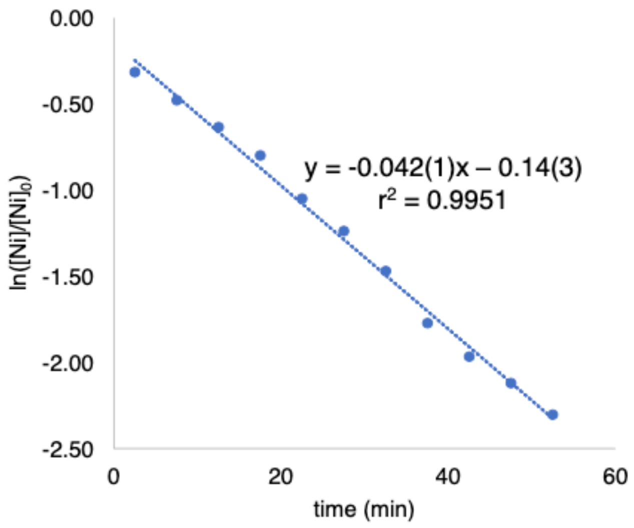

Figure S47. Log plot of relative concentration of **1**^{Me} vs time. (Condition: [**1**^{Me}] = 0.0118 M, [py] = 0.0236 M, [tBA] = 0.59 M, solvent: C_6D_6, V(total) = 0.5 mL, T = 40 °C).

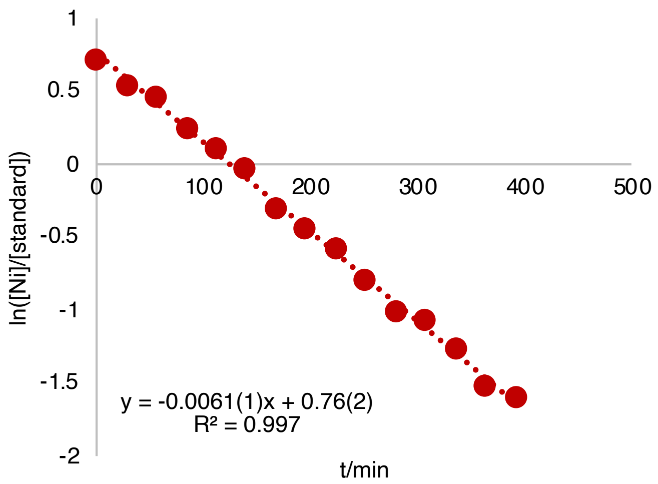

Figure S48. Log plot of relative concentration of **1**^{Ph} vs time. (Condition: [**1**^{Ph}] = 0.0118 M, [py] = 0.0236 M, [tBA] = 0.59 M, solvent: C_6D_6, V(total) = 0.5 mL, T = 40 °C).

8. Quantitative Kinetic Studies of Acrylate-Induced Reactions

Procedures: 0.0059 mmol of Ni catalyst prepared using the above procedure was dissolved in a C_6D_6 solution of pyridine (2 equiv.) and transferred to a J-Young tube. The solution was frozen in the coldwell pre-cooled by a liquid nitrogen bath, and a set amount of methyl acrylate (MA) was added via syringe (Total volume=0.50 ml). The resulting mixture was warmed up to thawing temperature and shaken vigorously prior to transferring to pre-heated NMR probe for acquisition of spectra at 25 ºC. NMR monitoring of tBA insertion were performed by monitoring ^1H and $^{31}P\{^1H\}$ NMR spectra.

Note: An external standard, $Ph_3PMe^+Br^-$, was added in a sealed capillary. A large excess of tBA and a small amount of pyridine were added to make sure their concentrations remain similar during monitoring (*pseudo*-1st order conditions).

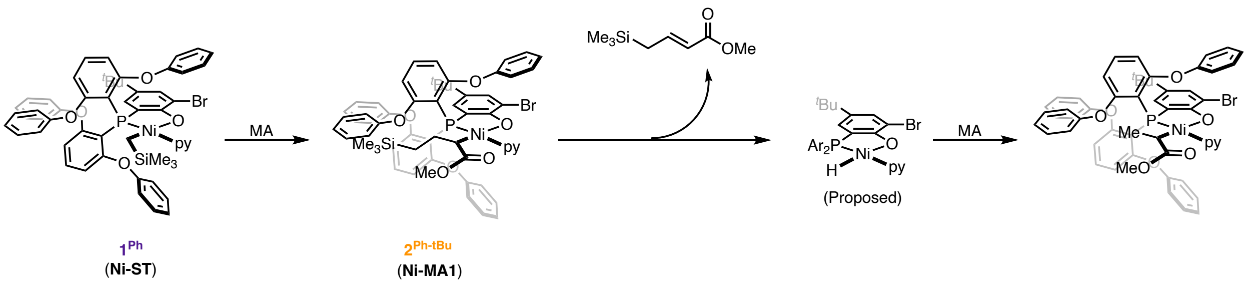

Original kinetic plots:

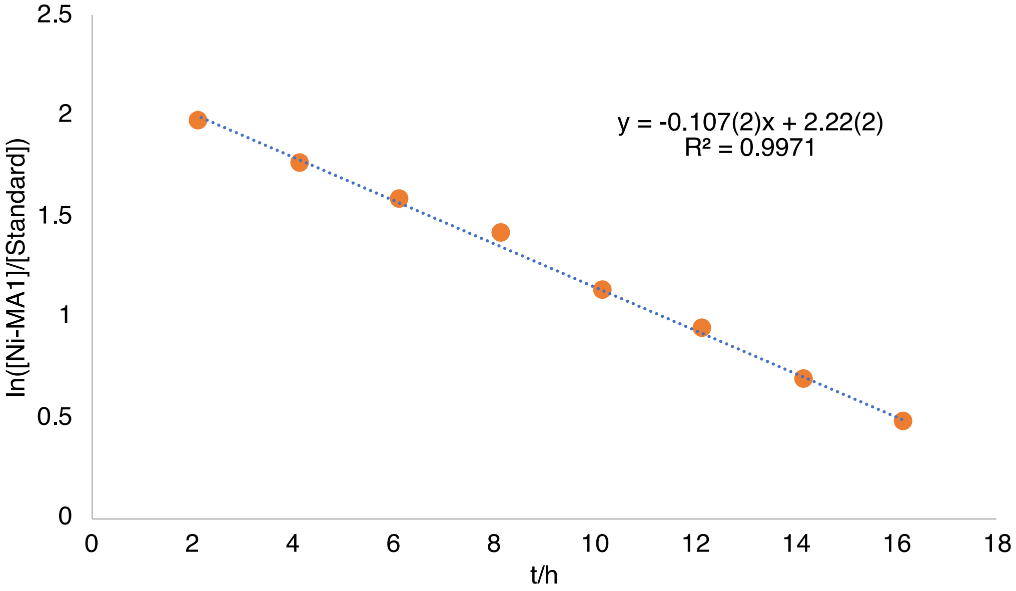

Figure S49. Log plot of relative concentration of **2**$^{\mathbf{Ph\text{-}tBu}}$ (Ni-MA1) vs time (Condition: [**1**$^{\mathbf{Ph}}$] = 0.0118 M, [py] = 0, [MA] = 0.177 M, solvent: C_6D_6, V(total) = 0.5 mL, T = 25 °C).

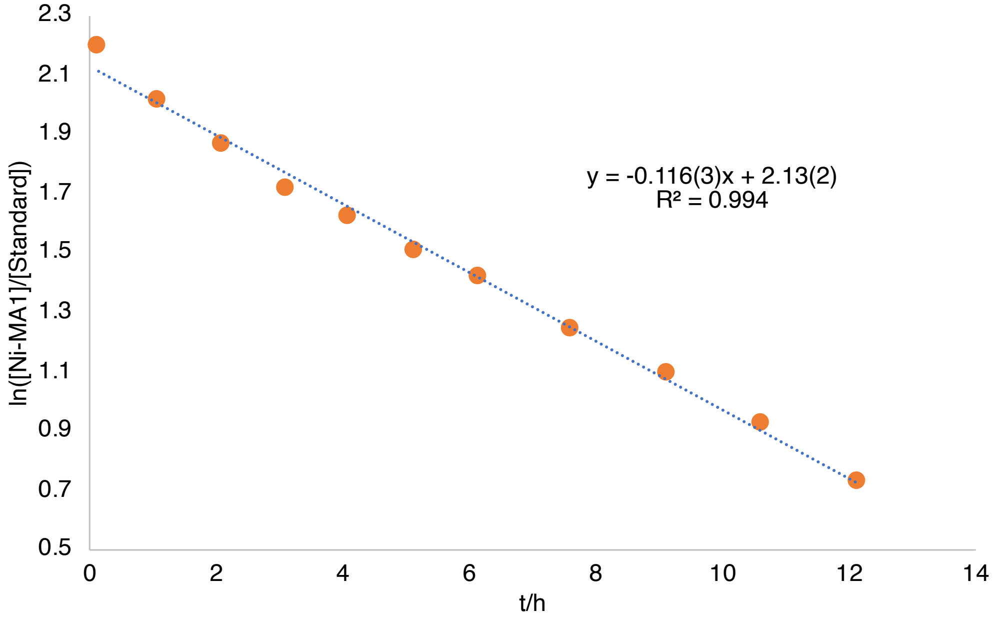

Figure S50. Log plot of relative concentration of **2**$^{\mathbf{Ph\text{-}tBu}}$ (Ni-MA1) vs time (Condition: [**1**$^{\mathbf{Ph}}$] = 0.0118 M, [py] = 0, [MA] = 0.59 M, solvent: C_6D_6, V(total) = 0.5 mL, T = 25 °C).

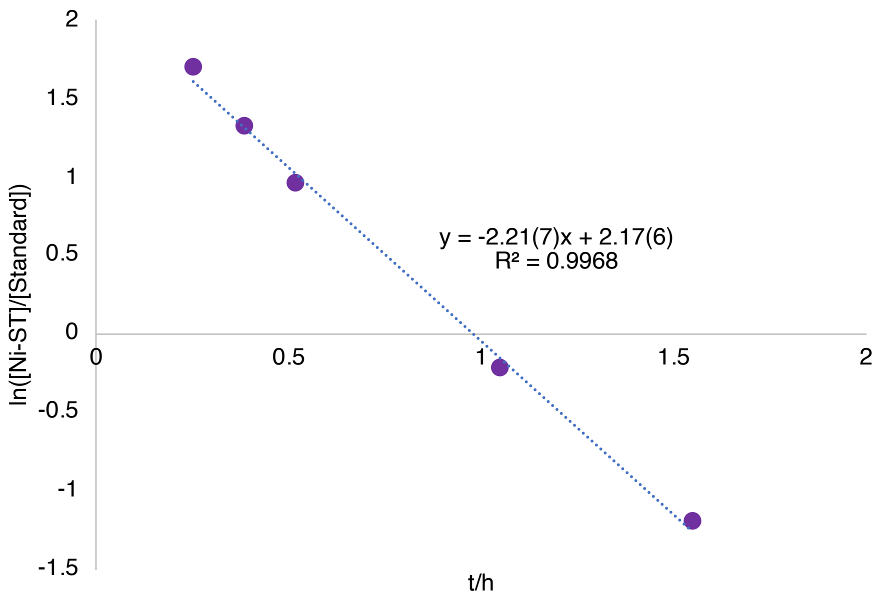

Figure S51. Log plot of relative concentration of **1**^{Ph} (Ni-ST) vs time (Condition: [**1**^{Ph}] = 0.0118 M, [py] = 0.0039 M, [MA] = 0.59 M, solvent: C_6D_6, V(total) = 0.5 mL, T = 25 °C).

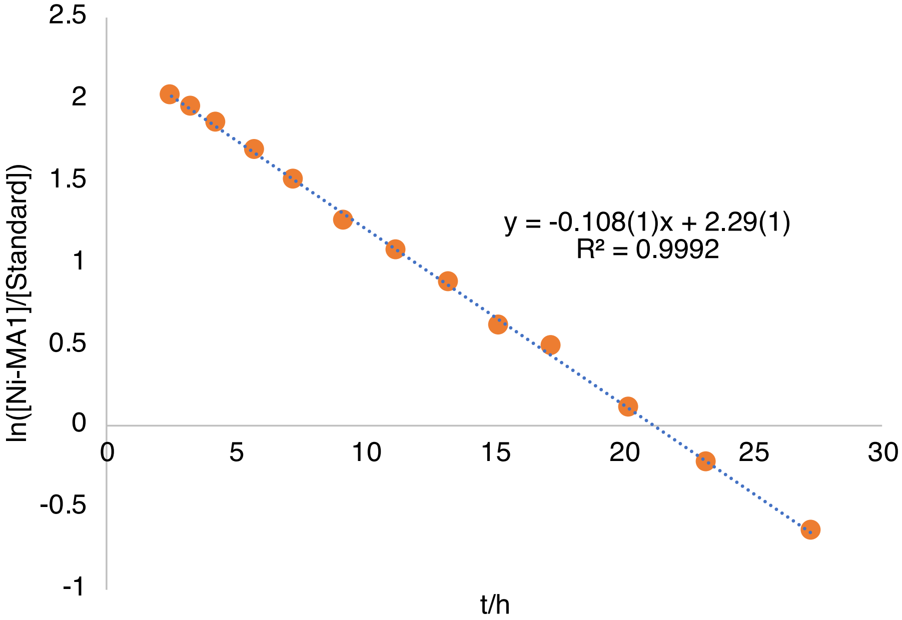

Figure S52. Log plot of relative concentration of **2**$^{Ph\text{-}tBu}$ (Ni-MA1) vs time (Condition: [**1**^{Ph}] = 0.0118 M, [py] = 0.0039 M, [MA] = 0.59 M, solvent: C_6D_6, V(total) = 0.5 mL, T = 25 °C).

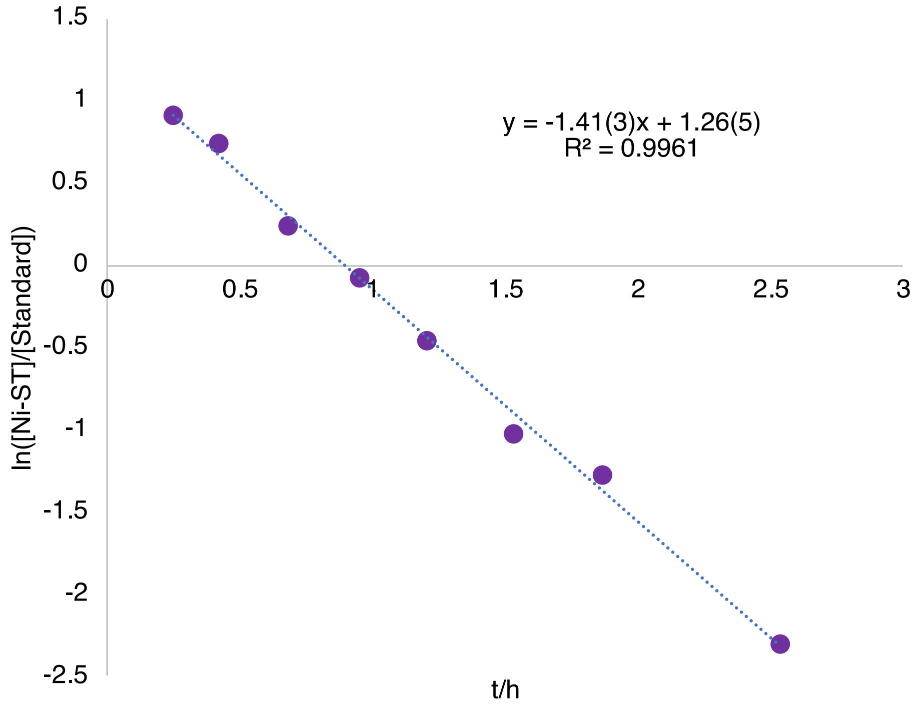

Figure S53. Log plot of relative concentration of **1^{Ph}** (Ni-ST) vs time (Condition: [**1^{Ph}**] = 0.0118 M, [py] = 0.0059 M, [MA] = 0.59 M, solvent: C_6D_6, V(total) = 0.5 mL, T = 25 °C).

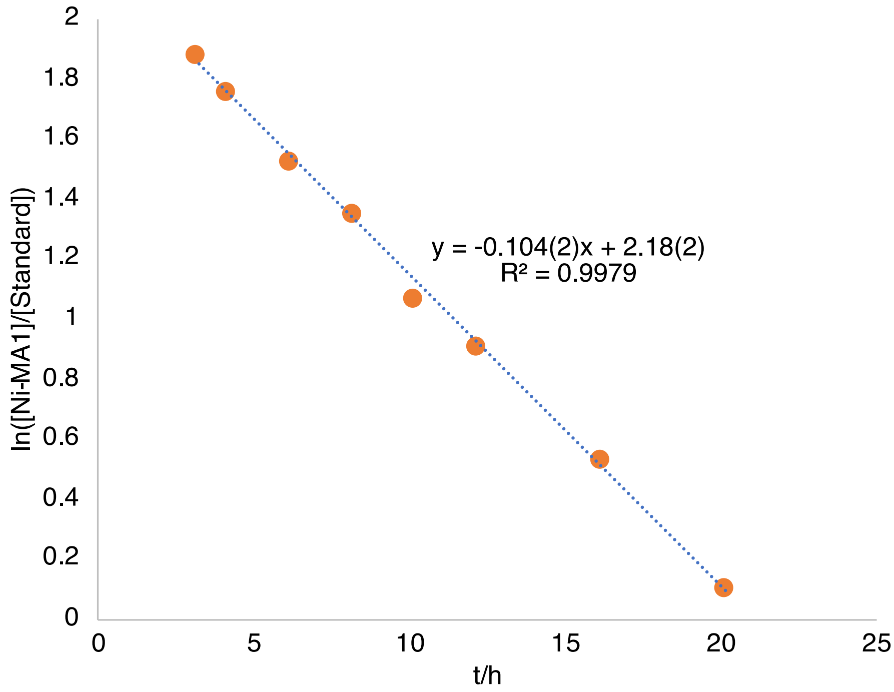

Figure S54. Log plot of relative concentration of of **2^{Ph-tBu}** (Ni-MA1) vs time (Condition: [**1^{Ph}**] = 0.0118 M, [py] = 0.0059 M, [MA] = 0.59 M, solvent: C_6D_6, V(total) = 0.5 mL, T = 25 °C).

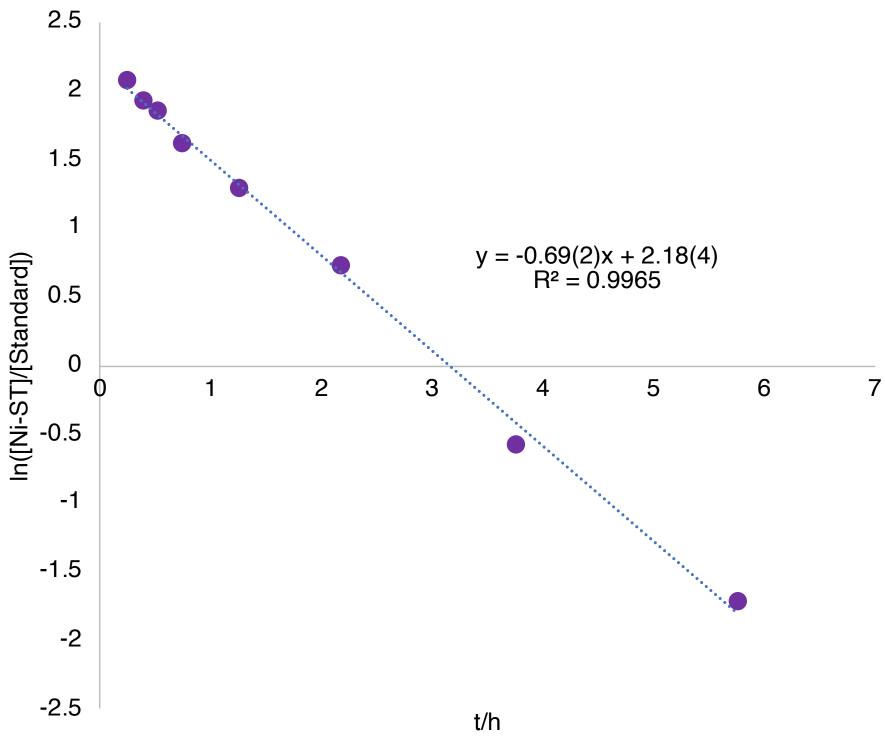

Figure S55. Log plot of relative concentration of **1**^{Ph} (Ni-ST) vs time (Condition: [**1**^{Ph}] = 0.0118 M, [py] = 0.0118 M, [MA] = 0.59 M, solvent: C_6D_6, V(total) = 0.5 mL, T = 25 °C).

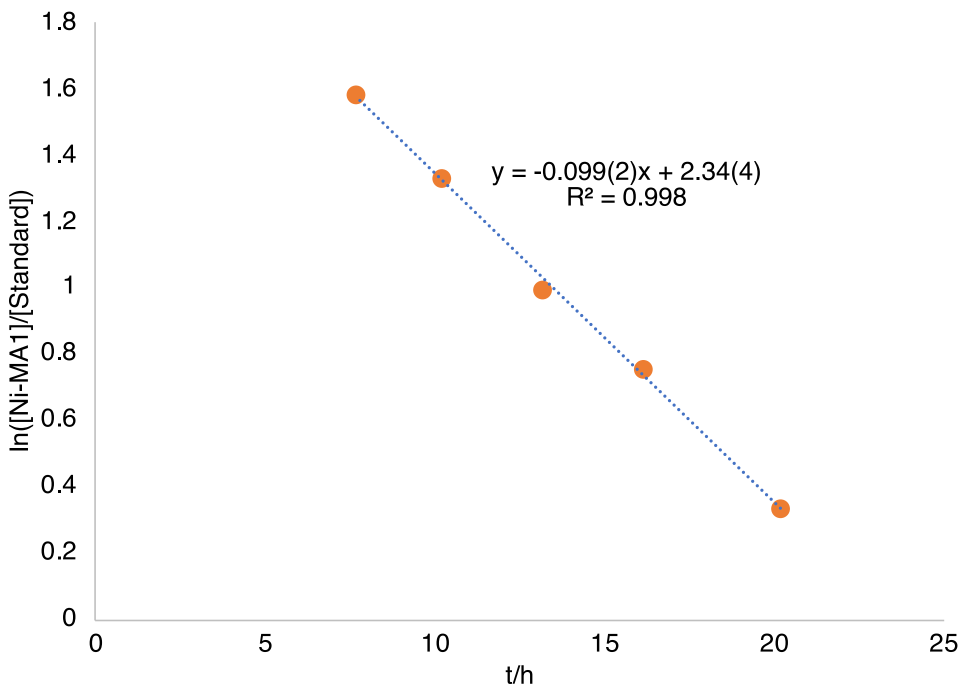

Figure S56. Log plot of relative concentration of **2**$^{Ph\text{-}tBu}$ (Ni-MA1) vs time (Condition: [**1**^{Ph}] = 0.0118 M, [py] = 0.0118 M, [MA] = 0.59 M, solvent: C_6D_6, V(total) = 0.5 mL, T = 25 °C).

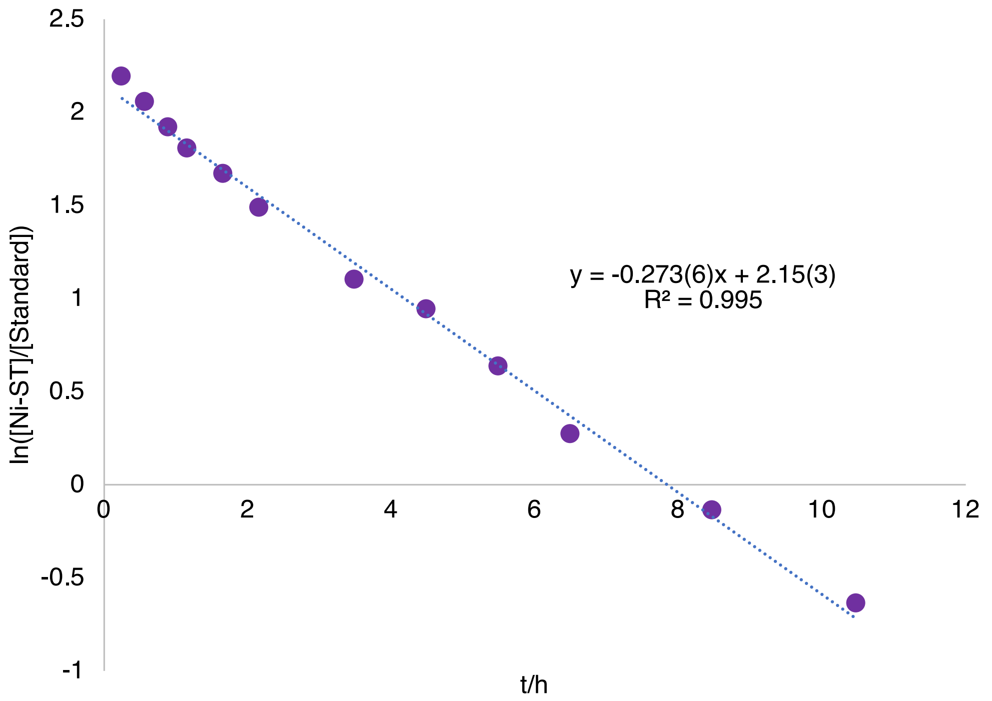

Figure S57. Log plot of relative concentration of **1**^{Ph} (Ni-ST) vs time (Condition: [**1**^{Ph}] = 0.0118 M, [py] = 0.0236 M, [MA] = 0.59 M, solvent: C_6D_6, V(total) = 0.5 mL, T = 25 °C).

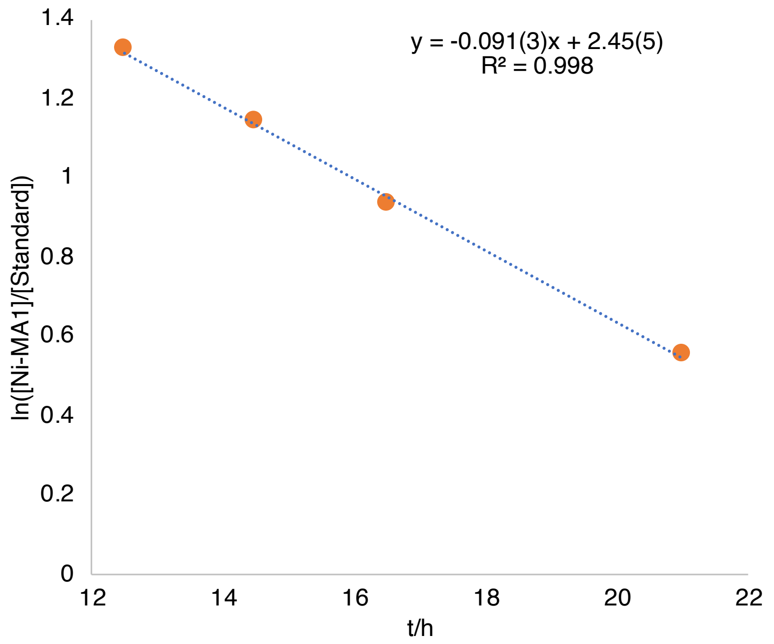

Figure S58. Log plot of relative concentration of **2**$^{Ph\text{-}tBu}$ (Ni-MA1) vs time (Condition: [**1**^{Ph}] = 0.0118 M, [py] = 0.0236 M, [MA] = 0.59 M, solvent: C_6D_6, V(total) = 0.5 mL, T = 25 °C).

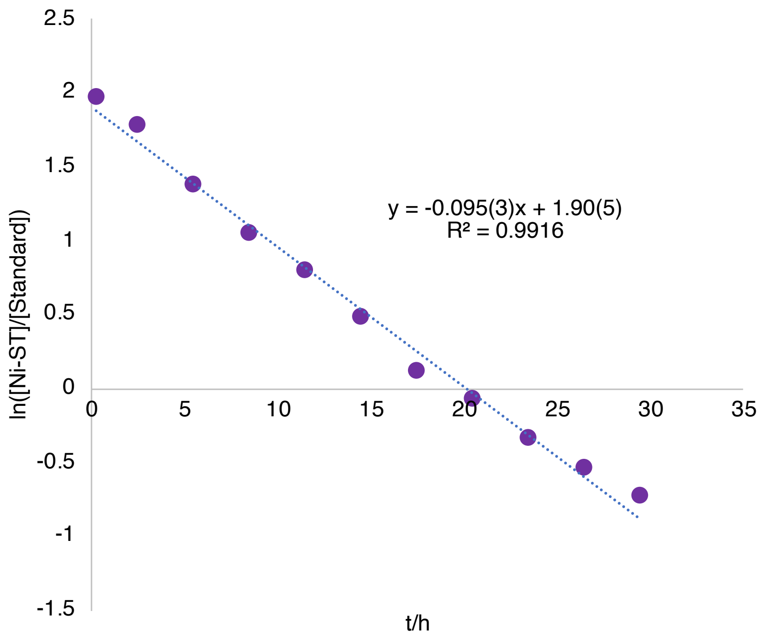

Figure S59. Log plot of relative concentration of **1**[Ph] (Ni-ST) vs time (Condition: [**1**[Ph]] = 0.0118 M, [py] = 0.059 M, [MA] = 0.59 M, solvent: C_6D_6, V(total) = 0.5 mL, T = 25 °C).

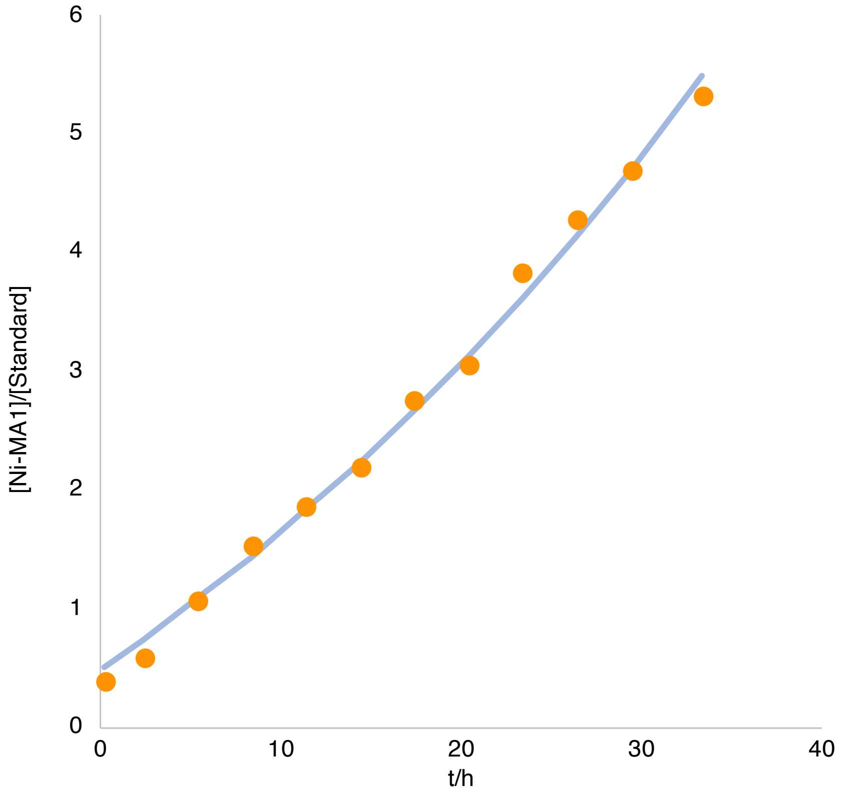

Figure S60. Plot of relative concentration of **2**[Ph-tBu] (Ni-MA1) vs time (Condition: [**1**[Ph]] = 0.0118 M, [py] = 0.059 M, [MA] = 0.59 M, solvent: C_6D_6, V(total) = 0.5 mL, T = 25 °C. Red spots: experimental data, blue line: fitted curve.)

- p=-0.02, SSR=0.153, k_2=0.0742 (See below for methods)

Methods for Figure S60. Pseudo-1st order rate constant of β-H elimination can be obtained based on changes of [**Ni-ST**] and [**Ni-MA1**] over time, which is shown below.

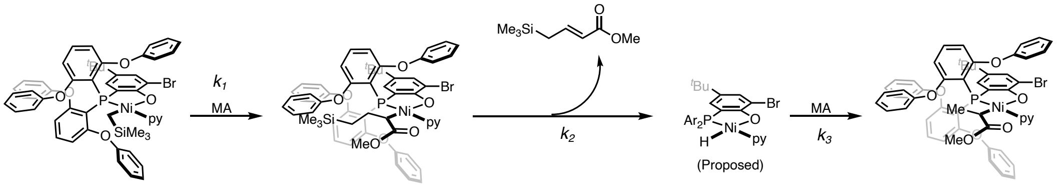

Ni-ST
A

Ni-MA1
B

$$\frac{d[A]}{dt} = -k_1 \cdot [A] \quad \textbf{(i)}$$

$$[A] = [A]_0 \cdot e^{-k_1 t} \quad \textbf{(ii)}$$

$$\frac{d[B]}{dt} = k_1 \cdot [A] - k_2 \cdot [B] \quad \textbf{(iii)}$$

Based on (ii) and (iii), $k_2 \cdot [B] + \frac{d[B]}{dt} = k_1 \cdot [A]_0 \cdot e^{-k_1 t}$ **(iv) ->**

$$k_2 \cdot e^{k_2 t} \cdot [B] + e^{k_2 t} \cdot \frac{d[B]}{dt} = k_1 \cdot [A]_0 \cdot e^{k_2 t} \cdot e^{-k_1 t} \quad \textbf{(v) ->}$$

$$\frac{d}{dt}(e^{k_2 t} \cdot [B]) = k_1 \cdot [A]_0 \cdot e^{(k_2 - k_1)t} \quad \textbf{(vi) ->}$$

$$e^{k_2 t} \cdot [B] = \frac{k_1}{k_2 - k_1} \cdot [A]_0 \cdot e^{(k_2 - k_1)t} + \mathrm{C} \quad \textbf{(vii) ->}$$

$$[B] = \frac{k_1}{k_2 - k_1} \cdot [A]_0 \cdot e^{-k_1 t} + \mathrm{C} \cdot e^{k_2 t} \quad \textbf{(viii)}$$

t=0, [B]=0, ∴ C=$-\frac{k_1}{k_2 - k_1} \cdot [A]_0$ **(ix)**

Based on (viii) and (ix), $[B] = \frac{k_1}{k_2 - k_1} \cdot [A]_0 \cdot (e^{-k_1 t} - e^{k_2 t})$ **(x)**

(viii)/(iii) -> $\frac{[B]}{[A]} = \frac{k_1}{k_2 - k_1} \cdot (1 - e^{-(k_2 - k_1)t})$ **(xi)**

If p=$k_2 - k_1$, $\frac{[B]}{[A]} = \frac{k_1}{p} \cdot (1 - e^{-pt})$ **(xii)**

[B]/[A] can be obtained from spectra, thereby p is solved via minimizing the difference of calculated curve (y axis: [B]/[A], x axis: time) and curve generated from exp in excel ("solver" add-on).

Analysis of β-H elimination

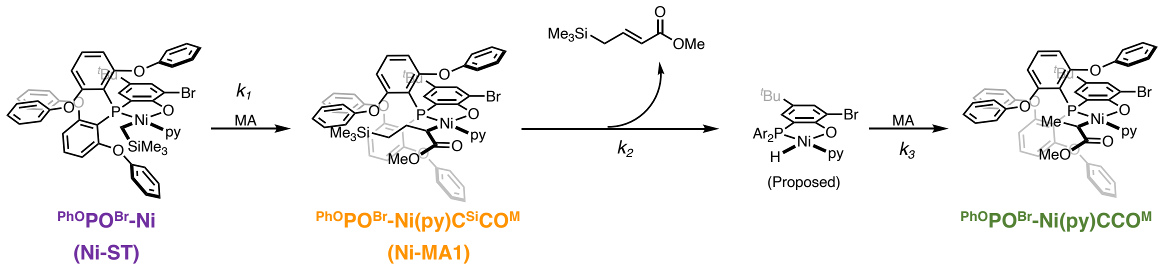

Table S4. *Pseudo*-1st order constants of the β-H elimination step (k_2) under different pyridine and acrylate concentrations.

Entry	[MA]/[Ni]	[py]/[Ni]	$k_{2\text{-obs}}$ (elimination)/h
1	15	0	0.107(2)
2	50	0	0.116(3)
3	50	0.33	0.108(1)
4	50	0.5	0.104(2)
5	50	1	0.099(2)
6	50	2	0.091(3)
7	50	5	0.0742

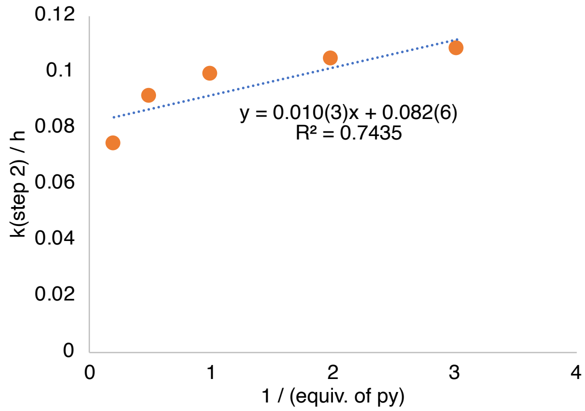

Figure S61. Plot of *pseudo*-1st order rate constant of β-H elimination after MA insertion vs 1 / (equivalents of pyridine added) (Conditions: [**PhOPOBr-Ni**] = 0.0118 M, [py] = 0.0039-0.059 M, [MA] = 0.59 M, solvent: C_6D_6, V(total) = 0.5 mL, T = 25 °C).

As shown in the figure, a simple linear relationship in NOT observed between *pseudo*-1st order rate constant of the β-H elimination step (k_2) and equivalents of pyridine. This suggests a kinetic profile more complex than the pathway featuring a fast dissociative pre-equilibrium (See section S8 for rationale and other possibilities).

9. Discussion of pathways of β-H elimination

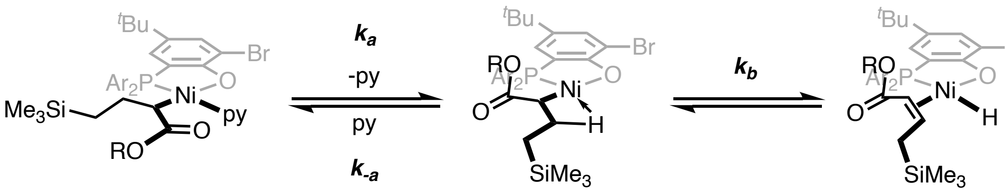

Case 1: Fast dissociation equilibrium (but K_a<<1, step a) followed by slow β-H transfer (step b)

$$\text{Rate} = \frac{k_b K_a}{[py]}\,[\text{Ni-CCO-py}]$$

$$k(\text{elimination}) = \frac{k_b K_a}{[py]}$$

This scenario is not consistent with Figure S61, as a simple linear relationship in NOT observed between pseudo-1st order rate constant of the β-H elimination step (k2) and equivalents of pyridine, and not consistent with Figure 3h, as the line is expected to cross the origin.

Case 2: Slow exchange (concerted mechanism, step a) followed by fast β-H transfer (step b)

$$\text{Rate} = k_a \frac{k_b}{k_{-a}[py]+k_b}\,[\text{Ni-CCO-py}]$$

$$k(\text{elimination}) = k_a \frac{k_b}{k_{-a}[py]+k_b},$$

$$[py]=0,\ k(\text{elimination}) = k_a$$

$$1/k(\text{elimination}) = \frac{k_{-a}[py]+k_2}{k_a k_b}$$

This scenario is consistent with Figure 3h.

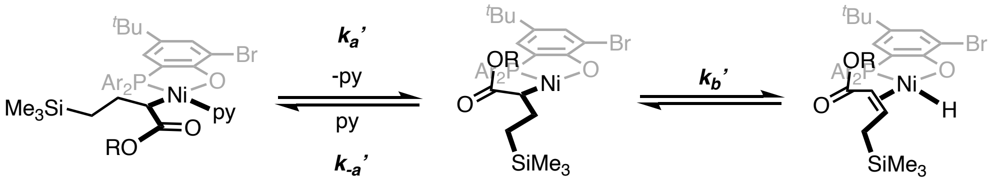

Case 3: Dissociative mechanism – slow pyridine dissociation followed by fast β-H transfer (step 2)

$$\text{Rate} = k_a' \frac{k_{b'}}{k_{-a'}[\text{py}]+k_{b'}} [\text{Ni-CCO-py}]$$

Similar to case 2, this case is also consistent with Figure 3h.

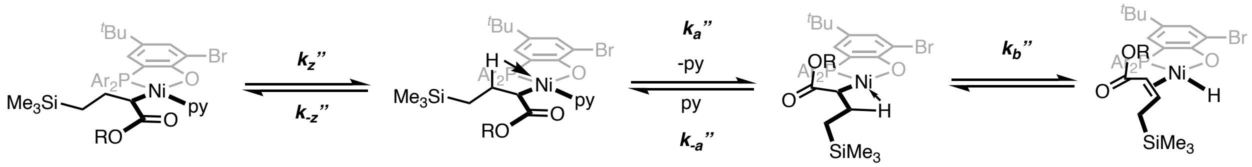

Case 4: Associative mechanism – slow coordination of fifth ligand followed by fast subsequent steps.

Rate = K [Ni-CCO-py] (K is a constant independent of pyridine concentration)

This scenario is not consistent with Table S4.

Case 5: Associative mechanism – fast coordination of fifth ligand follow by slow pyridine dissociation.

$$\text{Rate} = K_z'' k_a'' \frac{k_{b''}}{k_{-a''}[\text{py}]+k_{b''}} [\text{Ni-CCO-py}]$$

Similar as case 2, this case is also consistent with Figure 3h.

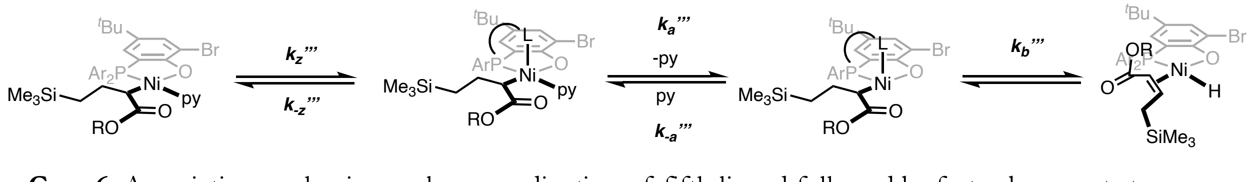

Case 6: Associative mechanism – slow coordination of fifth ligand followed by fast subsequent steps

Rate = K [Ni-CCO-py] (K is a constant independent of pyridine concentration)

This scenario is not consistent with Table S4.

Case 7: Associative mechanism – fast coordination of fifth ligand follow by slow pyridine dissociation.

$$\text{Rate} = K_z'''k_a'''\frac{k_b'''}{k_{-a}'''[\text{py}]+k_b'''}[\text{Ni-CCO-py}]$$

Similar as case 2, this case is also consistent with Figure 3h.

Overall, the linear relationship revealed by Figure 3h is consistent with case 2,3,4,5,7 in which pyridine dissociation is slow. This scenario implies that pyridine plays an important role in β-H elimination.

10. Crystallographic Information

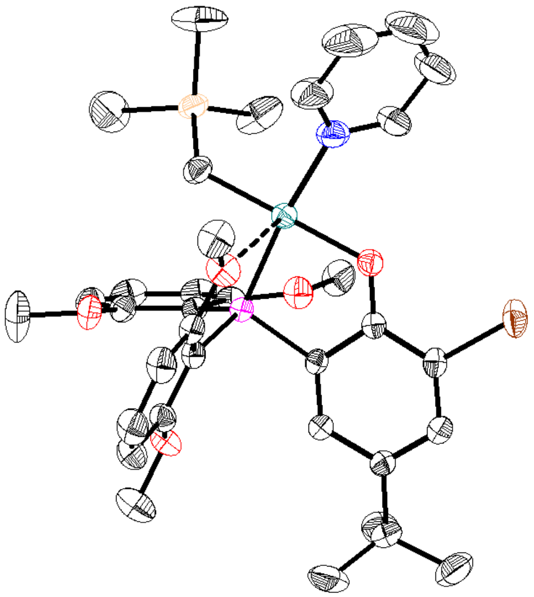

Figure S62. Solid-State Structure of $\mathbf{1^{Me}}$ (Green: Ni, Pink: P, Blue: N, Red: O, orange: Si, black: C). Ellipsoids are show at the 50% probability level. Hydrogen atoms and solvent molecules excluded for clarity.

Special Refinement Details for $\mathbf{1^{Me}}$: Complex $\mathbf{1^{Me}}$ crystalizes in a P-1 space group with two full molecules in the asymmetric unit, as well as 3/8 THF molecule. A disorder was present in the 3/8 THF molecule and could not be modelled. The solvent mask (Olex® implementation of BYPASS/SQUEEZE) was used to suppress two sections of electron density likely corresponding to 1/4 THF molecule and 1/8 THF molecule. The void was calculated to be near 51 electrons per unit cell, which would be close to 3/8 THF molecule per asymmetric unit (Z=4).

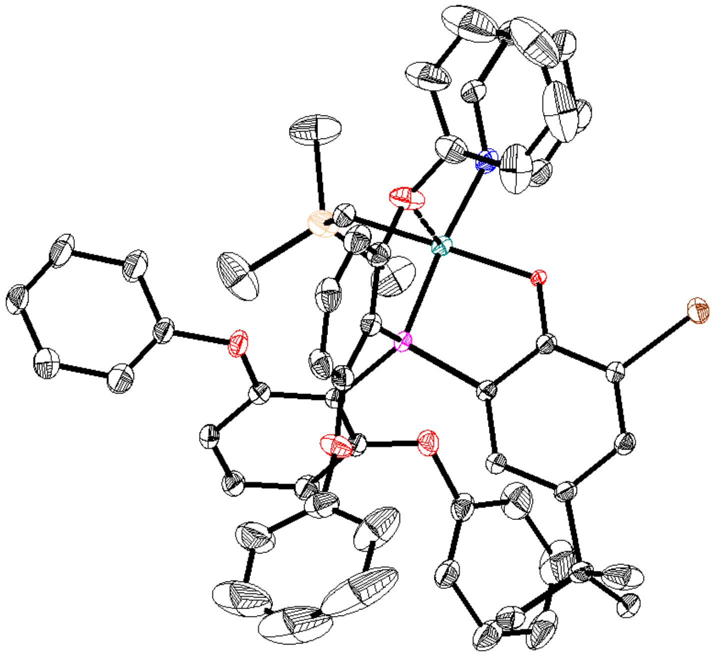

Figure S63. Solid-State Structure of $\mathbf{1^{Ph}}$ (Green: Ni, Pink: P, Blue: N, Red: O, orange: Si, black: C). Ellipsoids are show at the 50% probability level. Hydrogen atoms and solvent molecules excluded for clarity.

Special Refinement Details for $\mathbf{1^{Ph}}$: Complex $\mathbf{1^{Ph}}$ crystalizes in a $P2_1/n$ space group with one full molecule in the asymmetric unit, as well as one toluene molecule. A disorder was present in the one toluene molecule and could not be modelled. The solvent mask (Olex® implementation of BYPASS/SQUEEZE) was used to suppress one section of electron density likely corresponding to this one toluene molecule. The void was calculated to be near 184 electrons per unit cell, which would be close to one toluene molecule per asymmetric unit (Z=4).

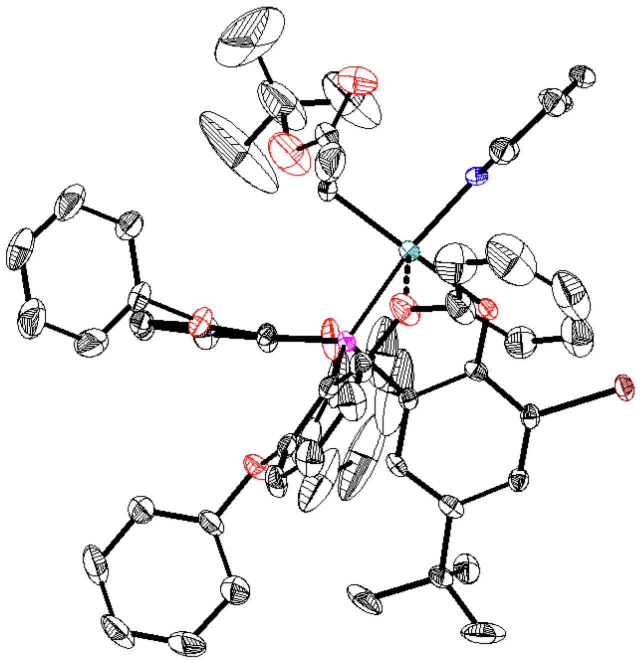

Figure S64. Solid-State Structure of $\mathbf{4^{Ph\text{-}tBu}}$ (Green: Ni, Pink: P, Blue: N, Red: O, orange: Si, black: C). Ellipsoids are show at the 50% probability level. Hydrogen atoms and solvent molecules excluded for clarity.

Special Refinement Details for $\mathbf{4^{Ph\text{-}tBu}}$: Complex $\mathbf{4^{Ph\text{-}tBu}}$ crystalizes in a $P2_1/n$ space group with one full molecule in the asymmetric unit, as well as half toluene molecule. One methyl group on t-butoxy moiety is modelled with two-site disorder with half occupancies for each. A disorder was present in the one and a half pentane molecules and could not be modelled. The solvent mask (Olex® implementation of BYPASS/SQUEEZE) was used to suppress one section of electron density likely corresponding to this half toluene molecule. The void was calculated to be near 108 electrons per unit cell, which would be close to half toluene molecule per asymmetric unit (Z=4).

Crystallographic Information

Table S5. Crystal and refinement data for complexes **1**$^{\mathbf{Me}}$, **1**$^{\mathbf{Ph}}$, and **4**$^{\mathbf{Ph\text{-}tBu}}$.

	**1**$^{\mathbf{Me}}$	**1**$^{\mathbf{Ph}}$	**4**$^{\mathbf{Ph\text{-}tBu}}$
Empirical formula	$C_{35}H_{45}BrNNiO_5PSi$	$C_{55}H_{52}BrNNiO_5PSi$	$C_{58}H_{55}BrNiO_7P$
Formula weight	774.67	1089.82	1110.15
Temperature/K	100 K	100 K	100 K
Crystal system	Triclinic	Monoclinic	Monoclinic
Space group	P-1	$P2_1/n$	$P2_1/n$
a/Å	13.161(3)	13.733(2)	13.546(13)
b/Å	15.276(3)	17.573(2)	17.949(14)
c/Å	21.447(5)	22.824(3)	22.94(3)
α/°	102.207(10)	90	90
β/°	101.799(18)	103.207(12)	105.05(4)
γ/°	99.382(18)	90	90
Volume/Å^3	4028.4(16)	5363(1)	5386(9)
Z	4	4	4
ρ_{calc}g/cm^3	1.277	1.350	1.369
μ/mm^{-1}	1.581	1.209	1.188
F(000)	1613	2280	2317
Radiation	MoKα (λ = 0.71073)	MoKα (λ = 0.71073)	MoKα (λ = 0.71073)
Reflections collected	128856	175043	75731
Independent reflections	26181	16282	16453
Goodness-of-fit on F^2	1.039	1.073	0.941
Final R indexes	R_1 = 4.00 %	R_1 = 4.02 %	R_1 = 10.31 %
[I>=2σ (I)]	R_2 = 10.00%	R_2 = 11.40 %	R_2 = 22.97 %

11. NMR Spectra of Ligands and Organometallic Complexes

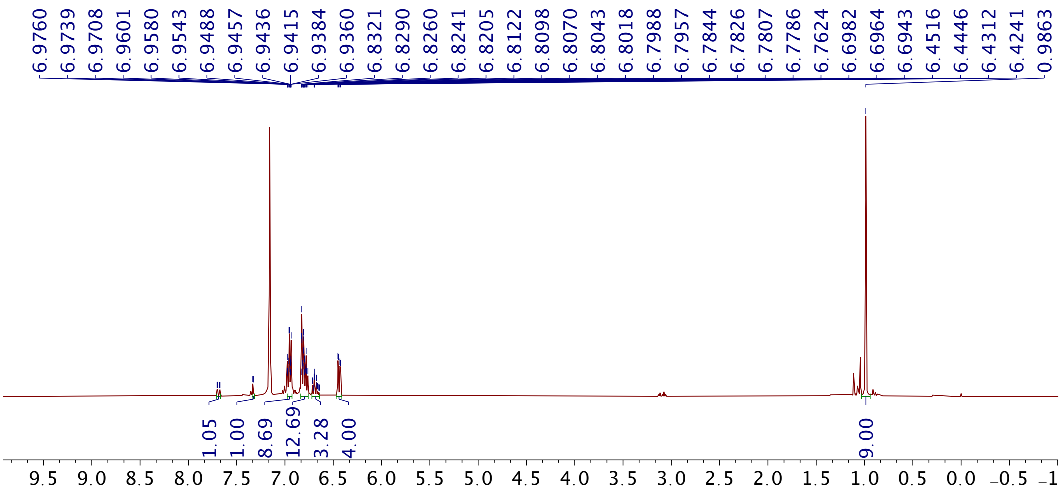

Figure S65. ^{1}H NMR spectrum of **^{PhO}PO^{Br}H** in C_6D_6.

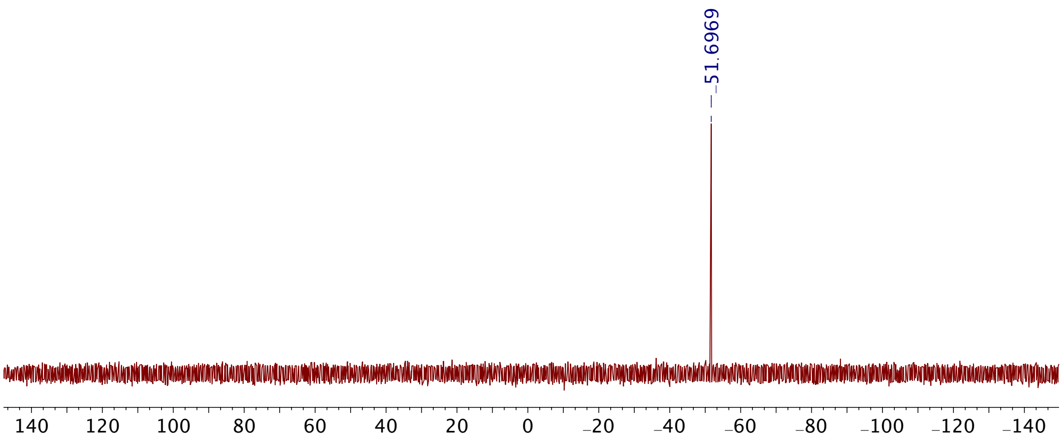

Figure S66. ^{31}P{^{1}H} NMR spectrum of **^{PhO}PO^{Br}H** in in C_6D_6.

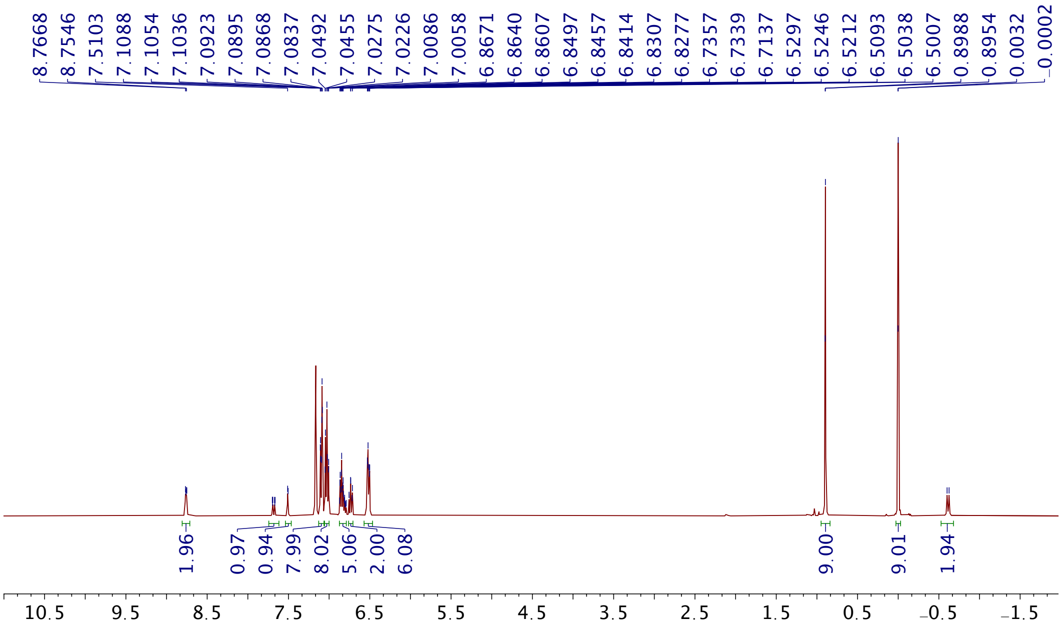

Figure S67. ^{1}H NMR spectrum of **1^{Ph}** in C_6D_6.

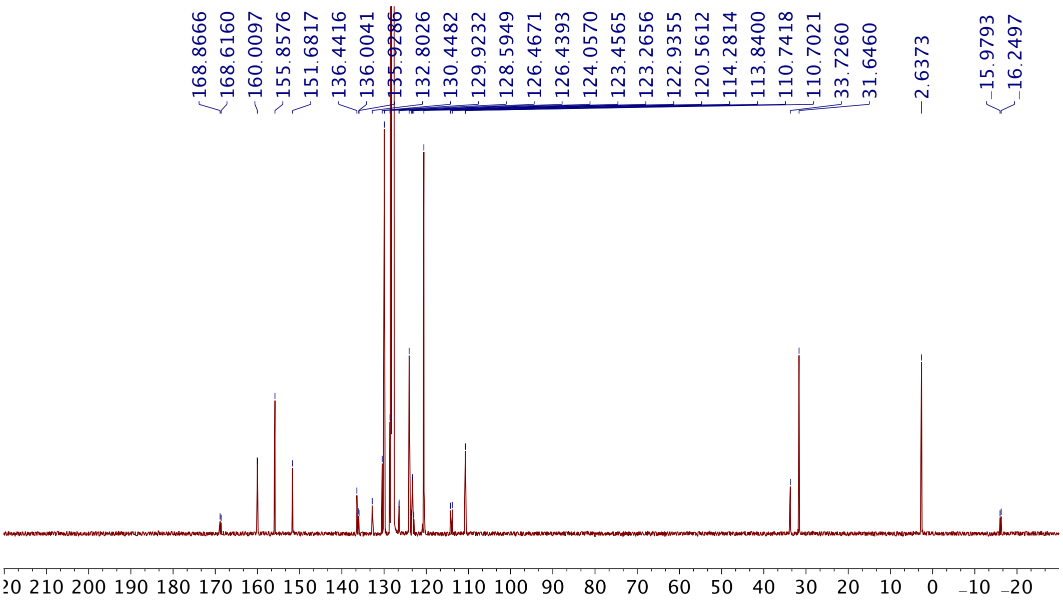

Figure S68. ^{13}C{^{1}H} NMR spectrum of **1^{Ph}** in C_6D_6.

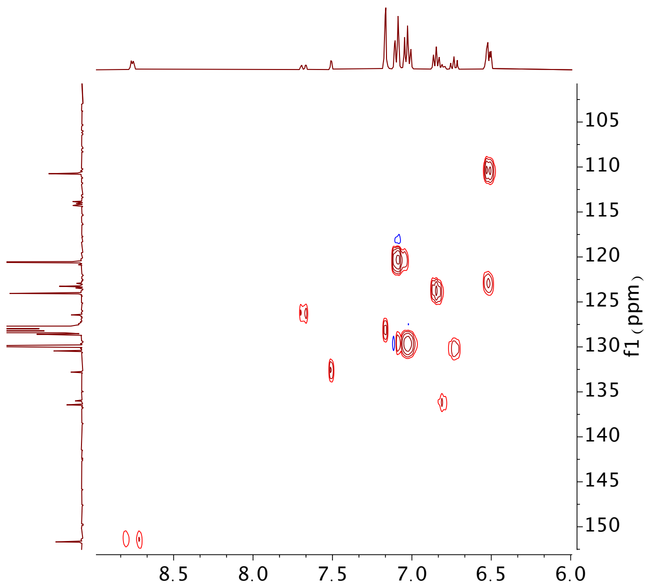

Figure S69. ^{1}H-$^{13}C\{^{1}H\}$ HSQC NMR spectrum of **1**$^{\mathbf{Ph}}$ in C_6D_6.

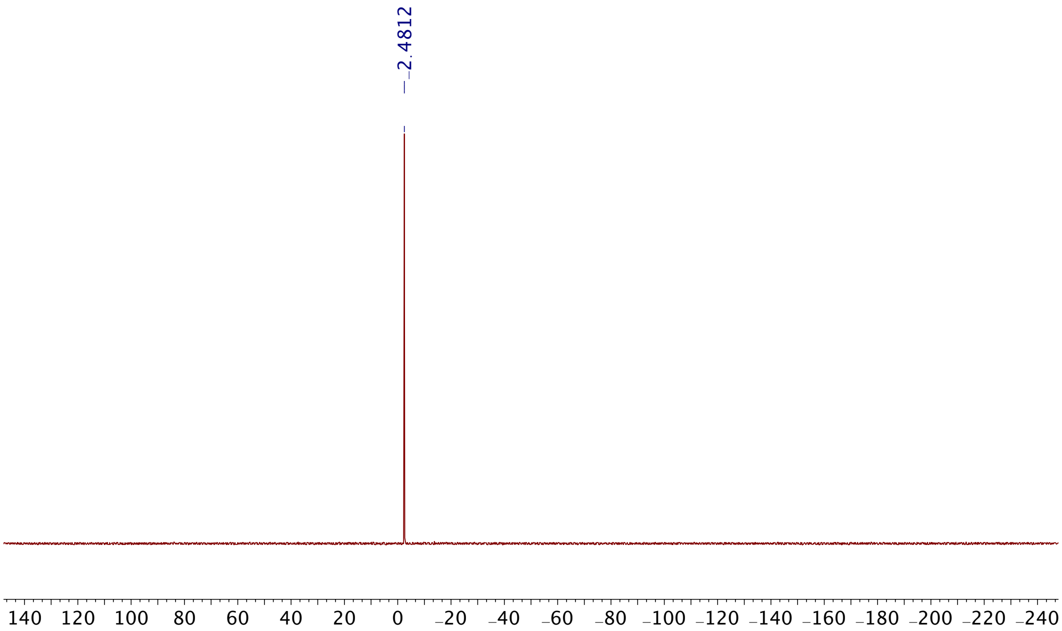

Figure S70. $^{31}P\{^{1}H\}$ NMR spectrum of **1**$^{\mathbf{Ph}}$ in in C_6D_6.

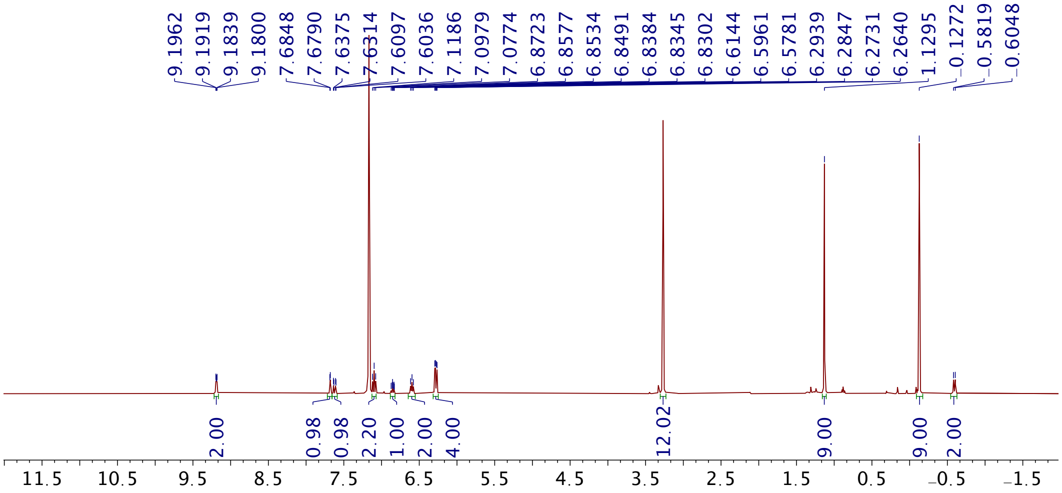

Figure S71. ^{1}H NMR spectrum of **1^{Me}** in C_6D_6.

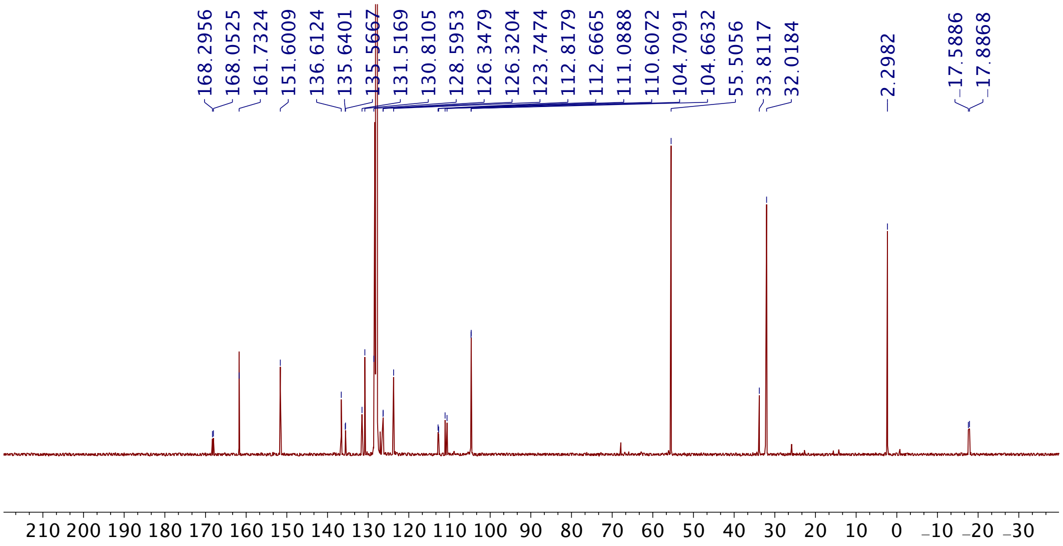

Figure S72. $^{13}C\{^{1}H\}$ NMR spectrum of **1^{Me}** in C_6D_6.

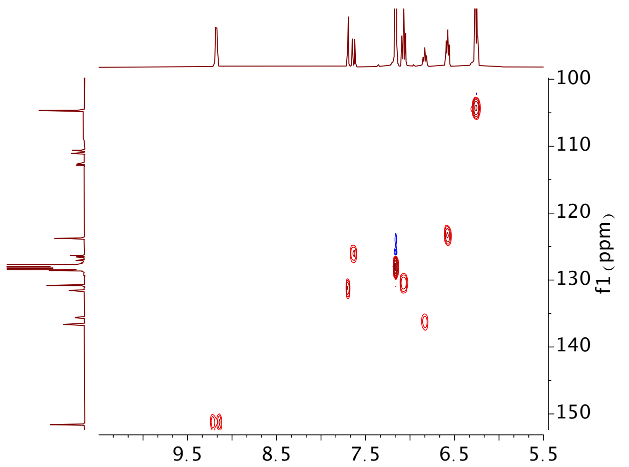

Figure S73. ^{1}H-$^{13}C\{^{1}H\}$ HSQC NMR spectrum of $\mathbf{1^{Me}}$ in C_6D_6.

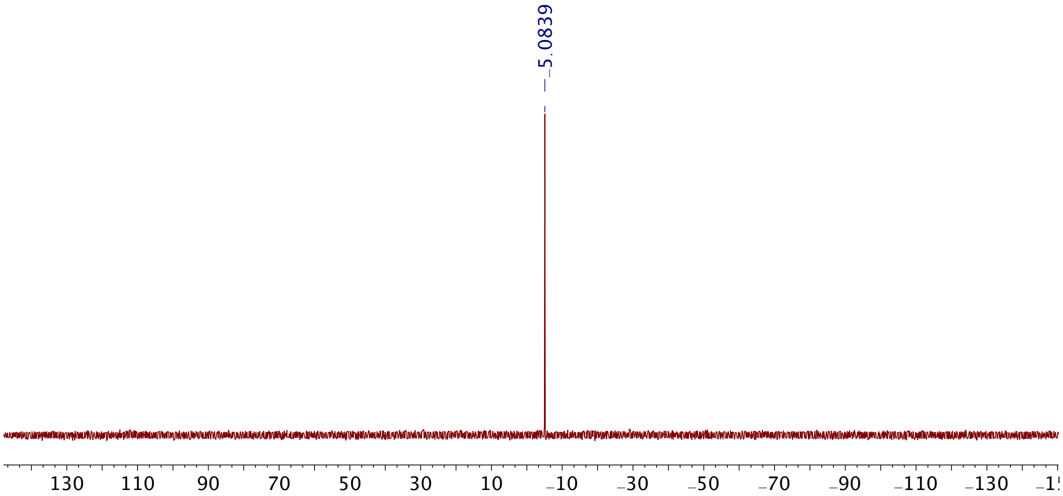

Figure S74. $^{31}P\{^{1}H\}$ NMR spectrum of $\mathbf{1^{Me}}$ in in C_6D_6.

References

(1) Waltman, A. W.; Younkin, T. R.; Grubbs, R. H., Insights into the deactivation of neutral nickel ethylene polymerization catalysts in the presence of functionalized olefins. *Organometallics* **2004,** *23* (22), 5121-5123.
(2) Low, C. H.; Rosenberg, J. N.; Lopez, M. A.; Agapie, T., Oxidative coupling with Zr (IV) supported by a noninnocent anthracene-based ligand: Application to the catalytic cotrimerization of alkynes and nitriles to pyrimidines. *J. Am. Chem. Soc.* **2018,** *140* (38), 11906-11910.
(3) Neuwald, B.; Caporaso, L.; Cavallo, L.; Mecking, S., Concepts for stereoselective acrylate insertion. *J. Am. Chem. Soc.* **2013,** *135* (3), 1026-1036.
(4) Xiong, S.; Hong, A.; Bailey, B. C.; Spinney, H. A.; Senecal, T. D.; Bailey, H.; Agapie, T., Highly Active and Thermally Robust Nickel Enolate Catalysts for the Synthesis of Ethylene - Acrylate Copolymers. *Angew. Chem. Int. Ed.* **2022.**
(5) Palmer, W. N.; Zarate, C.; Chirik, P. J., Benzyltriboronates: building blocks for diastereoselective carbon–carbon bond formation. *J. Am. Chem. Soc.* **2017,** *139* (7), 2589-2592.
(6) Xiong, S.; Ghana, P.; Bailey, B. C.; Spinney, H. A.; Henderson, B. S.; Espinosa, M. R.; Agapie, T., Impact of Labile Ligands on Catalyst Initiation and Chain Propagation in Ni-Catalyzed Ethylene/Acrylate Copolymerization. *ACS Catal.* **2023,** *13* (7), 5000-5006.
(7) Falivene, L.; Credendino, R.; Poater, A.; Petta, A.; Serra, L.; Oliva, R.; Scarano, V.; Cavallo, L., SambVca 2. A web tool for analyzing catalytic pockets with topographic steric maps. *Organometallics* **2016,** *35* (13), 2286-2293.
(8) Falivene, L.; Cao, Z.; Petta, A.; Serra, L.; Poater, A.; Oliva, R.; Scarano, V.; Cavallo, L., Towards the online computer-aided design of catalytic pockets. *Nat. Chem.* **2019,** *11* (10), 872-879.
(9) Poater, A.; Cosenza, B.; Correa, A.; Giudice, S.; Ragone, F.; Scarano, V.; Cavallo, L., SambVca: A web application for the calculation of the buried volume of N-heterocyclic carbene ligands. Wiley Online Library: 2009.
(10) Ragone, F.; Poater, A.; Cavallo, L., Flexibility of N-heterocyclic carbene ligands in ruthenium complexes relevant to olefin metathesis and their impact in the first coordination sphere of the metal. *J. Am. Chem. Soc.* **2010,** *132* (12), 4249-4258.
(11) Xin, B. S.; Sato, N.; Tanna, A.; Oishi, Y.; Konishi, Y.; Shimizu, F., Nickel catalyzed copolymerization of ethylene and alkyl acrylates. *J. Am. Chem. Soc.* **2017,** *139* (10), 3611-3614.
(12) Xiong, S.; Shoshani, M. M.; Zhang, X.; Spinney, H. A.; Nett, A. J.; Henderson, B. S.; Miller III, T. F.; Agapie, T., Efficient Copolymerization of Acrylate and Ethylene with Neutral P, O-Chelated Nickel Catalysts: Mechanistic Investigations of Monomer Insertion and Chelate Formation. *J. Am. Chem. Soc.* **2021,** *143* (17), 6516-6527.
(13) Johnson, L. K.; Mecking, S.; Brookhart, M., Copolymerization of ethylene and propylene with functionalized vinyl monomers by palladium (II) catalysts. *J. Am. Chem. Soc.* **1996,** *118* (1), 267-268.
(14) Nakano, R.; Nozaki, K., Copolymerization of propylene and polar monomers using Pd/IzQO catalysts. *J. Am. Chem. Soc.* **2015,** *137* (34), 10934-10937.
(15) Takano, S.; Takeuchi, D.; Osakada, K.; Akamatsu, N.; Shishido, A., Dipalladium catalyst for olefin polymerization: Introduction of acrylate units into the main chain of branched polyethylene. *Angew. Chem. Int. Ed.* **2014,** *53* (35), 9246-9250.
(16) Zhang, Y.; Mu, H.; Wang, X.; Pan, L.; Li, Y., Elaborate tuning in ligand makes a big difference in catalytic performance: bulky nickel catalysts for (co) polymerization of ethylene with promising vinyl polar monomers. *ChemCatChem* **2019,** *11* (9), 2329-2340.

(17) Chen, M.; Chen, C., A Versatile Ligand Platform for Palladium-and Nickel-Catalyzed Ethylene Copolymerization with Polar Monomers. *Angew. Chem. Int. Ed.* **2018,** *57* (12), 3094-3098.
(18) Saki, Z.; D'Auria, I.; Dall'Anese, A.; Milani, B.; Pellecchia, C., Copolymerization of Ethylene and Methyl Acrylate by Pyridylimino Ni (II) Catalysts Affording Hyperbranched Poly (ethylene-co-methyl acrylate) s with Tunable Structures of the Ester Groups. *Macromolecules* **2020,** *53* (21), 9294-9305.
(19) Tahmouresilerd, B.; Xiao, D.; Do, L. H., Rigidifying Cation-Tunable Nickel Catalysts Increases Activity and Polar Monomer Incorporation in Ethylene and Methyl Acrylate Copolymerization. *Inorg. Chem.* **2021**.
(20) Tao, W. j.; Nakano, R.; Ito, S.; Nozaki, K., Copolymerization of ethylene and polar monomers by using Ni/IzQO catalysts. *Angew. Chem. Int. Ed.* **2016,** *55* (8), 2835-2839.
(21) Li, M.; Wang, X.; Luo, Y.; Chen, C., A Second-Coordination-Sphere Strategy to Modulate Nickel-and Palladium-Catalyzed Olefin Polymerization and Copolymerization. *Angew. Chem. Int. Ed.* **2017,** *56* (38), 11604-11609.
(22) Zhang, Y.; Mu, H.; Pan, L.; Wang, X.; Li, Y., Robust bulky [P, O] neutral nickel catalysts for copolymerization of ethylene with polar vinyl monomers. *ACS Catal.* **2018,** *8* (7), 5963-5976.
(23) Ye, J.; Mu, H.; Wang, Z.; Jian, Z., Heteroaryl backbone strategy in bisphosphine monoxide palladium-catalyzed ethylene polymerization and copolymerization with polar monomers. *Organometallics* **2019,** *38* (15), 2990-2997.
(24) Cui, L.; Jian, Z., A N-bridged strategy enables hemilabile phosphine–carbonyl palladium and nickel catalysts to mediate ethylene polymerization and copolymerization with polar vinyl monomers. *Polym. Chem* **2020,** *11* (38), 6187-6193.
(25) Zou, C.; Liao, D.; Pang, W.; Chen, M.; Tan, C., Versatile PNPO ligands for palladium and nickel catalyzed ethylene polymerization and copolymerization with polar monomers. *J. Catal.* **2021,** *393*, 281-289.
(26) Drent, E.; van Dijk, R.; van Ginkel, R.; van Oort, B.; Pugh, R. I., Palladium catalysed copolymerisation of ethene with alkylacrylates: polar comonomer built into the linear polymer chain. *Chem. Commun.* **2002,** (7), 744-745.
(27) Jenkins, J. C.; Brookhart, M., A mechanistic investigation of the polymerization of ethylene catalyzed by neutral Ni (II) complexes derived from bulky anilinotropone ligands. *J. Am. Chem. Soc.* **2004,** *126* (18), 5827-5842.
(28) Popeney, C. S.; Camacho, D. H.; Guan, Z., Efficient incorporation of polar comonomers in copolymerizations with ethylene using a cyclophane-based Pd (II) α-diimine catalyst. *J. Am. Chem. Soc.* **2007,** *129* (33), 10062-10063.
(29) Neuwald, B.; Ölscher, F.; Göttker-Schnetmann, I.; Mecking, S., Limits of activity: weakly coordinating ligands in arylphosphinesulfonato palladium (II) polymerization catalysts. *Organometallics* **2012,** *31* (8), 3128-3137.
(30) Piche, L.; Daigle, J. C.; Rehse, G.; Claverie, J. P., Structure–Activity Relationship of Palladium Phosphanesulfonates: Toward Highly Active Palladium-Based Polymerization Catalysts. *Chem. - Eur. J.* **2012,** *18* (11), 3277-3285.
(31) Wucher, P.; Goldbach, V.; Mecking, S., Electronic influences in phosphinesulfonato palladium (II) polymerization catalysts. *Organometallics* **2013,** *32* (16), 4516-4522.
(32) Jian, Z.; Wucher, P.; Mecking, S., Heterocycle-substituted phosphinesulfonato palladium (II) complexes for insertion copolymerization of methyl acrylate. *Organometallics* **2014,** *33* (11), 2879-2888.
(33) Ota, Y.; Ito, S.; Kuroda, J.-i.; Okumura, Y.; Nozaki, K., Quantification of the steric influence of alkylphosphine–sulfonate ligands on polymerization, leading to high-molecular-weight copolymers of

ethylene and polar monomers. *J. Am. Chem. Soc.* **2014,** *136* (34), 11898-11901.
(34) Pan, H.; Zhu, L.; Li, J.; Zang, D.; Fu, Z.; Fan, Z., A thermal stable α-diimine palladium catalyst for copolymerization of ethylene with functionalized olefins. *Journal of Molecular Catalysis A: Chemical* **2014,** *390*, 76-82.
(35) Zhu, L.; Fu, Z.-S.; Pan, H.-J.; Feng, W.; Chen, C.; Fan, Z.-Q., Synthesis and application of binuclear α-diimine nickel/palladium catalysts with a conjugated backbone. *Dalton Trans* **2014,** *43* (7), 2900-2906.
(36) Allen, K. E.; Campos, J. s.; Daugulis, O.; Brookhart, M., Living polymerization of ethylene and copolymerization of ethylene/methyl acrylate using "sandwich" diimine palladium catalysts. *ACS Catal.* **2015,** *5* (1), 456-464.
(37) Chen, M.; Yang, B.; Chen, C., Redox-Controlled Olefin (Co) Polymerization Catalyzed by Ferrocene-Bridged Phosphine-Sulfonate Palladium Complexes. *Angew. Chem. Int. Ed.* **2015,** *54* (51), 15520-15524.
(38) Dai, S.; Sui, X.; Chen, C., Highly Robust Palladium (II) α-Diimine Catalysts for Slow-Chain-Walking Polymerization of Ethylene and Copolymerization with Methyl Acrylate. *Angew. Chem. Int. Ed.* **2015,** *54* (34), 9948-9953.
(39) Dai, S.; Chen, C., Direct synthesis of functionalized high-molecular-weight polyethylene by copolymerization of ethylene with polar monomers. *Angew. Chem. Int. Ed.* **2016,** *55* (42), 13281-13285.
(40) Hu, H.; Chen, D.; Gao, H.; Zhong, L.; Wu, Q., Amine–imine palladium catalysts for living polymerization of ethylene and copolymerization of ethylene with methyl acrylate: incorporation of acrylate units into the main chain and branch end. *Polym. Chem* **2016,** *7* (3), 529-537.
(41) Mitsushige, Y.; Carrow, B. P.; Ito, S.; Nozaki, K., Ligand-controlled insertion regioselectivity accelerates copolymerisation of ethylene with methyl acrylate by cationic bisphosphine monoxide–palladium catalysts. *Chem. Sci.* **2016,** *7* (1), 737-744.
(42) Wang, R.; Zhao, M.; Chen, C., Influence of ligand second coordination sphere effects on the olefin (co) polymerization properties of α-diimine Pd (II) catalysts. *Polym. Chem* **2016,** *7* (23), 3933-3938.
(43) Wu, Z.; Chen, M.; Chen, C., Ethylene polymerization and copolymerization by palladium and nickel catalysts containing naphthalene-bridged phosphine–sulfonate ligands. *Organometallics* **2016,** *35* (10), 1472-1479.
(44) Zou, W.; Chen, C., Influence of backbone substituents on the ethylene (co) polymerization properties of α-diimine Pd (II) and Ni (II) catalysts. *Organometallics* **2016,** *35* (11), 1794-1801.
(45) Liang, T.; Chen, C., Side-arm control in phosphine-sulfonate palladium-and nickel-catalyzed ethylene polymerization and copolymerization. *Organometallics* **2017,** *36* (12), 2338-2344.
(46) Sui, X.; Hong, C.; Pang, W.; Chen, C., Unsymmetrical α-diimine palladium catalysts and their properties in olefin (co) polymerization. *Mater. Chem. Front.* **2017,** *1* (5), 967-972.
(47) Tao, W.; Akita, S.; Nakano, R.; Ito, S.; Hoshimoto, Y.; Ogoshi, S.; Nozaki, K., Copolymerisation of ethylene with polar monomers by using palladium catalysts bearing an N-heterocyclic carbene–phosphine oxide bidentate ligand. *Chem. Commun.* **2017,** *53* (17), 2630-2633.
(48) Wu, Z.; Hong, C.; Du, H.; Pang, W.; Chen, C., Influence of ligand backbone structure and connectivity on the properties of phosphine-sulfonate Pd (II)/Ni (II) catalysts. *Polymers* **2017,** *9* (5), 168.
(49) Yang, B.; Xiong, S.; Chen, C., Manipulation of polymer branching density in phosphine-sulfonate palladium and nickel catalyzed ethylene polymerization. *Polym. Chem* **2017,** *8* (40), 6272-6276.
(50) Zhai, F.; Solomon, J. B.; Jordan, R. F., Copolymerization of ethylene with acrylate monomers by amide-functionalized α-diimine Pd catalysts. *Organometallics* **2017,** *36* (9), 1873-1879.

(51) Zhang, D.; Chen, C., Influence of Polyethylene Glycol Unit on Palladium - and Nickel - Catalyzed Ethylene Polymerization and Copolymerization. *Angew. Chem. Int. Ed.* **2017,** *56* (46), 14672-14676.
(52) Zhao, M.; Chen, C., Accessing multiple catalytically active states in redox-controlled olefin polymerization. *ACS Catal.* **2017,** *7* (11), 7490-7494.
(53) Zhong, S.; Tan, Y.; Zhong, L.; Gao, J.; Liao, H.; Jiang, L.; Gao, H.; Wu, Q., Precision synthesis of ethylene and polar monomer copolymers by palladium-catalyzed living coordination copolymerization. *Macromolecules* **2017,** *50* (15), 5661-5669.
(54) Cai, Z.; Do, L. H., Thermally Robust Heterobimetallic Palladium–Alkali Catalysts for Ethylene and Alkyl Acrylate Copolymerization. *Organometallics* **2018,** *37* (21), 3874-3882.
(55) Ding, L.; Cheng, H.; Li, Y.; Tanaka, R.; Shiono, T.; Cai, Z., Efficient ethylene copolymerization with polar monomers using palladium anilinonaphthoquinone catalysts. *Polym. Chem* **2018,** *9* (45), 5476-5482.
(56) Guo, L.; Liu, Y.; Sun, W.; Du, Q.; Yang, Y.; Kong, W.; Liu, Z.; Chen, D., Synthesis, characterization, and olefin (co) polymerization behavior of unsymmetrical α-diimine palladium complexes containing bulky substituents at 4-position of aniline moieties. *J. Organomet. Chem.* **2018,** *877*, 12-20.
(57) Zhang, W.; Waddell, P. M.; Tiedemann, M. A.; Padilla, C. E.; Mei, J.; Chen, L.; Carrow, B. P., Electron-rich metal cations enable synthesis of high molecular weight, linear functional polyethylenes. *J. Am. Chem. Soc.* **2018,** *140* (28), 8841-8850.
(58) Du, C.; Zhong, L.; Gao, J.; Zhong, S.; Liao, H.; Gao, H.; Wu, Q., Living (co) polymerization of ethylene and bio-based furfuryl acrylate using dibenzobarrelene derived α-diimine palladium catalysts. *Polym. Chem* **2019,** *10* (16), 2029-2038.
(59) Liao, Y.; Zhang, Y.; Cui, L.; Mu, H.; Jian, Z., Pentiptycenyl substituents in insertion polymerization with α-diimine nickel and palladium species. *Organometallics* **2019,** *38* (9), 2075-2083.
(60) Li, K.; Ye, J.; Wang, Z.; Mu, H.; Jian, Z., Indole-bridged bisphosphine-monoxide palladium catalysts for ethylene polymerization and copolymerization with polar monomers. *Polym. Chem* **2020**.
(61) Li, S.; Dai, S., 8-Arylnaphthyl substituent retarding chain transfer in insertion polymerization with unsymmetrical α-diimine systems. *Polym. Chem* **2020,** *11* (45), 7199-7206.
(62) Park, D.-A.; Byun, S.; Ryu, J. Y.; Lee, J.; Lee, J.; Hong, S., Abnormal N-Heterocyclic Carbene–Palladium Complexes for the Copolymerization of Ethylene and Polar Monomers. *ACS Catal.* **2020,** *10* (10), 5443-5453.
(63) Tan, C.; Qasim, M.; Pang, W.; Chen, C., Ligand–metal secondary interactions in phosphine–sulfonate palladium and nickel catalyzed ethylene (co) polymerization. *Polym. Chem* **2020,** *11* (2), 411-416.
(64) Xu, M.; Yu, F.; Li, P.; Xu, G.; Zhang, S.; Wang, F., Enhancing chain initiation efficiency in the cationic allyl-nickel catalyzed (co) polymerization of ethylene and methyl acrylate. *Inorg. Chem.* **2020,** *59* (7), 4475-4482.
(65) Zhang, Y.; Wang, C.; Mecking, S.; Jian, Z., Ultrahigh branching of main - chain - functionalized polyethylenes by inverted insertion selectivity. *Angew. Chem.* **2020,** *132* (34), 14402-14408.
(66) Zhong, L.; Zheng, H.; Du, C.; Du, W.; Liao, G.; Cheung, C. S.; Gao, H., Thermally robust α-diimine nickel and palladium catalysts with constrained space for ethylene (co) polymerizations. *J. Catal.* **2020,** *384*, 208-217.
(67) Zhou, G.; Mu, H.; Jian, Z., A comprehensive picture on catalyst structure construction in palladium catalyzed ethylene (co) polymerizations. *J. Catal.* **2020,** *383*, 215-220.
(68) Akita, S.; Nozaki, K., Copolymerization of ethylene and methyl acrylate by palladium catalysts

bearing IzQO ligands containing methoxyethyl ether moieties and salt effects for polymerization. *Polymer Journal* **2021,** *53* (9), 1057-1060.
(69) Chen, S.-Y.; Pan, R.-C.; Liu, Y.; Lu, X.-B., Bulky o-Phenylene-Bridged Bimetallic α-Diimine Ni (II) and Pd (II) Catalysts in Ethylene (Co) polymerization. *Organometallics* **2021,** *40* (22), 3703-3711.
(70) Dai, S.; Li, G.; Lu, W.; Liao, Y.; Fan, W., Suppression of chain transfer via a restricted rotation effect of dibenzosuberyl substituents in polymerization catalysis. *Polym. Chem* **2021,** *12* (22), 3240-3249.
(71) Ge, Y.; Li, S.; Fan, W.; Dai, S., Flexible "Sandwich"(8-Alkylnaphthyl α-Diimine) Catalysts in Insertion Polymerization. *Inorg. Chem.* **2021,** *60* (8), 5673-5681.
(72) Ge, Y.; Li, S.; Wang, H.; Dai, S., Synthesis of Branched Polyethylene and Ethylene-MA Copolymers Using Unsymmetrical Iminopyridyl Nickel and Palladium Complexes. *Organometallics* **2021,** *40* (17), 3033-3041.
(73) Guo, L.; Hu, X.; Lu, W.; Xu, G.; Liu, Q.; Dai, S., Investigations of ligand backbone effects on bulky diarylmethyl-based nickel (II) and palladium (II) catalyzed ethylene polymerization and copolymerization. *J. Organomet. Chem.* **2021,** *952*, 122046.
(74) Hai, Z.; Lu, Z.; Li, S.; Cao, Z.-Y.; Dai, S., The synergistic effect of rigid and flexible substituents on insertion polymerization with α-diimine nickel and palladium catalysts. *Polym. Chem* **2021,** *12* (32), 4643-4653.
(75) Li, S.; Dai, S., Highly efficient incorporation of polar comonomers in copolymerizations with ethylene using iminopyridyl palladium system. *J. Catal.* **2021,** *393*, 51-59.
(76) Liu, Y. S.; Harth, E., Distorted Sandwich α-Diimine PdII Catalyst: Linear Polyethylene and Synthesis of Ethylene/Acrylate Elastomers. *Angew. Chem. Int. Ed.* **2021**.
(77) Lu, W.; Xu, G.; Chang, G.; Wang, H.; Dai, S., Synthesis of highly branched polyethylene and ethylene-MA copolymers using hybrid bulky α-diimine Pd (II) catalysts. *J. Organomet. Chem.* **2021,** *956*, 122118.
(78) Wang, G.; Peng, D.; Sun, Y.; Chen, C., Interplay of supramolecular chemistry and photochemistry with palladium-catalyzed ethylene polymerization. *CCS Chemistry* **2021,** *3* (7), 2025-2034.
(79) Wang, X.-l.; Zhang, Y.-p.; Wang, F.; Pan, L.; Wang, B.; Li, Y.-s., Robust and Reactive Neutral Nickel Catalysts for Ethylene Polymerization and Copolymerization with a Challenging 1,1-Disubstituted Difunctional Polar Monomer. *ACS Catal.* **2021**, 2902-2911.
(80) Xia, J.; Han, Y.-F.; Kou, S.; Zhang, Y.; Jian, Z., Exploring steric effect of electron-donating group in palladium and nickel mediated ethylene polymerization and copolymerization with polar monomers. *European Polymer Journal* **2021,** *160*, 110781.
(81) Zhu, N.; Liang, T.; Huang, Y.; Pang, W.; Chen, M.; Tan, C., Influences of ligand backbone substituents on phosphinecarbonylpalladium and-nickel catalysts for ethylene polymerization and copolymerization with polar monomers. *Inorg. Chem.* **2021,** *60* (17), 13080-13090.
(82) Alberoni, C.; D'Alterio, M. C.; Balducci, G.; Immirzi, B.; Polentarutti, M.; Pellecchia, C.; Milani, B., Tunable "In-Chain" and "At the End of the Branches" Methyl Acrylate Incorporation in the Polyolefin Skeleton through Pd (II) Catalysis. *ACS Catal.* **2022,** *12* (6), 3430-3443.
(83) Shoshani, M. M.; Xiong, S.; Lawniczak, J. J.; Zhang, X.; Miller, T. F.; Agapie, T., Phosphine-Phenoxide Nickel Catalysts for Ethylene/Acrylate Copolymerization: Olefin Coordination and Complex Isomerization Studies Relevant to the Mechanism of Catalysis. *Organometallics* **2022**.
(84) Agapie, T.; Xiong, S.; Bailey, B. C.; Spinney, H. A.; Nett, A. J.; Wilson, D. R.; Klosin, J., Bis (phosphino)-phenoxy nickel (ii) catalysts for the copolymerization of ethylene and acrylate monomers. Google Patents: 2022.
(85) Cui, L.; Chu, Y.-K.; Liu, D.-J.; Han, Y.-F.; Mu, H.-L.; Jian, Z.-B., Enhancement on Hemilabile

Phosphine-Amide Palladium and Nickel Catalysts for Ethylene (Co)Polymerization with Polar Monomers Using a Cyclizing Strategy. *Chinese Journal of Polymer Science* **2022,** *40* (3), 241-247.
(86) Wang, X. l.; Zhang, Y. P.; Pan, L.; Wang, F.; Luo, S. y.; Li, Y. s., Reactivity of Phosphino-naphtholate Nickel Complexes and Their Catalysis of Copolymerization with Polar Monomers. *ChemCatChem* **2022,** *14* (5), e202101736.
(87) Yan, Z.; Xu, G.; Wang, H.; Dai, S., Synthesis of thermoplastic polyethylene elastomers and ethylene–methyl acrylate copolymers using methylene-bridged binuclear bulky dibenzhydryl α-diimine Ni (II) and Pd (II) catalysts. *European Polymer Journal* **2022,** *168*, 111105.
(88) Ma, X.; Zhang, Y.; Jian, Z., Unsymmetrical Strategy on α-Diimine Nickel and Palladium Mediated Ethylene (Co) Polymerizations. *Molecules* **2022,** *27* (24), 8942.
(89) Ji, M.; Si, G.; Pan, Y.; Tan, C.; Chen, M., Polymeric α-diimine palladium catalysts for olefin (co) polymerization. *J. Catal.* **2022,** *415*, 51-57.
(90) Zheng, H.; Pei, L.; Deng, H.; Gao, H.; Gao, H., Electronic effects of amine-imine nickel and palladium catalysts on ethylene (co) polymerization. *European Polymer Journal* **2023,** *184*, 111773.
(91) Wang, H.; Lu, Z.; Dai, S., Synthesis of Hyperbranched Polyethylene and Carboxylate Functionalized Polyethylene Using Bis (benzocyclopentyl) α-Diimine Pd (II) Catalysts with Axial Substituent-Constrained Strategy. *European Polymer Journal* **2023**, 112247.
(92) Ding, B.; Jiang, L.; Kang, X.; Dai, S., Enhancing Suppression of Chain Transfer via Catalyst Structural Evolution in Ethylene (Co) Polymerization. *Chinese Journal of Chemistry* **2023,** *41* (12), 1509-1516.
(93) Lu, W.; Fan, W.; Dai, S., A rigid–flexible double-layer steric strategy promoting ethylene polymerization and copolymerization in alkane solvents. *Inorganic Chemistry Frontiers* **2023,** *10* (1), 108-117.
(94) Cao, L.; Cai, Z.; Li, M., Synthesis and Characterization of Phosphinobenzenamine Palladium Complexes and Their Application in Ethylene Polymerization and Copolymerization with Polar Monomers. *Organometallics* **2022,** *41* (23), 3538-3545.
(95) Liao, Y.-D.; Cai, Q.; Dai, S.-Y., Synthesis of high molecular weight polyethylene and E-MA copolymers using iminopyridine Ni (II) and Pd (II) complexes containing a flexible backbone and rigid axial substituents. *Chinese Journal of Polymer Science* **2023,** *41* (2), 233-239.
(96) Lu, W.; Liao, Y.; Dai, S., Facile access to ultra-highly branched polyethylenes using hybrid "sandwich" Ni (II) and Pd (II) catalysts. *J. Catal.* **2022,** *411*, 54-61.
(97) Lu, Z.; Liao, Y.; Fan, W.; Dai, S., Efficient suppression of the chain transfer reaction in ethylene coordination polymerization with dibenzosuberyl substituents. *Polym. Chem* **2022,** *13* (28), 4090-4099.
(98) Fan, H.; Chang, G.; Wang, H.; Dai, S., The second coordination sphere effect of benzothiophene groups enhances chain transfer in ethylene (co) oligomerization. *New Journal of Chemistry* **2023,** *47* (5), 2488-2494.
(99) Zou, C.; Si, G.; Chen, C., A general strategy for heterogenizing olefin polymerization catalysts and the synthesis of polyolefins and composites. *Nat. Commun.* **2022,** *13* (1), 1-12.
(100) Lalwani, N.; Allen, D. W.; Horton, P. N.; Coles, S. J.; Cross, N. A.; Bricklebank, N., Methoxy-phenyl groups reduce the cytotoxicity and increase the aqueous solubility of phosphonium zwitterions and salts. *Polyhedron* **2019,** *158*, 515-523.
(101) Rünzi, T.; Tritschler, U.; Roesle, P.; Göttker-Schnetmann, I.; Möller, H. M.; Caporaso, L.; Poater, A.; Cavallo, L.; Mecking, S., Activation and deactivation of neutral palladium (II) phosphinesulfonato polymerization catalysts. *Organometallics* **2012,** *31* (23), 8388-8406.
